# Updated profiling of COVID-19 vaccine adverse events using VAERS case reports

**DOI:** 10.3389/fphar.2026.1741967

**Published:** 2026-03-26

**Authors:** Anna He, Katelyn Hur, Xingxian Li, Jie Zheng, Junguk Hur, Yongqun He

**Affiliations:** 1 Huron High School, Ann Arbor, MI, United States; 2 Red River High School, Grand Forks, ND, United States; 3 College of Literature, Science, and the Arts, University of Michigan, Ann Arbor, MI, United States; 4 Unit for Laboratory Animal Medicine, University of Michigan Medical School, Ann Arbor, MI, United States; 5 Department of Biomedical Sciences, University of North Dakota, School of Medicine and Health Sciences, Grand Forks, ND, United States; 6 Center of Computational Medicine and Bioinformatics, University of Michigan Medical School, Ann Arbor, MI, United States; 7 Department of Learning Health Science, University of Michigan Medical School, Ann Arbor, MI, United States

**Keywords:** adverse events, case reports, COVID-19 vaccine, Guillain-Barré syndrome, myocarditis, ontology of adverse events, thrombosis, VAERS

## Abstract

**Background:**

Adverse events (AEs) associated with COVID-19 vaccines remain a critical aspect of safety surveillance. In 2022, we reported the first systematic profiling of COVID-19 vaccine AEs using the Vaccine Adverse Event Reporting System (VAERS). Since then, vaccines have evolved with the introduction of bivalent formulations. This study provides an updated analysis to capture evolving safety trends.

**Methods:**

Building upon our previous analysis, we systematically analyzed AE profiles for the Pfizer-BioNTech, Moderna, and Janssen vaccines, along with the newer bivalent Pfizer-BioNTech and Moderna vaccines and the protein subunit Novavax vaccine, using VAERS data through 28 June 2024. We obtained processed VAERS data via Cov19VaxKB. Significance of each AE was determined using Pearson’s Chi-square test, proportional reporting ratios, and case report frequencies with established thresholds. Overlap and age- or sex-stratified analyses were conducted to characterize shared and unique AE patterns across vaccine types. AE classification using the Ontology of Adverse Events was performed to categorize and interpret significant AEs within a structured hierarchy.

**Results:**

We observed a marked decrease in unique AEs reported for the Pfizer-BioNTech and Moderna monovalent mRNA vaccines and the recombinant vector vaccine Janssen. The bivalent versions of Pfizer-BioNTech and Moderna exhibited distinct overlapping AE profiles compared to their monovalent counterparts, and bivalent vaccines were generally associated with fewer AEs than the classical monovalent vaccines. Significant differences were observed in thrombosis, myocarditis, and Guillain-Barré syndrome (GBS) across vaccines. Age-specific analyses revealed a bimodal pattern with higher AE reporting in children aged 0–9 and adults aged 50–69, and clear sex differences. Females reported more common AEs, while males were more often linked to serious AEs (thrombosis, myocarditis, and GBS). Death-related AEs were uncommon but more frequent among older males and primarily associated with monovalent formulations. Ontology-based classification revealed that females were more likely to experience sensory-related AEs, whereas males were more prone to cardiovascular-related AEs.

**Conclusion:**

The adverse event profiles of COVID-19 vaccines during 2020–2024 largely overlapped those identified during 2020–2021, while also revealing new overall and age- and sex-specific AE patterns. Ontology-guided classification enhanced the interpretation of large-scale vaccine safety data, supporting more precise risk assessment across groups.

## 1 Introduction

COVID-19 vaccines have had a significant impact on public health since their introduction in 2020. According to the WHO, as of 16 August 2025, the number of cases attributed to COVID-19 was over 778 million, and more than 7.1 million deaths were reported worldwide (WHO, 2024). Attribution of COVID-19-associated deaths in large-scale surveillance systems is subject to methodological considerations, including classification and case-counting biases (Alessandria et al., 2025).

Multiple COVID-19 vaccines have been developed and deployed over the past several years. Among them, monovalent Pfizer-BioNTech (Comirnaty) and Moderna (Spikevax) (Meo et al., 2021) vaccines were the most widely used between December 2020 and April 2023 (Oliver et al., 2024). The Janssen (Johnson & Johnson) vaccine was administered between February 2021 and May 2023, and it was withdrawn on 1 June 2023, by the United States Food and Drug Administration (FDA) (FDA, 2023). Since August 2022, bivalent versions of Pfizer-BioNTech and Moderna vaccines have also been in use (Rosenblum et al., 2022). Comirnaty and Spikevax are based on mRNA technology, while the Janssen vaccine employs a recombinant viral vector platform. In addition, the adjuvanted Novavax COVID-19 vaccine was authorized for emergency use by the U.S. FDA for individuals aged 12 years and older since August 2022 (Larkin, 2022; Fix et al., 2024).

COVID-19 vaccines have been associated with a range of adverse events (AEs) (Rosenblum et al., 2021; Yasmin et al., 2023). These AEs include serious conditions that may lead to hospitalization, life-threatening illness, or death (Diaz et al., 2021; Woo et al., 2021; Patone et al., 2022; Xu et al., 2024). Numerous studies have examined vaccine safety using diverse data sources (Newbern et al., 2025), and many types of AEs have been reported during the global COVID-19 vaccine campaign. For example, thrombosis with thrombocytopenia syndrome (TTS) was significantly associated with the Janssen vaccine (Marcucci and Marietta, 2021), a finding that contributed to its discontinuation. Meanwhile, other vaccines were also reported to cause other concerning AEs, such as myocarditis linked to the mRNA-based Pfizer-BioNTech Comirnaty (i.e., BNT162b2) and Moderna Spikevax (i.e., mRNA-1273) (Patrignani et al., 2021; Ahmed, 2022; Goddard et al., 2022; Naveed et al., 2022; Wong et al., 2022). Guillain-Barré syndrome (GBS) has also been identified as a severe side effect of COVID-19 vaccines (Sadowski et al., 2025). Indeed, these three AEs (myocarditis, thrombosis, and GBS) have been consistently recognized as key concerns in post-vaccine safety monitoring (Guo et al., 2022) and warned about by the CDC and FDA (MacNeil et al., 2021; Oliver et al., 2022; CDC, 2025; Pan et al., 2025). Meanwhile, many other severe AEs such as acute kidney injury, seizure, and death have been frequently reported and studied (Pan et al., 2024; CDC, 2025; Pan et al., 2025).

Using the Vaccine Adverse Event Reporting System (VAERS) (Dayan et al., 2005), we previously reported COVID-19 vaccine AE profiles using VAERS data up to the end of December 2021 (Guo et al., 2022). That systematic study identified 96 AEs that were statistically significantly associated with the monovalent Pfizer-BioNTech, Moderna, and Janssen COVID-19 vaccines. Among them, the Janssen vaccine showed a higher crude reporting rate of AEs, and females accounted for a greater proportion of reports for many common AEs. Myocarditis was more strongly associated with the monovalent Pfizer-BioNTech vaccine, while thrombosis and GBS were more strongly associated with the Janssen vaccine. These findings established the baseline for the current extended analysis that incorporates newer vaccine formulations and updated VAERS data through mid-2024.

Since the end of 2021, substantial changes in COVID-19 vaccine availability, formulation, and usage patterns have occurred. In this study, we present an updated analysis of COVID-19 vaccine AE profiles using VAERS data through June 2024. Our findings indicate that the updated monovalent Pfizer-BioNTech and Moderna vaccines demonstrated fewer AEs and fewer severe AEs compared to the earlier formulations. We also report AE profiles for the newer Pfizer-BioNTech and Moderna bivalent vaccines and the Novavax vaccine, providing an updated view of COVID-19 vaccine safety.

## 2 Methods

### 2.1 VAERS data extraction and processing

All VAERS case report data up to 28 June 2024, were downloaded from the VAERS database (Dayan et al., 2005), processed, and stored in a MySQL database, which was further used in the development of the web-based COVID-19 Vaccine Knowledge Base (Cov19VaxKB) (Huang et al., 2021). Cov19VaxKB conditional and statistical web query programs were used to extract vaccine AE data based on criteria such as sex and age group. Age groups were binned as 0–9, 10–19, 20–29, 30–39, 40–49, 50–59, 60–69, 70–79, and 80+, and records with missing age or sex were retained and categorized as “unknown.” Vaccine manufacturer and formulation were harmonized into six vaccine categories: Pfizer-BioNTech monovalent, Moderna monovalent, Janssen, Pfizer-BioNTech bivalent, Moderna bivalent, and Novavax.

The VAERS AE records were coded using the MedDRA system (Brown et al., 1999). However, some MedDRA terms do not represent true AEs, such as *white blood cell count normal*, *rheumatoid factor negative*, or *expired product administered*. These terms reflect normal findings or administrative or procedural concepts rather than AEs. Therefore, we employed an updated term-trimming process to remove these non-AE vocabulary terms, building on our previously described method (Zi et al., 2022). Briefly, trimming was guided by the Ontology of Adverse Events (OAE), and MedDRA terms were removed only if they were not classified as adverse events under OAE definitions. The removed terms fell into well-defined categories, including normal or negative clinical findings, diagnostic or monitoring procedures (e.g., laboratory tests, imaging, and physical examinations), medical or therapeutic interventions, and vaccine administration or product-related issues.

### 2.2 Statistical analysis

Three statistical data analysis metrics, including Pearson’s Chi-square statistic without Yates’s correction, Proportional Reporting Ratio (PRR) (Evans et al., 2001), and case report frequency (Sarntivijai et al., 2012), were calculated for each AE as previously defined (Guo et al., 2022). Case report frequency was calculated as the number of case reports for a specific AE associated with a given vaccine, divided by the total number of AE reports for the vaccine (Sarntivijai et al., 2012). PRR was calculated as the proportion of reports with the specific AE for the vaccine of interest divided by the proportion of reports with the same AE for all other vaccines combined. To define whether an AE was statistically significantly associated with a specific vaccine, the following criteria were required: (1) the total number of case reports for the vaccine ≥3, (2) a case report frequency >0.2%, (3) PRR >2, and (4) Chi-square statistic >4 (Sarntivijai et al., 2012).

The extracted AE information was compiled into Venn diagrams and summary tables to analyze AE profiles of COVID-19 vaccines over the entire period of vaccine use since 2020. This study analyzed AE profiles of COVID-19 vaccines over the entire period of vaccine use since 2020, and the results were compared with the earlier report that covered only the 2020–2021 period (Guo et al., 2022).

Furthermore, the effects of different variables, including sex (male, female, and unknown) and age bins listed above, on AE occurrence were investigated using the Cov19VaxKB (Huang et al., 2021; Guo et al., 2022) web interface and processed by custom R scripts for data processing. The three selected concerning AEs (i.e., myocarditis, GBS, and thrombosis) were also subjected to targeted comparative analyses.

For age- and sex-stratified analyses, AE case report frequencies were calculated within each vaccine-specific age and sex subgroup as the proportion of reports for a given AE relative to the total reports in that subgroup. To reduce instability in percentage estimates caused by sparse data, vaccine-by-subgroup strata (age and sex) with fewer than 100 total reports were excluded from stratified analyses.

### 2.3 AE overlap analysis

To assess the overlap and uniqueness of the identified AEs across different COVID-19 vaccines over time, overlap analyses were performed using the R packages VennDetail and UpSetR, across the following vaccines: Pfizer-BioNTech monovalent, Moderna monovalent, Janssen, Pfizer-BioNTech bivalent, Moderna bivalent, and Novavax. However, because Novavax had a very small number of AE reports, it was excluded from Venn diagram analyses and other inferential comparisons.

For pairwise comparisons, Venn diagrams were generated to illustrate shared and unique AEs among the monovalent vaccines and between the monovalent and bivalent formulations of Pfizer-BioNTech and Moderna. UpSet plots were used to visualize broader comparisons among all five vaccines, enabling a clearer representation of common and distinct AE profiles. The compiled results provided insights into how AE patterns evolved over time and across vaccine types.

### 2.4 Ontology-based AE classification and modeling

The lists of statistically significant vaccine AEs identified in our study were mapped to the Ontology of Adverse Events (OAE) (He et al., 2014) and then classified using the OAE AE hierarchy as reported earlier (Sarntivijai et al., 2012). After the term mapping, the OntoFox tool (Xiang et al., 2010) was used to extract each mapped OAE AE term and its semantically related concepts within OAE. The resulting ontology subset was visualized in the Protégé-OWL editor (Musen and Protege, 2015) to display the hierarchical structure of the mapped AE terms and their related terms in the OAE. Ontology mapping was performed after the MedDRA trimming step to avoid propagating non-AE concepts.

### 2.5 Software and reproducibility

Data processing and visualization were conducted using MySQL for data storage, R for statistical analysis and plotting, and in-house Python and R scripts for automation. The specific packages included VennDetail and UpSetR for overlap visualizations. Aggregated output tables and analysis scripts, including figure generation, are available in the Supplementary Material and in a GitHub repository (see Data Availability Statement).

### 2.6 Ethics and data use

VAERS data are publicly available and de-identified. Analyses of public, de-identified data are not human subjects research; therefore, institutional review board review was not required.

## 3 Results

**FIGURE 1:**
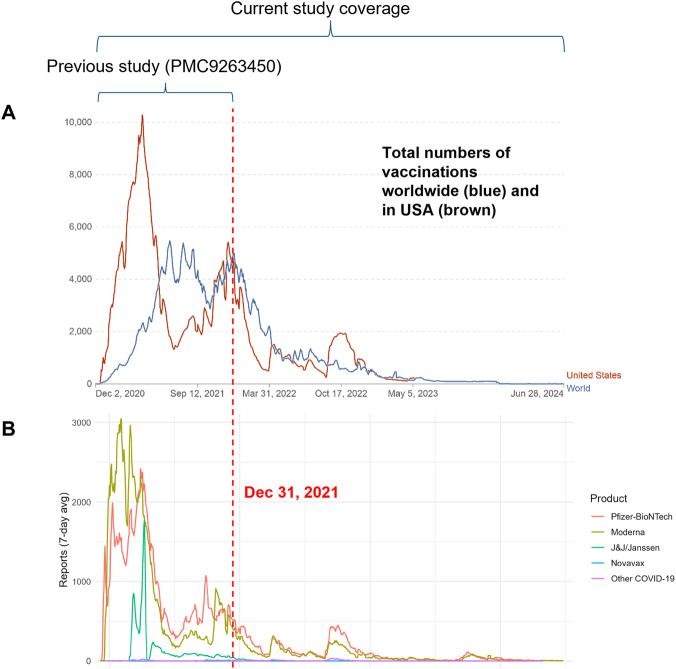
COVID-19 vaccination and VAERS reporting trends over time. **(A)** Daily COVID-19 vaccine doses administered per million people in the United States (brown) and world (blue). Data were extracted from Our World in Data (https://ourworldindata.org/covid-vaccinations). **(B)** Frequency of COVID-19 vaccine-related VAERS reports over the same period. Data were obtained from Cov19VaxKB using VAERS data.

Figure 1 summarizes the trends in COVID-19 vaccination rates and VAERS reporting frequencies from December 2020 through June 2024 (Mathieu et al., 2020). The global and U.S. vaccination rates (Figure 1A) peaked in 2021 and then gradually declined in the following years. The number of VAERS case reports (Figure 1B) followed a similar pattern, with a sharp peak in 2021, a decrease in 2022, and further reductions in 2023 and early 2024. This alignment between vaccination activity and reporting frequency provides context for the vaccine-specific AE profiles presented in the following sections.

### 3.1 General profiles of COVID-19 vaccine adverse event case reports between the periods of 2020–2021 and 2020–2024

Table 1 compares the statistics of COVID-19 vaccine-associated AE case reports during the two study periods of 2020–2021 and 2020–2024. As of 28 June 2024, the VAERS database included a total of 970,968 AE case reports for all FDA-authorized or approved classic COVID-19 vaccines, including 453,010 reports for the Pfizer-BioNTech Monovalent COVID-19 vaccine, 436,071 reports for the Moderna Monovalent COVID-19 vaccine, 74,108 reports for the Janssen COVID-19 vaccine, and 7,302 reports for COVID-19 vaccines with unknown manufacturers. Additionally, the new dataset includes cases for three new COVID-19 vaccines, specifically, 23,575 cases for the Pfizer-BioNTech bivalent vaccine, 17,369 cases for the Moderna bivalent vaccine, and 477 cases for the Novavax vaccine. Because the number of Novavax vaccine-associated AE cases, which began in September 2022, was very small, meaningful statistical analyses were not feasible; therefore, they were excluded from inferential analyses but retained for descriptive summaries.

**TABLE 1:** Number of reported COVID-19 vaccine AE cases in VAERS.

Vaccines	2000 - December 2021	2000 - June 2024	2020^a^	2021	2022	2023	2024^b^
Pfizer (monovalent)	323,185	453,010	7,719	315,466	101,300	23,045	5,479
Pfizer (Bivalent)	N/A	23,575	N/A	N/A	8,853	14,142	580
Moderna (monovalent)	329,056	436,071	3,124	325,932	83,509	20,441	3,063
Moderna (bivalent)	N/A	17,369	N/A	N/A	6,695	10,094	580
Janssen	63,741	74,108	N/A	63,741	8,711	1,422	234
Novavax	N/A	477	N/A	N/A	200	207	70
Unknown	1,595	7,302	21	1,574	3,368	2,086	253
Total (Bivalent only)	717,577	970,968	10,864	706,713	197,088 (15,548)	47,201 (24,236)	9,099 (1,160)

^a^ 2020 included reports collected in December 2020.

^b^ 2024 included reports collected from 1 January 2024, through 28 June 2024. N/A: not available.

The last five columns in Table 1 compare the numbers of case reports for each year from 2020 to 2024. Annual total reports reached their peak in 2021, followed by a small surge in 2022. Initially, most case reports came from Pfizer-BioNTech’s monovalent vaccines when the COVID-19 vaccines were first released in December 2020. In 2021, the number of case reports for Pfizer-BioNTech monovalent and Moderna monovalent vaccines increased sharply, while the number for the Janssen vaccine remained lower. In 2022, the number of case reports for monovalent vaccines decreased by roughly one-third, and Janssen case reports dropped even more significantly. Meanwhile, the number of bivalent Pfizer-BioNTech and Moderna vaccine case reports began to rise. By 2023, case reports for monovalent vaccines fell to around 20,000, with bivalent vaccines stabilizing at approximately 10,000.

The trends in AE case reporting closely mirrored the vaccination activity (Figure 1). For example, 2021, the year with the highest COVID-19 vaccine dosage rate per 1 million people in the U.S., also had the highest number of VAERS reports (Table 1). During the first half of 2024, only 9,099 AE case reports were recorded, representing a substantial decline compared with previous years.

### 3.2 Fewer statistically significant AE signals of COVID-19 monovalent vaccines during 2020–2024 compared with 2020–2021

**FIGURE 2:**
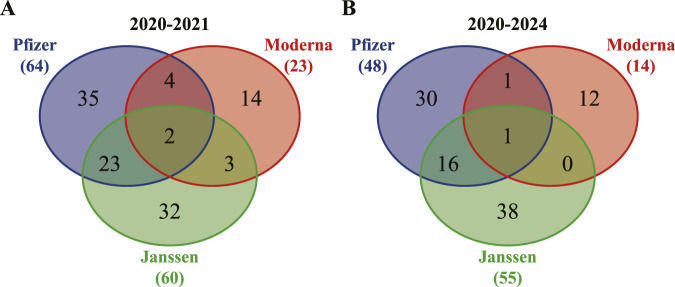
Venn diagrams of significant VAE types for three monovalent classic COVID-19 vaccines in December 2020 - December 2021 and December 2020 - June 2024. The three classic vaccines are monovalent Pfizer, Moderna, and Janssen vaccines. **(A)** A total of 113 significantly enriched VAE terms during 2020–2021. **(B)** A total of 98 significantly enriched VAE terms during 2020–2024. The criteria for significance selection included the total number of case reports for the vaccine ≥3, case report frequency >0.2%, PRR >2, and Chi-square statistic >4. Only trimmed versions after removing non-medically relevant VAEs were used in the analysis (the same in other analyses later).

Since the only three classic COVID-19 vaccines in use before the end of 2021 were monovalent Pfizer-BioNTech, Moderna, and Janssen vaccines, we first compared the statistically significant AE profiles associated with these vaccines during these two study periods (2020–2021 and 2020–2024). Using the criteria specified in Methods, we identified fewer shared statistically significant AEs associated during 2020–2024 than during 2020–2021. Specifically, there were 113 AE terms during 2020–2021 (Figure 2A) in the trimmed version (i.e., after removing non-medically relevant VAEs), compared with 98 AE terms during 2020–2024 (Figure 2B). Supplementary Materials S1,S2 include both untrimmed and trimmed AE tables. In total, 55 and 48 non-AE MedDRA terms were removed, respectively, based on ontology-guided exclusion criteria.

More specifically, Pfizer-BioNTech monovalent vaccine showed a reduction in significant AEs (64 in 2020–2021 vs. 48 in 2020–2024), and Moderna monovalent vaccine also showed fewer AEs (23 vs. 14) (Figure 2, Supplementary Materials S1–S3). Across all three vaccines, *taste disorder* was the only AE shared by all during 2020–2024, whereas *ageusia* and *taste disorder* were shared in 2020–2021. These findings suggest a reduction in the number of statistically significant AE signals meeting our predefined criteria in 2020–2024 relative to 2020–2021, within the limitations of passive reporting. Notably, the Janssen vaccine retained a relatively higher number of unique AEs across both periods, with 38 unique AEs in 2020–2024 and 32 unique AEs in 2020–2021.

### 3.3 AE profile comparisons between classic monovalent and new bivalent versions of the Pfizer-BioNTech and Moderna vaccines

**FIGURE 3:**
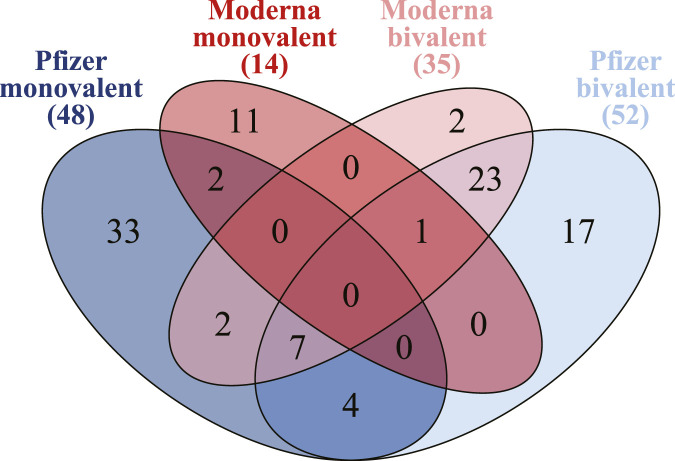
Overlap analysis of statistically significant adverse events (AEs) for monovalent and bivalent formulations of Pfizer and Moderna COVID-19 vaccines. This Venn diagram integrates the results for both manufacturers to highlight shared and unique AE profiles across their monovalent and bivalent versions (trimmed versions).

To assess the effects of the newer bivalent formulations on safety, we compared the AE profiles of monovalent and bivalent Pfizer-BioNTech and Moderna vaccines (Figure 3). Novavax was excluded from these analyses because of its small number of AE reports, as noted in Section 3.1. For Pfizer-BioNTech, the bivalent vaccine was associated with 52 statistically significant AEs, slightly higher than the 48 AEs observed for the monovalent formulation. Eleven AEs were shared between both formulations, with the top three (based on the highest average PRR values) being *acute respiratory failure*, *acute myocardial infarction*, and *hypoxia*. The bivalent formulation also had 41 unique AEs, with the top three (based on the highest PRR values) being *obstructive sleep apnoea syndrome*, *paranasal sinus hypersecretion*, and *chronic respiratory failure*. The monovalent formulation showed 37 unique associations, with the top three being *heavy menstrual bleeding*, *menstrual disorder*, and *dysmenorrhoea*.

In contrast, Moderna showed a larger difference in the number of AEs. The bivalent formulation was associated with 35 AEs, nearly three times as many as the 14 AEs for the monovalent formulation. Only one AE, *immunization reaction*, was shared between the two formulations. The bivalent formulation was uniquely associated with 34 AEs, with the top three including *brain fog*, *paranasal sinus hypersecretion*, and *upper-airway cough syndrome*. The monovalent formulation had unique associations with 13 AEs, most of which were related to skin and the top three being *vaccination site pruritus*, *vaccination site rash*, and *vaccination site induration*.

These results demonstrate that while the core AE profiles of monovalent and bivalent formulations share similarities, the bivalent formulations introduced notable differences, potentially reflecting changes in vaccine composition, population exposure, or reporting behaviors. Detailed data for these comparisons are available in Supplementary Material S4.

### 3.4 AE profiles associated with five COVID-19 vaccines

**FIGURE 4:**
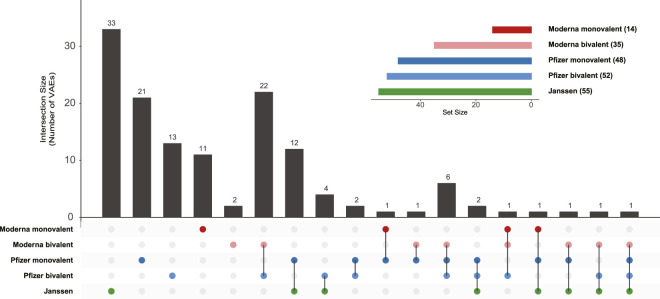
Overlap among five statistically significant VAE sets. VennDetail was used to perform the overlap analysis and visualization of the results as an UpSet plot. The upset plot function was modified to enhance the visual representation of the plot (script available in our GitHub repository).

The overlap analysis across the five COVID-19 vaccines (Pfizer-BioNTech monovalent, Moderna monovalent, Janssen, and the bivalent versions of Pfizer-BioNTech and Moderna) revealed notable patterns in AEs that were shared or unique to each vaccine (Figure 4, Supplementary Material S5). Janssen had the largest set of 33 unique AEs associated with only this vaccine, highlighting its distinctive profile as a recombinant viral vector vaccine. In contrast, the Pfizer-BioNTech and Moderna vaccines, both monovalent and bivalent, exhibited substantial overlap in AEs, reflecting their shared mRNA platform and similar mechanisms of action.

Interestingly, the intersection of the AEs shared across all five vaccines was minimal, with no AE common across all five. This finding highlights the distinct safety profiles of each vaccine type and formulation, likely reflecting the differences in platform technology, formulation, and target populations. The circular Venn diagram (Supplementary Figure S1) complements this analysis by visually representing these intersections and further highlighting the unique contribution of Janssen to the diversity of AE profiles. Detailed counts and data tables are available in Supplementary Material S5. The limited overlap also underscores the need for ongoing, platform-specific monitoring to ensure the timely detection of emerging safety signals and reinforce the importance of tracking vaccine-specific AE profiles to optimize vaccine safety and public confidence.

### 3.5 Three selected concerning AEs revisited: myocarditis, Guillain-Barré syndrome (GBS), and thrombosis

We next analyzed three clinically important AEs, including myocarditis, GBS, and thrombosis, for the two bivalent vaccines (Table 2). As introduced in the Introduction section and discussed further in the Discussion section, these three AEs are clinically significant, warned about by national authorities including CDC and FDA (MacNeil et al., 2021; Oliver et al., 2022; CDC, 2025; Pan et al., 2025), and were systematically examined in our earlier study (Guo et al., 2022). In this updated analysis, we also evaluated the effect of sex on these three AEs.

**TABLE 2:** Statistical analysis of three selected concerning VAEs during 2020–2024.

AE	​	Pfizer monovalent	Pfizer bivalent	Moderna monovalent	Moderna bivalent	Janssen
GBS	Cases (%, PRR)	501 (0.11%, 0.32)	25 (0.11%, 0.36)	356 (0.08%, 0.23)	10 (0.06%, 0.20)	296 (0.4%, 1.39)
	Cases females (%)	242 (0.08%)	15 (0.11%)	186 (0.06%)	4 (0.04%)	119 (0.29%)
	Cases males (%)	231 (0.16%)	10 (0.11%)	162 (0.13%)	6 (0.10%)	146 (0.54%)
	F/M case ratio	1.05	1.50	1.15	0.67	0.82
	F/M % ratio	**0.52**	1.02	**0.52**	**0.44**	**0.54**
Myocarditis	Cases (%, PRR)	**2,078 (0.46%, 3.55)**	26 (0.11%, 0.52)	1,086 (0.25%, 1.26)	19 (0.11%, 0.52)	115 (0.16%, 0.73)
	Cases females (%)	514 (0.18%)	11 (0.08%)	317 (0.11%)	9 (0.09%)	33 (0.08%)
	Cases males (%)	1,498 (1.02%)	15 (0.16%)	728 (0.57%)	10 (0.16%)	65 (0.24%)
	F/M case ratio	0.34	0.73	0.44	0.90	0.51
	F/M % ratio	**0.17**	**0.50**	**0.20**	**0.59**	**0.34**
Thrombosis	Cases (%, PRR)	**2,367 (0.52%, 2.12)**	36 (0.15%, 0.48)	1,630 (0.37%, 1.26)	41 (0.24%, 0.75)	**1,311 (1.77%, 6.98)**
	Cases females (%)	1,451 (0.50%)	20 (0.14%)	984 (0.34%)	27 (0.28%)	678 (1.65%)
	Cases males (%)	790 (0.54%)	15 (0.16%)	594 (0.46%)	14 (0.22%)	488 (1.79%)
	F/M case ratio	1.84	1.33	1.66	1.93	1.39
	F/M % ratio	0.92	0.91	0.74	1.27	0.92
Total AE cases		453,010	23,575	436,071	17,369	74,108

Cells with an F/M % ratio below 0.6 and those with a PRR, greater than 2 in overall cases are highlighted in bold. Because some case reports lacked gender information, the total number of cases may not equal the sum of female and male cases. PRR: proportional reporting ratio.

For the three monovalent vaccines, the findings were consistent across the 2020–2021 and 2020–2024 periods. Thrombosis remained statistically significant for both Janssen (1.77% case reports; PRR = 6.98) and Pfizer-BioNTech monovalent (0.52% case reports; PRR = 2.12), but not for Moderna monovalent (Table 2). Myocarditis was also statistically significant for the Pfizer-BioNTech monovalent (0.46%; PRR = 3.55), but not for Janssen or Moderna monovalent (Table 2). GBS, while still reported for all three vaccines, did not meet the statistical significance threshold. These results are aligned with our earlier study between 2020–2021 (Guo et al., 2022), confirming the stability of these safety signals over time. In contrast, the bivalent formulations of Pfizer-BioNTech and Moderna showed no statistically significant associations with these three selected concerning AEs, demonstrating improved safety profiles of bivalent formulations with respect to these serious events.

Sex-stratified analyses revealed notable differences. GBS and myocarditis were reported more frequently in males as indicated by the female-to-male (F/M) % ratio < 0.6 (Table 2), except for Pfizer-BioNTech bivalent (F/M % ratio = 1.02). For thrombosis, however, sex distribution was more balanced, with F/M % ratios ranging from 0.74 to 1.27 across vaccines. These findings suggest that sex differences in AE risk are event-specific, with males being more vulnerable to myocarditis and GBS, while thrombosis is relatively evenly distributed.

### 3.6 Differential sex effects on statistically significant and nonsignificant vaccine AEs

Building on our 2020–2021 analysis (Guo et al., 2022), which found that females were consistently associated with higher reporting of the top 10 ranked AEs, we re-evaluated sex-specific patterns using the expanded 2020–2024 dataset. We first confirmed that females were more likely than males to report common AEs, including *headache*, *fatigue*, and *pyrexia*. Among the 16 most frequently reported AEs, representing the top 10 AEs ranked by the number of case reports, 14 showed F/M ratios that were >1 (Table 3), ranging from 1.22 for *pyrexia* to 1.59 for *headache*. The only exceptions were *dyspnoea* and *asthenia*, with F/M ratios of 0.93 and 0.96, respectively. In total, female cases accounted for 624,161 reports, more than double the 304,975 reports from males, consistent with the overall pattern of higher reporting among females.

**TABLE 3:** Sex effect on the top-ranked AEs based on the VAERS case reports for all COVID-19 vaccines. A total of 16 AEs, collectively including the top 10 female-specific AEs and the top 10 male-specific AEs, are listed.

#	AE	# Of cases in females	% In total female cases	# Of cases in males	% In total male cases	F/M case ratio	F/M % ratio
1	Headache	107,287	17.19%	33,016	10.83%	3.250	1.588
2	Fatigue	91,037	14.59%	31,564	10.35%	2.884	1.409
3	Pyrexia	86,889	13.92%	34,896	11.44%	2.490	1.217
4	Pain	79,472	12.73%	24,972	8.19%	3.182	1.555
5	Chills	73,337	11.75%	26,080	8.55%	2.812	1.374
6	Dizziness	57,457	9.21%	22,410	7.35%	2.564	1.253
7	Pain in extremity	60,819	9.74%	18,169	5.96%	3.347	1.636
8	Nausea	62,065	9.94%	16,669	5.47%	3.723	1.819
9	Dysponea	34,590	5.54%	18,180	5.96%	1.903	0.930
10	Myalgia	34,847	5.58%	13,728	4.50%	2.538	1.240
11	Arthralgia	35,197	5.64%	13,564	4.45%	2.595	1.268
12	Injection site pain	34,071	5.46%	9,153	3.00%	3.722	1.819
13	Rash	32,699	5.24%	9,888	3.24%	3.307	1.616
14	Pruritus	32,306	5.18%	6,348	2.08%	5.089	2.487
15	Asthenia	24,148	3.87%	12,319	4.04%	1.960	0.958
16	Vomiting	24,684	3.95%	8,730	2.86%	2.827	1.382

Total cases reported females: 624,161; Total cases reported males: 304,975.

However, a closer look at statistically significant AEs revealed a more complex picture. Using thresholds of a two-fold difference in F/M % ratios and at least 200 case reports, we identified 21 AEs (19 unique OAE concepts) that were biased towards females and 21 AEs (20 unique OAE concepts) that were biased towards males (Supplementary Material S6). Among the female-biased AEs, the top three were *injection site pruritus* (F/M % ratio 6.52), *vaccination site warmth* (F/M % ratio = 5.68), and *vaccination site erythema* (F/M % ratio 4.42). Among the male-biased AEs, the top three were *myocarditis* (F/M % ratio = 5.29), *troponin level increased* (F/M % ratio 4.05), and *acute myocardial infarction* (F/M % ratio = 3.07).

**FIGURE 5:**
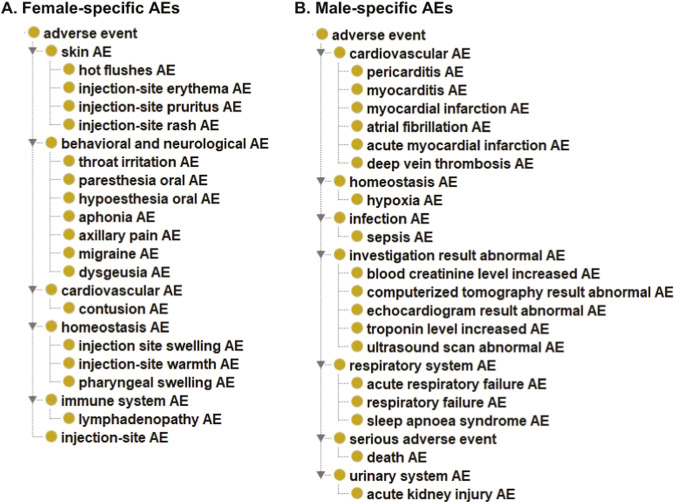
Ontological representation of female- and male-biased COVID-19 vaccine AEs. AEs predominantly biased in females **(A)** and males **(B)** among statistically significant AEs were included in this representation. Female-biased AEs were defined as those with an F/M % ratio ≥ 2.0 and at least 200 case reports, while male-biased AEs were defined as those with an F/M % ratio ≤ 0.5 and at least 200 case reports. The AEs at the bottom of the hierarchy are the significantly identified AEs from our analysis. The intermediate layer AEs are included for AE classification. Various injection-site AEs are available and classified under specific groups instead of ‘injection-site AE’ for the display simplification. Ontology of Adverse Events (OAE) was used as the basis for the AE classification of the current VAERS 2020–2024 dataset.

To further categorize these female- or male-biased AEs, we applied an ontology-based hierarchical classification using the Ontology of Adverse Events (OAE) (He et al., 2014) (Figure 5). Female-biased AEs (Figure 5A) were generally non-life-threatening and clustered in categories such as sensory disturbance, local injection-site reactions, or homeostasis-related responses. Examples include *rash*, *erythema*, and *pruritus* (injection-site AEs); *oral paresthesia*, *aphonia*, and *throat irritation* (sensory AEs); and *injection site swelling*, *pharyngeal swelling*, and *injection site warmth* (homeostasis AEs).

In contrast, male-biased AEs were often more severe and clinically significant, being related to cardiovascular, respiratory, or abnormal laboratory/test result categories (Figure 5B). Cardiovascular AEs included *myocarditis*, *pericarditis*, and *acute myocardial infarction* (heart attack). Respiratory AEs included *respiratory failure* and *sleep apnoea syndrome*. Test abnormal AEs included *blood creatinine level increase*, *CT scan abnormal*, and *echocardiogram result abnormal*. Many of these test-based findings were directly or indirectly linked to cardiovascular or respiratory conditions. For example, *echocardiogram result abnormal* can indicate uneven heartbeats or heart structure abnormalities (Ramalho et al., 2021); *troponin level increase* is related to heart muscle damage and acute myocardial infarction (Chauin, 2021); and *blood creatinine level increase* often reflects renal dysfunction (Chertow et al., 2005).

**FIGURE 6:**
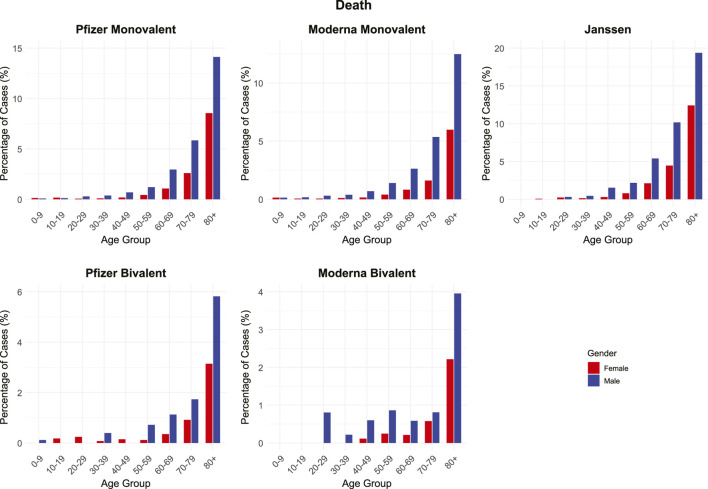
Sex- and age-specific distribution of death-related adverse events (AEs) following COVID-19 vaccination (VAERS, 2020–2024). Percentages of reported death AEs are shown by age group and sex for Pfizer (monovalent and bivalent), Moderna (monovalent and bivalent), and Janssen vaccines.

Particular attention was given to the “*death*” AE, which showed a bias towards males (Figure 5B). The *death* AE had an F/M % ratio of 0.352, equivalent to a male-to-female (M/F) % ratio of 2.84, the fifth highest among all the examined AEs. Further stratification by age revealed that *death* cases were more frequent in males, especially among older age groups. The reporting percentage increased substantially with advancing age across all vaccine types, with the highest percentages observed in individuals aged 80 and older (Figure 6). When comparing formulations, monovalent vaccines (both Pfizer-BioNTech and Moderna) were associated with notably higher death-reporting percentages than their bivalent counterparts, approximately two to three times higher in some age groups. These findings highlight the interplay among age, sex, and vaccine formulation in this rare but serious AE.

### 3.7 Age comparison between the periods of 2022–2024 and 2020–2021

**FIGURE 7:**
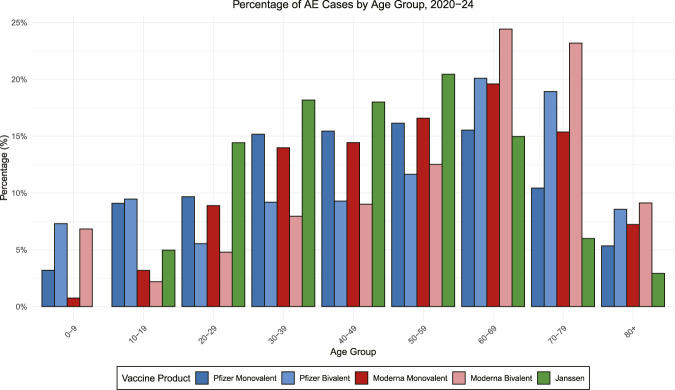
Age distribution of reported COVID-19 AEs, 2020–2024. Percentages of reported AEs are shown by age group for Pfizer (monovalent and bivalent), Moderna (monovalent and bivalent), and Janssen vaccines. Percentages are relative to the total AE reports for each vaccine formulation.

Figure 7 illustrates the percentages of AEs across different age groups for the five COVID-19 vaccines from 2020 to 2024. To avoid unstable percentage estimates, vaccine-by-subgroup strata with fewer than 100 total reports were excluded from stratified analyses, resulting in the exclusion of the 0–9 age group for the Janssen vaccine. Age-dependent patterns were evident across all vaccine types, though their magnitude varied by formulation.

Overall, the percentages of AEs generally increased with age, with peaks observed in later age groups (most commonly 50–69, depending on the vaccine and AE), before tapering off in older groups (70–79 and 80+) (Figure 7). This trend was most pronounced for Pfizer-BioNTech and Moderna monovalent vaccines and their bivalent versions, which consistently showed higher AE percentages in middle-aged adults than in younger and older groups. The bivalent formulations further showed elevated percentages in the 60–69 and 70–79 age groups compared to their monovalent counterparts, suggesting potential differences in formulation or immunogenicity that influence age-specific responses. Deviated from this overall pattern described above, the Janssen vaccine showed a distinct age distribution of age groups, which might reflect differences in immune mechanisms, reporting biases, formulations, and vaccine use in specific populations.

In summary, these results in Figure 7 highlight the complexity of age-specific responses to COVID-19 vaccines, emphasizing the need for age-stratified surveillance and mechanistic research to better understand these patterns.

### 3.8 Combined age and sex effects on COVID-19 AEs

As described above, from over 40 statistically significant AEs found, we observed clear age- and sex-related trends. For the AEs that were most prevalent in males, case percentages increased with age. The monovalent Pfizer-BioNTech and Moderna formulations showed more consistent patterns across different AEs, while the bivalent formulations showed more significant variations, suggesting changes in the underlying risk profiles or population exposure. In contrast, AEs, which are more common in females, tended to lack sharp peaks but instead displayed a broader, curved distribution across the 20–70 age range. This pattern was consistent across monovalent formulations, while the bivalent formulations demonstrated more variability by AE, reflecting the evolving dynamics of vaccine use and reporting.

**FIGURE 8:**
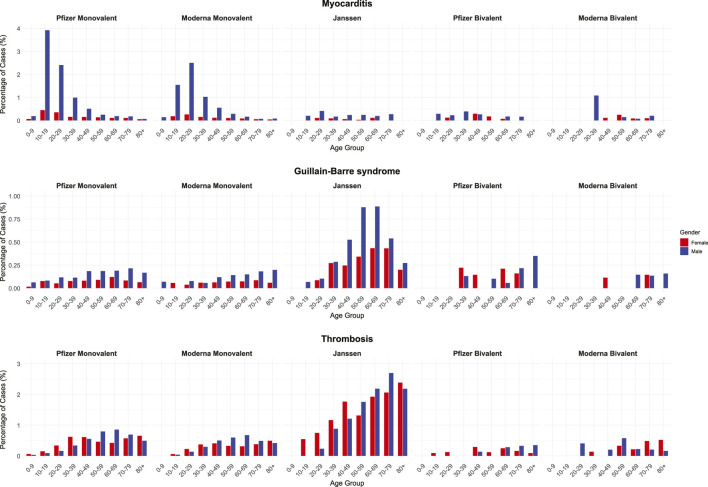
Combined age-by-sex profiles of myocarditis, Guillain-Barré syndrome, and thrombosis across COVID-19 vaccine formulations. Bar graphs depict case percentages stratified by age group (0–9 through 80+) and sex (red: female; blue: male) for monovalent and bivalent formulations of Pfizer and Moderna vaccines as well as Janssen vaccines.

Building upon these general observations, we evaluated three clinically important AEs: *myocarditis*, *GBS*, and *thrombosis* in greater detail (Figure 8). For myocarditis, monovalent formulations showed case percentages that peaked in adolescence and early adulthood, then declined with age. The Pfizer-BioNTech monovalent exhibited the highest percentages, peaking at approximately 4% in the 10–19 age group in males. This sharp early-age peak diminished with increasing age. Females, by contrast, showed consistently low myocarditis case percentages (<0.5%) across all age groups. Bivalent formulations showed substantially lower percentages for both sexes.

For GBS and thrombosis, age played a dominant role in shaping risk profiles, though the patterns differed in magnitude and sex-specificity. For GBS, male predominance was evident, with case percentages gradually increasing with age and peaking in the 50–69 age group, whereas female case percentages remained lower and more evenly distributed across age groups, indicating less age-related variability. Thrombosis showed a similar age-related trend, with the highest clustering of cases occurring in individuals aged 70 and above, regardless of sex. Although males exhibited slightly higher percentages overall, the sex differences were less pronounced than those observed for myocarditis or GBS. These findings underscore the combined effects of aging, comorbid conditions, and, in the case of thrombosis, a potentially higher baseline thrombotic risk in older populations.

Overall, the analysis of the evaluated AEs revealed distinct age-related clustering patterns (Supplementary Materials S7,S8). AEs such as *contusion*, *dysgeusia*, and *throat irritation*, as shown in *myocarditis* above, were predominantly reported in younger individuals, while *migraine*, *vaccination site pruritus*, and *pericarditis* were more frequent in middle-aged groups. In contrast, *acute myocardial infarction*, *respiratory failure*, and *deep vein thrombosis* showed a marked increase in older adults, especially males, paralleling the pattern observed for the *death* AE. These patterns indicate that certain AEs were age-specific, potentially reflecting biological susceptibility or comorbidity burden by age group.

## 4 Discussion

This study updates the safety profiles of COVID-19 vaccines using more than 3 years of VAERS data, capturing the transition from monovalent to bivalent formulations. We found fewer statistically significant AEs associated with monovalent mRNA Pfizer-BioNTech and Moderna vaccines during the period of 2020–2024 compared with 2020–2021. In contrast, the Janssen vaccine showed a slight increase in unique AEs during 2020–2024, even though its total number of reports decreased. Analysis of the three selected concerning AEs (myocarditis, GBS, and thrombosis) showed that males were more frequently affected, particularly for myocarditis with the Pfizer-BioNTech monovalent vaccine and thrombosis with the Pfizer-BioNTech monovalent and Janssen vaccines. Females were more likely to report common and non-life-threatening AEs, whereas males were associated with various significant and often severe, clinically significant AEs, especially cardiovascular events. Age, and particularly its interaction with sex, also strongly influenced the AE distribution.

While our study analyzed all statistically significant AEs associated with COVID-19 vaccines, we specifically focused on three selected concerning AEs, including myocarditis, GBS, and thrombosis, which were specifically warned about by the CDC and FDA and were also emphasized in our previous VAERS study (Guo et al., 2022). Specifically, the thrombosis AE was chosen because it was a major reason why the Janssen COVID-19 vaccine administration was halted in 2021 (MacNeil et al., 2021; Oliver et al., 2022). The FDA previously also released a warning that GBS could be a side effect of the Janssen COVID-19 vaccine (Woo et al., 2021; Guo et al., 2022; Thant et al., 2022). The FDA and other health authorities have also acknowledged and issued warnings of myocarditis as a potentially significant adverse event of the Pfizer-BioNTech and Moderna COVID-19 vaccines, especially after the second dose (Ahmed, 2022; Heidecker et al., 2022; Florek and Sokolski, 2024; FDA, 2025). Active surveillance studies conducted in defined cohorts have reported higher myocarditis detection rates than passive reporting systems such as VAERS, including frequencies of approximately 2%–3% in selected populations undergoing systematic cardiac evaluation (Mansanguan et al., 2022; Buergin et al., 2023). These differences likely reflect study design, case definitions, population characteristics, and monitoring intensity, and are not directly comparable to spontaneous reporting data used for signal detection.

Meanwhile, although only three concerning AEs were focused on in this study, there have been many more severe AEs associated with COVID-19 (Pan et al., 2025). Indeed, the CDC has collected a list of commonly found AEs for COVID-19 vaccines (CDC, 2025). Notably, using the National Clinical Cohort Collaborative (N3C), our recent studies found that COVID-19 vaccination is associated with a significantly lower acute kidney injury risk compared to COVID-19 infection (Pan et al., 2024), and among 15 studied AEs, only anaphylaxis was statistically significantly associated with XBB.1.5-containing COVID-19 vaccines (Pan et al., 2025). The current VAERS study provided a complementary aspect of the COVID-19 vaccine AE profiles.

According to our systematic analysis of the VAERS data, the AE profiles with COVID-19 vaccines, especially mRNA vaccines, appeared to improve over the period of 2020–2024 compared with 2020–2021 (Guo et al., 2022). This improvement was evident across three comparisons: between monovalent vaccines across two periods, between monovalent and bivalent vaccines, and in the reassessment of three selected concerning AEs. For the classic monovalent vaccines, the number of statistically significant AEs decreased from 113 in 2020–2021 to 98 in 2020–2024 (Figure 2). Among the shared AEs, *ageusia* and *taste disorder* were the only two shared during 2020–2021, while only *taste disorder* was shared by the three vaccines in 2020–2024. Interestingly, *ageusia* refers to the complete loss of the sense of taste. This observation reflects the reduction of taste disorder severity over time, suggesting a safer profile of these vaccines during the longer follow-up period, which aligns with previous reports (Riad et al., 2023; Li et al., 2024). The newly introduced bivalent vaccines, introduced after 2021, showed fewer statistically significant AE signals (per our predefined criteria) (Figure 3) and no significant associations with the three selected concerning AEs (thrombosis, GBS, and myocarditis) (Table 2). While these AEs remained statistically significant for the classic monovalent vaccines across both periods, none were significantly associated with the new bivalent formulations.

Several factors might explain the reduced number of statistically significant AE signals observed during the longer period, 2020–2024. First, repeated exposures to the virus or vaccine likely led to population-level immune adaptation (Atmar et al., 2022), reducing the frequency of adverse reactions to these vaccines. Second, the shift in dominant SARS-CoV-2 strains to less virulent variants (Nyberg et al., 2022), as well as the components (e.g., the spike protein sequence variation) used in the development of the new vaccine, may have contributed. Third, improvements in vaccine manufacturing methods and the predominant use of mRNA vaccines, which have been associated with fewer serious AE signals compared with the recombinant viral-vector vaccines such as Janssen, may also have played a role (Schultz et al., 2021; Choi et al., 2024; King, 2024).

The COVID-19 mRNA vaccines are produced by *in vitro* transcription (IVT) from plasmid DNA templates (Whitley et al., 2022; Shankar et al., 2024). As a result, residual plasmid DNA fragments are an expected manufacturing byproduct and are regulated as an impurity. Regulatory guidance for biologic products has historically referenced precautionary limits for residual DNA, commonly cited as up to 10 ng per dose, with risk assessments primarily addressing theoretical infectivity and oncogenic potential (World Health, 2007; Yang, 2013; Banoun, 2023). Cytosolic double-stranded DNA can activate innate immune sensing pathways such as cGAS-STING under certain experimental conditions (Chen et al., 2016; Hopfner and Hornung, 2020). However, whether trace levels of residual plasmid DNA in mRNA vaccines are present in sufficient quantity, form, or cellular context to engage these pathways *in vivo* remains unproven. Recent publications have reported variable measurements of residual DNA in COVID-19 mRNA vaccine vials (Konig and Kirchner, 2024; Speicher et al., 2025; McKernan et al., 2026). However, the appropriate analytical methods and interpretation of these measurements have been debated in the literature (Kaiser et al., 2025; Methods and Protocols Editorial, 2025). At present, direct evidence linking residual DNA measurements to clinically meaningful adverse event outcomes remains limited. Because our study is based on passive surveillance data, it cannot evaluate product composition or establish mechanistic causality.

However, our finding of the apparently improved safety profile of more recent COVID-19 vaccines, as identified from this study, should be interpreted with caution. This study utilized data from VAERS, a passive voluntary reporting system, which has inherent limitations (Zhou et al., 2003; Varricchio et al., 2004; Shimabukuro et al., 2015; Donzelli, 2023). Due to its non-mandatory nature, the vaccine adverse events collected in VAERS were underreported, lacked follow-up investigation, and were selectively reported, with severe or media-highlighted cases being more likely to be reported. VAERS lacks the total vaccine doses administered as the true denominator. The VAERS data analysis largely relies on the Proportional Reporting Ratio (PRR), which identifies signals of disproportionate reporting instead of absolute incidence rates. In addition, VAERS data are subject to variability in report quality and coding. The raw VAERS data was manually coded by MedDRA codes, and the quality of MedDRA coding of adverse events depends on the reporters’ clarity and the coders’ interpretation. Furthermore, our observed decline in unique AEs might be partly due to the significantly reduced number of vaccine doses administered in later years, instead of enhanced safety. The “healthy vaccinee effect” (i.e., healthier individuals being more likely to receive vaccines) might further bias comparisons by potentially masking risks observed in frailer or severely ill populations (Chemaitelly et al., 2025; Riedmann et al., 2025). Further studies with higher-quality data would be required to obtain more solid evidence. Consequently, VAERS is best interpreted as a signal detection tool rather than a source of definitive incidence data. Active surveillance studies have demonstrated substantially higher rates of certain events, such as myocarditis, than passive reporting systems suggest (Donzelli, 2023), highlighting the need to contextualize VAERS findings within complementary safety data sources.

Our study also provided new insights into sex differences, expanding on our previous findings (Guo et al., 2022). In the earlier study, the top ten most frequently reported AEs were all female-biased, but they were not statistically significant. The present study incorporated biological sex into the analysis of the three selected concerning AEs and showed that, although females continue to report more AEs, males experienced more statistically significant and severe AEs, particularly those involving the cardiovascular system (Figure 5). Such a phenomenon has also been observed in other reports (Xiong et al., 2021; Al-Qazaz et al., 2022). For example, an analysis of VAERS data during the first 2 months of the U.S. vaccine rollout found that cardiopulmonary adverse effects were reported more frequently in females, while males were more often associated with serious outcomes such as hospitalization or death (Xiong et al., 2021). However, these studies were limited by their short observation periods and smaller sample sizes. In contrast, our study used more than 3 years of VAERS data, offering a broader and more statistically robust view of age- and sex-specific AE patterns, and further emphasizing the importance of stratified monitoring and risk communication.

Different mechanisms likely underlie the sex-biased vaccine immune responses and AEs. There are well-documented differences in immune responses to vaccines between males and females, influenced by genetic factors (e.g., X-linked immune-related genes) (Klein et al., 2010), hormonal factors (e.g., estrogen, progesterone, and testosterone), and differences in innate and adaptive immune mechanisms (Cook, 2008; Klein et al., 2015; Fink and Klein, 2018). As a result, females generally mount stronger immune responses to vaccines compared to males, resulting in higher antibody titers, greater vaccine efficacy, but also a higher risk of adverse reactions (Fischinger et al., 2019). Male-specific COVID-19 vaccine AEs have also been reported (Xiong et al., 2021); for example, myocarditis occurs more frequently in males, with a female-to-male ratio of 1:2–4 (Al-Qazaz et al., 2022).

An enriched male-biased pattern of *death* AE was identified in our study and was further carefully investigated. Large-scale epidemiological studies using the U.S. Vaccine Safety Datalink and VAERS found that COVID-19 vaccine recipients exhibit lower overall mortality than unvaccinated individuals or expected background rates (Xu et al., 2021; Day et al., 2023). However, these studies did not account for important statistical biases like immortal time bias (Alessandria et al., 2024) and case counting window bias (Alessandria et al., 2025), which could significantly inflate mortality rates among unvaccinated individuals. While vaccine-associated deaths have been reported in post-marketing surveillance, the causal relationships between death and vaccines are associated with many conditions such as vaccine-induced immune thrombotic thrombocytopenia (VITT) (Danish et al., 2022), myocarditis (Kim et al., 2021; Hulscher et al., 2025), severe allergic reactions including anaphylaxis (Paul et al., 2022; Jaggers and Wolfson, 2023), acute disseminated encephalomyelitis (Nabizadeh et al., 2023), myocardial infarction (Baqi et al., 2022), and rhabdomyolysis (Maiese et al., 2022). Our findings support that, although rare, males were more susceptible to the *death* AE compared to females (Supplementary Material S6). Furthermore, age, vaccine type, and vaccine formulation also influence the *death* AE outcome (Figure 6). Our results show that the *death* AE was typically seen in senior male adults, especially in the 80+ age group. The recombinant viral vector Janssen vaccine was associated with higher percentages of *death* AE among reported cases, and monovalent vaccines had notably higher death-reporting percentages than their bivalent counterparts. These findings underscore the importance of closely monitoring older adults, particularly senior males with comorbidities, while reaffirming that vaccination remains associated with lower overall mortality.

Age also had a major influence on AE occurrence, especially when it was co-studied with the sex effect. We observed that the percentages of AEs generally increased with age, with peaks occurring in middle-aged and older adults, while patterns in younger age groups varied by vaccine and were limited by subgroup size. The lower percentage in older groups (70–79 and 80+) likely reflects smaller population sizes and underreporting. When sex and age are considered together, specific patterns emerged, as demonstrated by the high *death* AE in older males. These findings are consistent with and extend previously reported age- and sex-specific biological mechanisms related to immune senescence and inflammation and warrant further mechanistic studies to elucidate underlying pathways (Jensen et al., 2022).

The benefits and risks of COVID-19 vaccines and the underlying mechanisms deserve deeper investigation. It has been strongly supported with real-world evidence that COVID-19 vaccines have substantially reduced mortality and saved millions of lives globally (Watson et al., 2022; Zheng et al., 2022; Ioannidis et al., 2025), and the vaccines targeting earlier SARS-CoV strains might induce cross-reactive responses against later virus strains (Lipsitch et al., 2020; Fenton and Lee, 2022; Moss, 2022). However, the most commonly used COVID-19 mRNA vaccines have shown reduced effectiveness after the second dose or booster dose (Chemaitelly et al., 2022; Chemaitelly et al., 2023). The effectiveness against Omicron BA.1 and BA.2 subvariants has also been reported to decline substantially over time (Chemaitelly et al., 2022; Chemaitelly et al., 2023). In certain observational analyses, long-term effectiveness estimates have declined during later follow-up, in some subgroups, have reached point estimates below zero with wide confidence intervals (Chemaitelly et al., 2023). For example, in a healthcare employee cohort during the Omicron period, Shrestha et al. reported that a higher number of prior vaccine doses was associated with a higher observed risk of subsequent COVID-19 infection, and the bivalent effectiveness estimate had a confidence interval that included negative values, consistent with limitations inherent to the observational design (Shrestha et al., 2023). Given the occurrence of various adverse events associated with COVID-19 vaccines, it is critical to identify the underlying mechanisms, which would include how different variables (e.g., sex, age, genetic variations, and comorbidities) affect the occurrences of adverse events (Wang et al., 2022). Such mechanistic understanding enables precision vaccinology approaches (Xie et al., 2021; Lagousi et al., 2022; Oh et al., 2022; Scully et al., 2025) for the development and usage of safe and effective vaccines for everyone.

Overall, this study provides an important update to the safety profile of various COVID-19 vaccines based on long-term VAERS data. Our findings underscore the importance of continuous safety surveillance and stratified analyses by sex and age to inform risk mitigation and communication strategies. Future work should integrate active surveillance systems and mechanistic studies (Ryan et al., 2023) to better estimate true incidence, account for confounders such as prior infection, and understand the biological bases of observed sex- and age-specific patterns. Expanding integration with EHR-based resources, such as N3C (Pan et al., 2024; Pan et al., 2025), All of Us (Ramirez et al., 2022), and UK Biobank (Bycroft et al., 2018), will further enhance these efforts. Furthermore, large language models (Wang et al., 2025) can be leveraged to systematically analyze these datasets and the biomedical literature (e.g., PubMed and PubMed Central) to generate deeper insights into vaccine safety and related biological mechanisms.

## Data Availability

The original contributions presented in the study are included in the article/Supplementary Material, further inquiries can be directed to the corresponding authors. The vaccine adverse event data used were obtained from the publicly available Vaccine Adverse Event Reporting System (VAERS) via the Cov19VaxKB platform (https://violinet.org/cov19vaxkb). All compiled data and scripts used in this study are provided in the Supplementary Files or available at a GitHub repository (https://github.com/hurlab/covid19VAE).

## Supplementary material

SUPPLEMENTARY FIGURE S1 Circular Venn Diagram of five VAE sets. The border colors match each vaccine.

SUPPLEMENTARY FIGURE S2 Age and Sex Patterns of all AEs. Age- and sex-stratified AE profile of all vaccines.

SUPPLEMENTARY FILE 1 Original and trimmed list of significant AEs from the 202-2021 period.

SUPPLEMENTARY FILE 2 Original and trimmed list of significant AEs from the 202-2024 period.

SUPPLEMENTARY FILE 3 Venn analysis details of three classical formulations.

SUPPLEMENTARY FILE 4 Venn analysis details of mono- and bi-valent vaccine comparison.

SUPPLEMENTARY FILE 5 Venn analysis details of all formulations.

SUPPLEMENTARY FILE 6 Tables of female and male-based AEs.

SUPPLEMENTARY FILE 7 Age- and sex-associated AE classifications.
