# Supplementary figures and images for: Updated profiling of COVID-19 vaccine adverse events using VAERS case reports
Source: Front Pharmacol. 2026 Mar 26;17:1741967. doi: 10.3389/fphar.2026.1741967 (PMC13061691; doi:10.3389/fphar.2026.1741967)

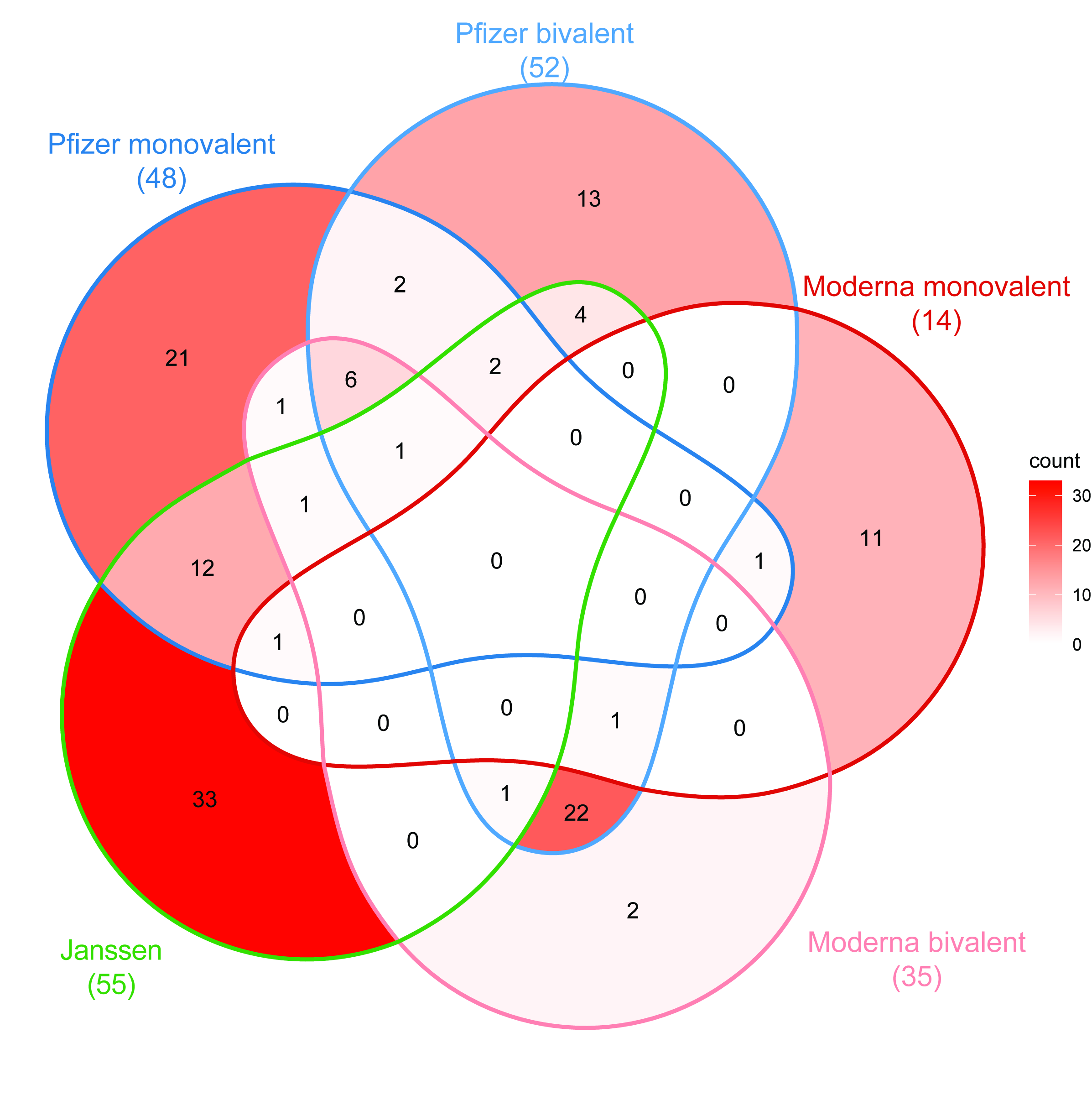

Supplement: Supplementary file 3 [file Image1.tif]
