# Supplementary material for: Updated profiling of COVID-19 vaccine adverse events using VAERS case reports
Source: Front Pharmacol. 2026 Mar 26;17:1741967. doi: 10.3389/fphar.2026.1741967 (PMC13061691; doi:10.3389/fphar.2026.1741967)
Supplement: Supplementary file 4 [file Image2.pdf]

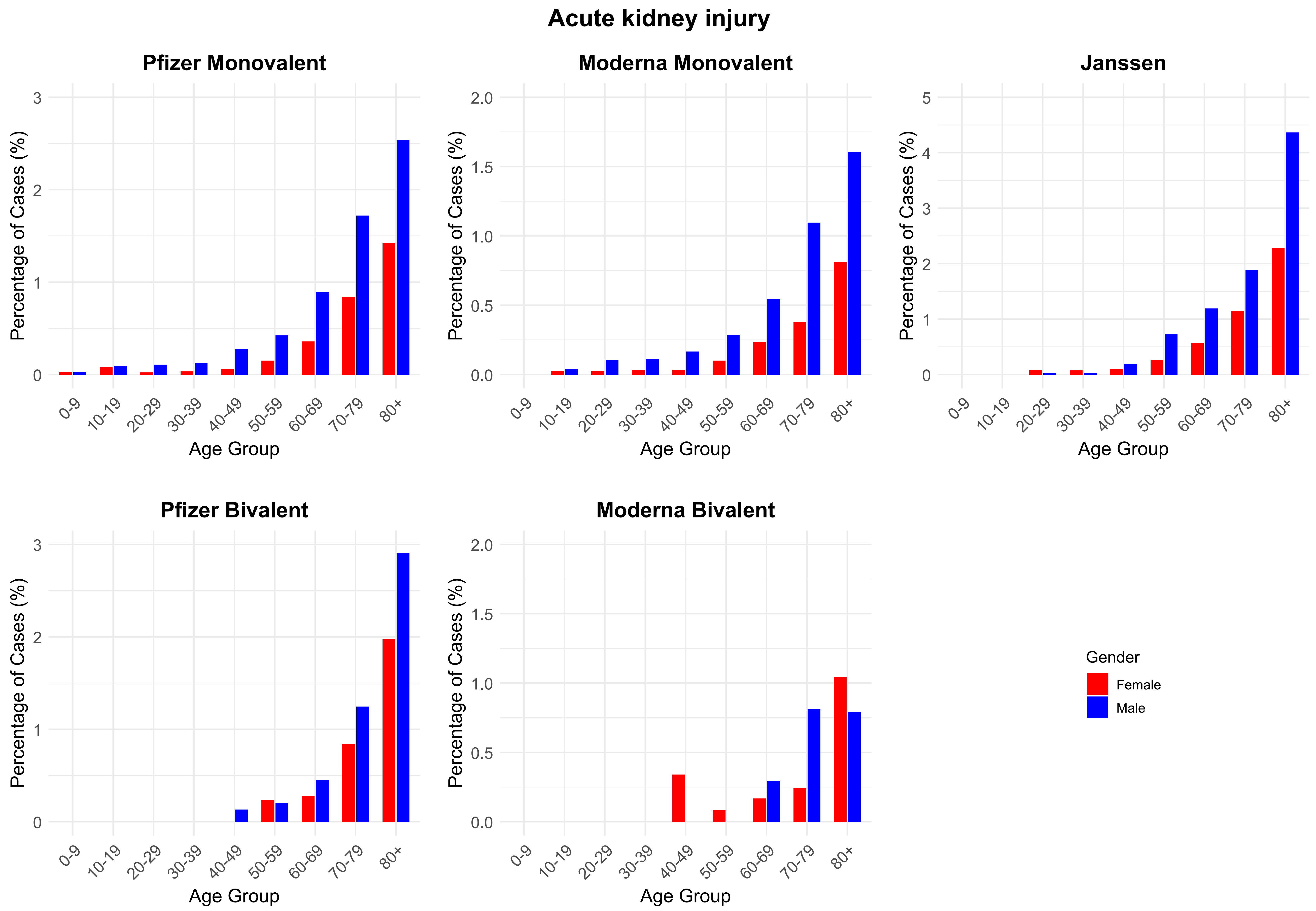
Acute kidney injury
Pfizer Monovalent
Moderna Monovalent
Janssen
Pfizer Bivalent
Moderna Bivalent
Percentage of Cases (%)
Age Group
0-9
10-19
20-29
30-39
40-49
50-59
60-69
70-79
80+
Gender
Female
Male

# Acute myocardial infarction

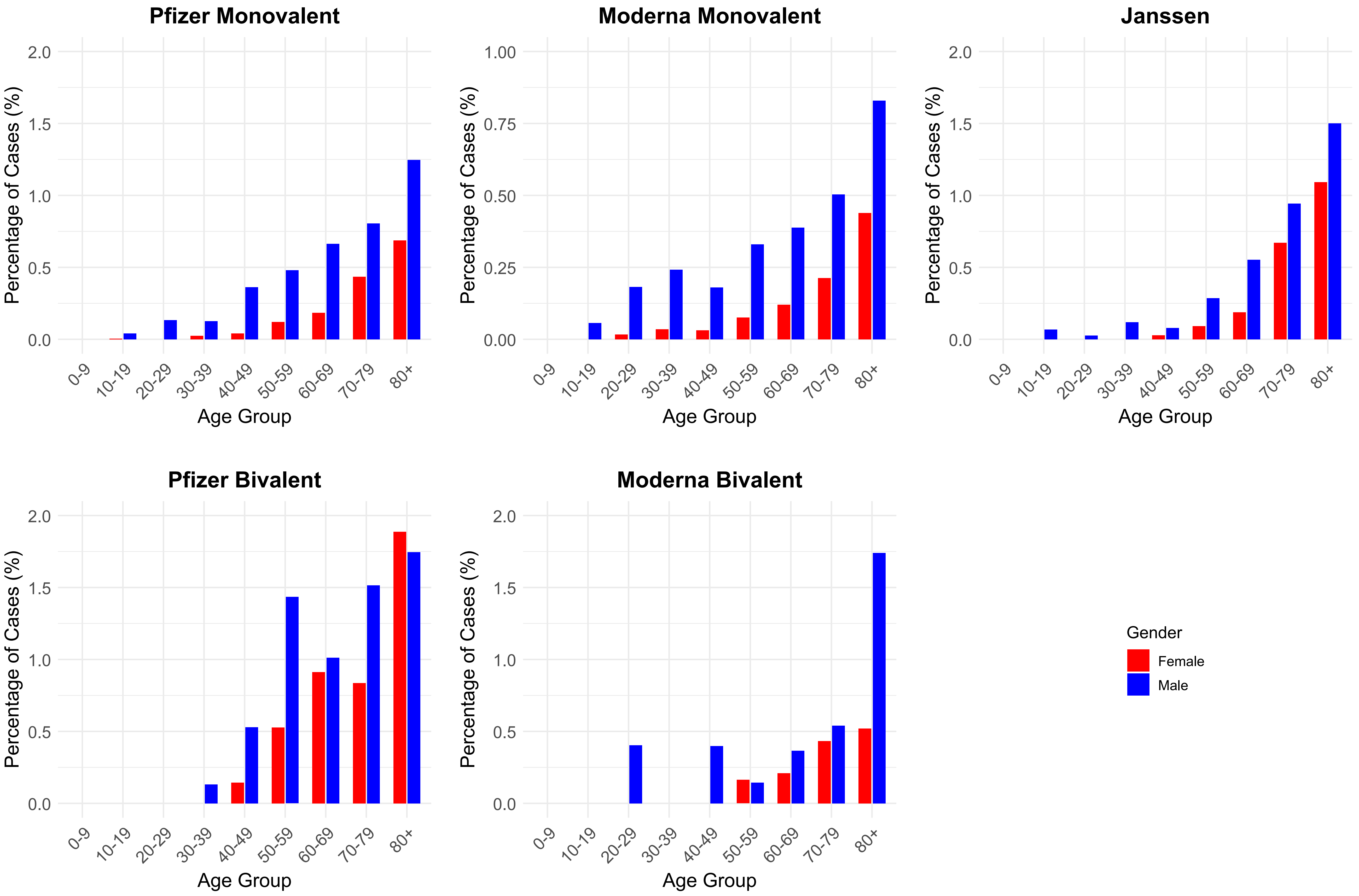

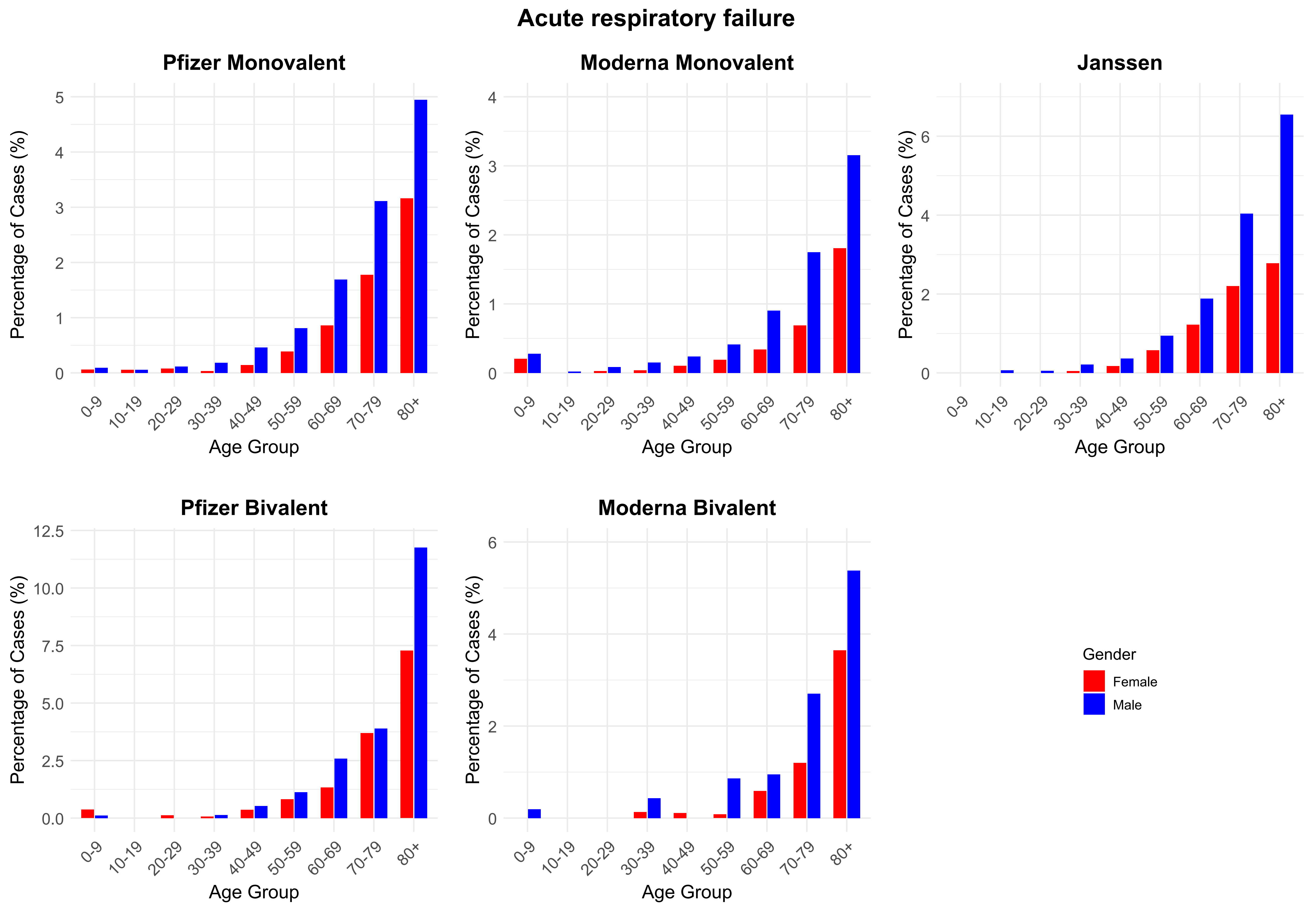

Acute respiratory failure
Pfizer Monovalent
Moderna Monovalent
Janssen
Pfizer Bivalent
Moderna Bivalent
Percentage of Cases (%)
Age Group
0-9
10-19
20-29
30-39
40-49
50-59
60-69
70-79
80+
0
1
2
3
4
5
6
0.0
2.5
5.0
7.5
10.0
12.5
Gender
Female
Male

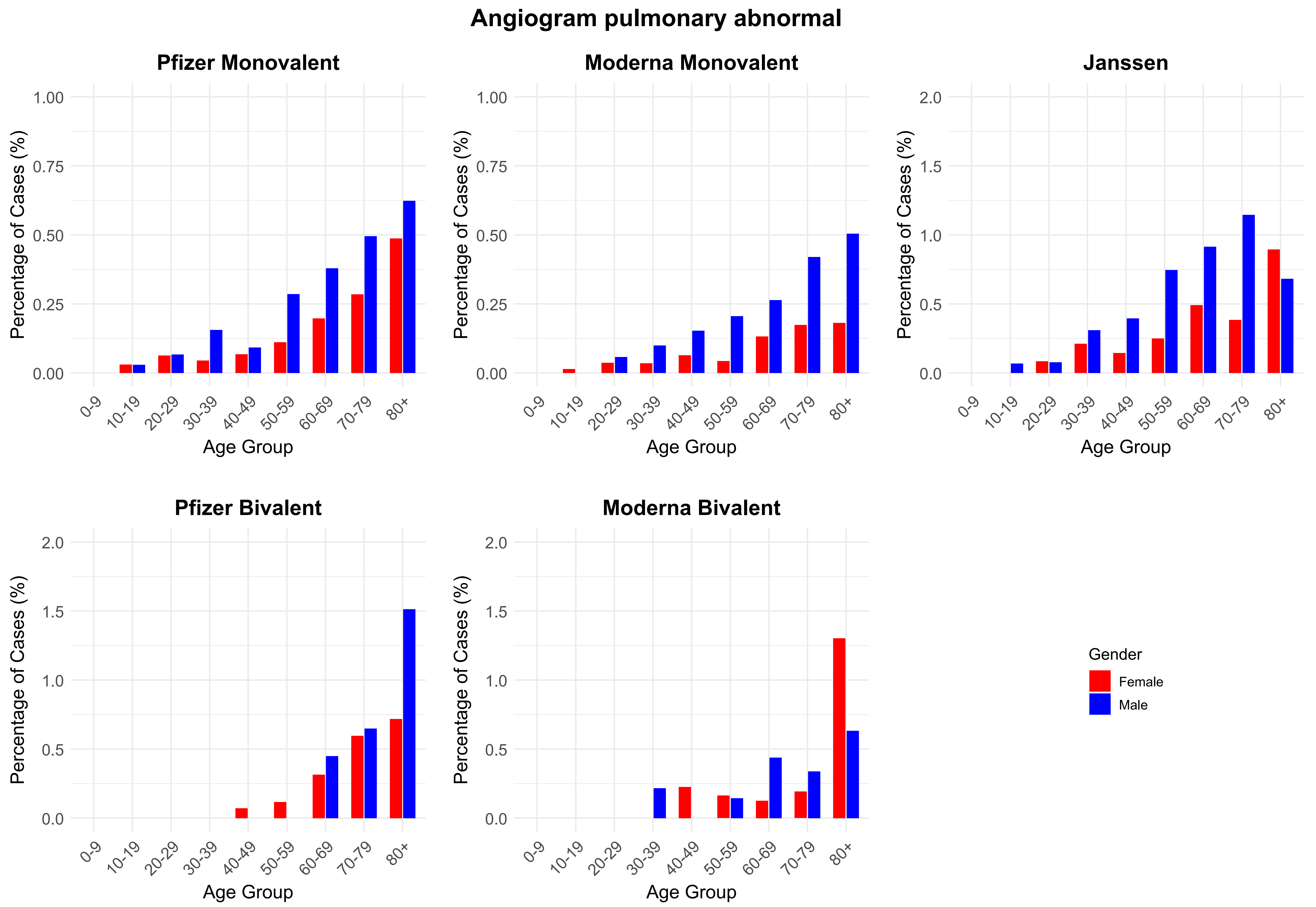

Angiogram pulmonary abnormal
Pfizer Monovalent
Moderna Monovalent
Janssen
Pfizer Bivalent
Moderna Bivalent
Percentage of Cases (%)
Age Group
0-9
10-19
20-29
30-39
40-49
50-59
60-69
70-79
80+
0.00
0.25
0.50
0.75
1.00
0.0
0.5
1.0
1.5
2.0
Gender
Female
Male

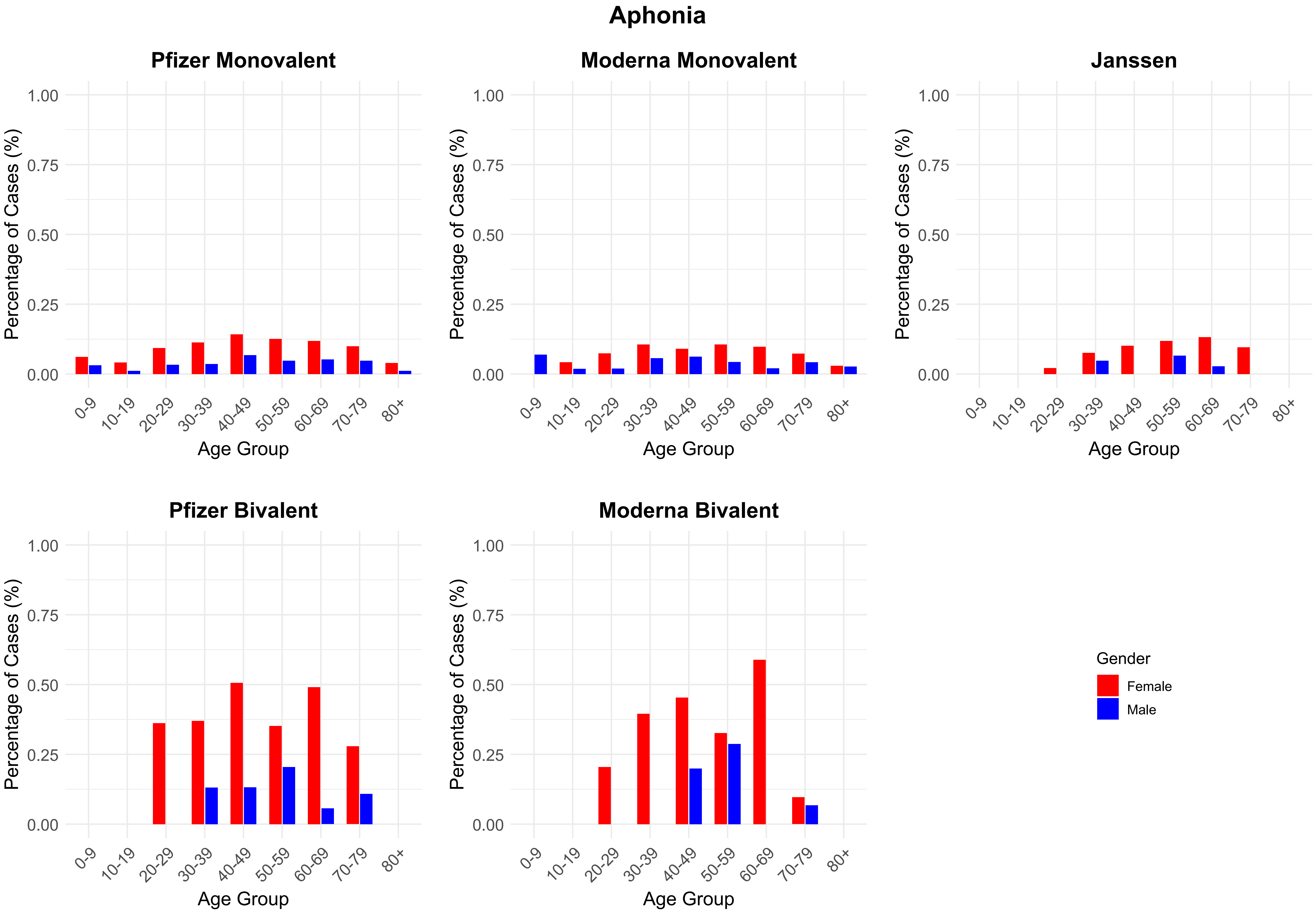
Aphonia
Pfizer Monovalent
Moderna Monovalent
Janssen
Pfizer Bivalent
Moderna Bivalent
Percentage of Cases (%)
1.00
0.75
0.50
0.25
0.00
0-9
10-19
20-29
30-39
40-49
50-59
60-69
70-79
80+
Age Group
Gender
Female
Male

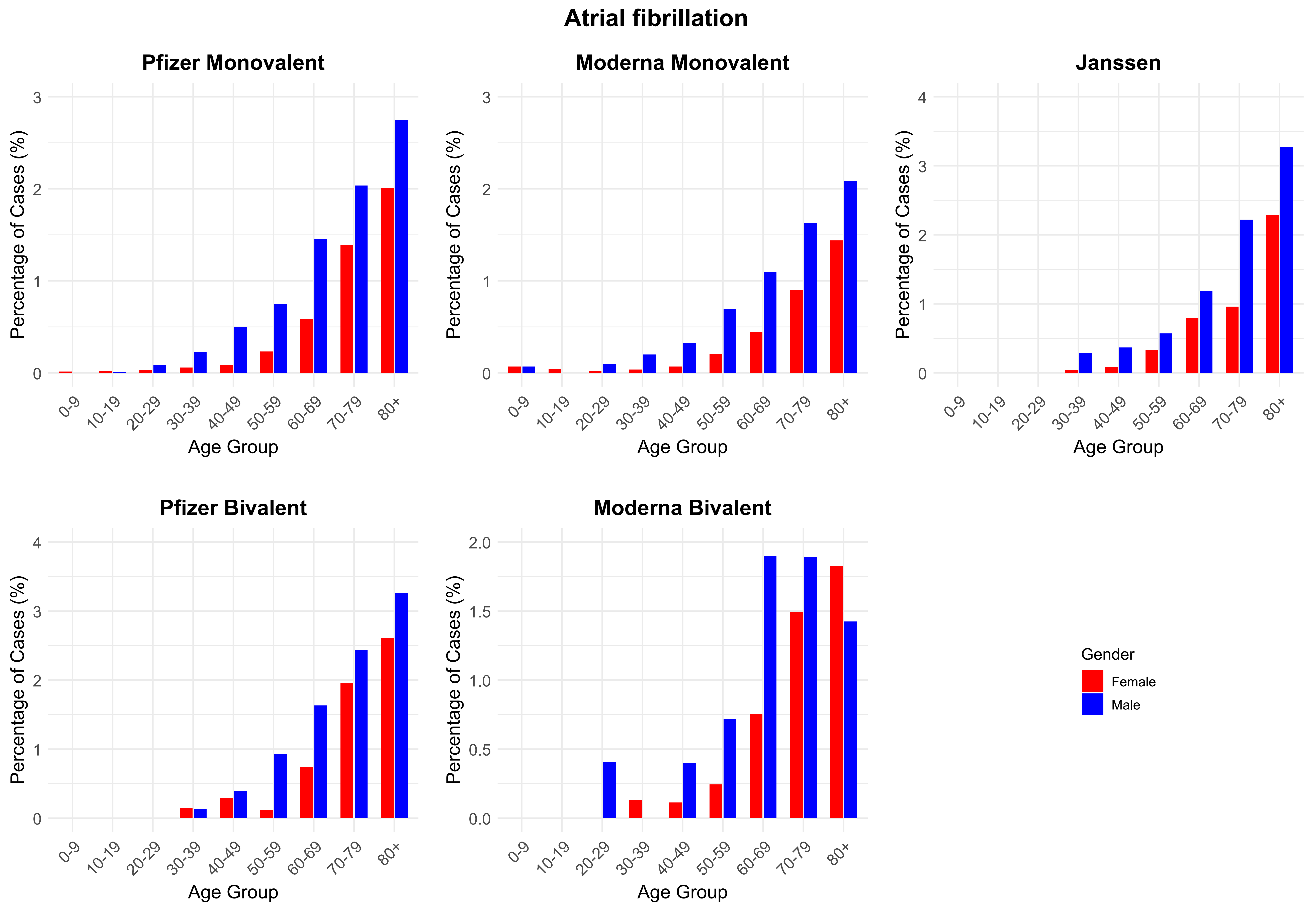
Atrial fibrillation
Pfizer Monovalent
Moderna Monovalent
Janssen
Pfizer Bivalent
Moderna Bivalent
Percentage of Cases (%)
Age Group
0-9
10-19
20-29
30-39
40-49
50-59
60-69
70-79
80+
Gender
Female
Male

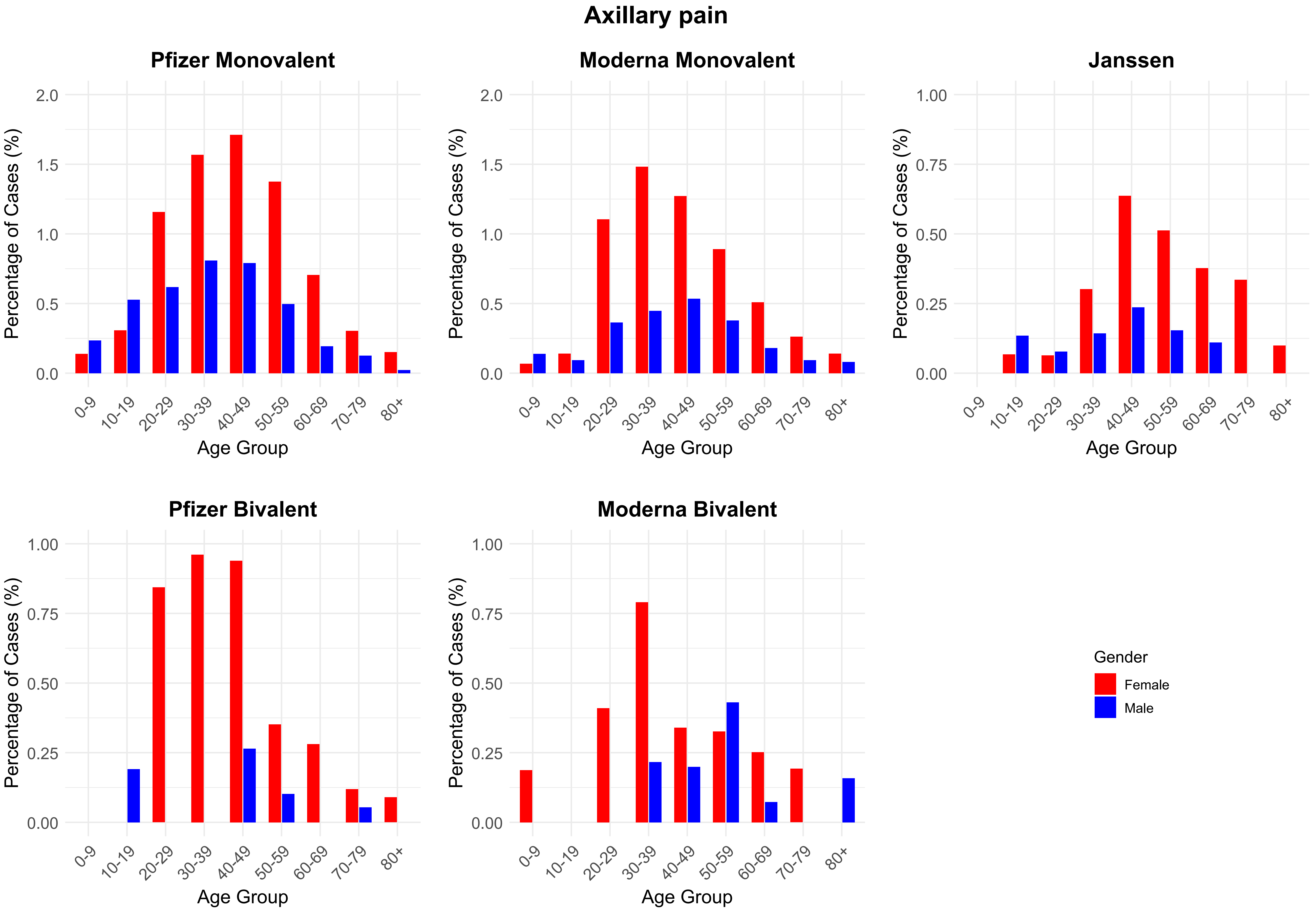
Axillary pain
Pfizer Monovalent
Moderna Monovalent
Janssen
Pfizer Bivalent
Moderna Bivalent
Percentage of Cases (%)
Age Group
0-9
10-19
20-29
30-39
40-49
50-59
60-69
70-79
80+
0.0
0.5
1.0
1.5
2.0
0.00
0.25
0.50
0.75
1.00
Gender
Female
Male

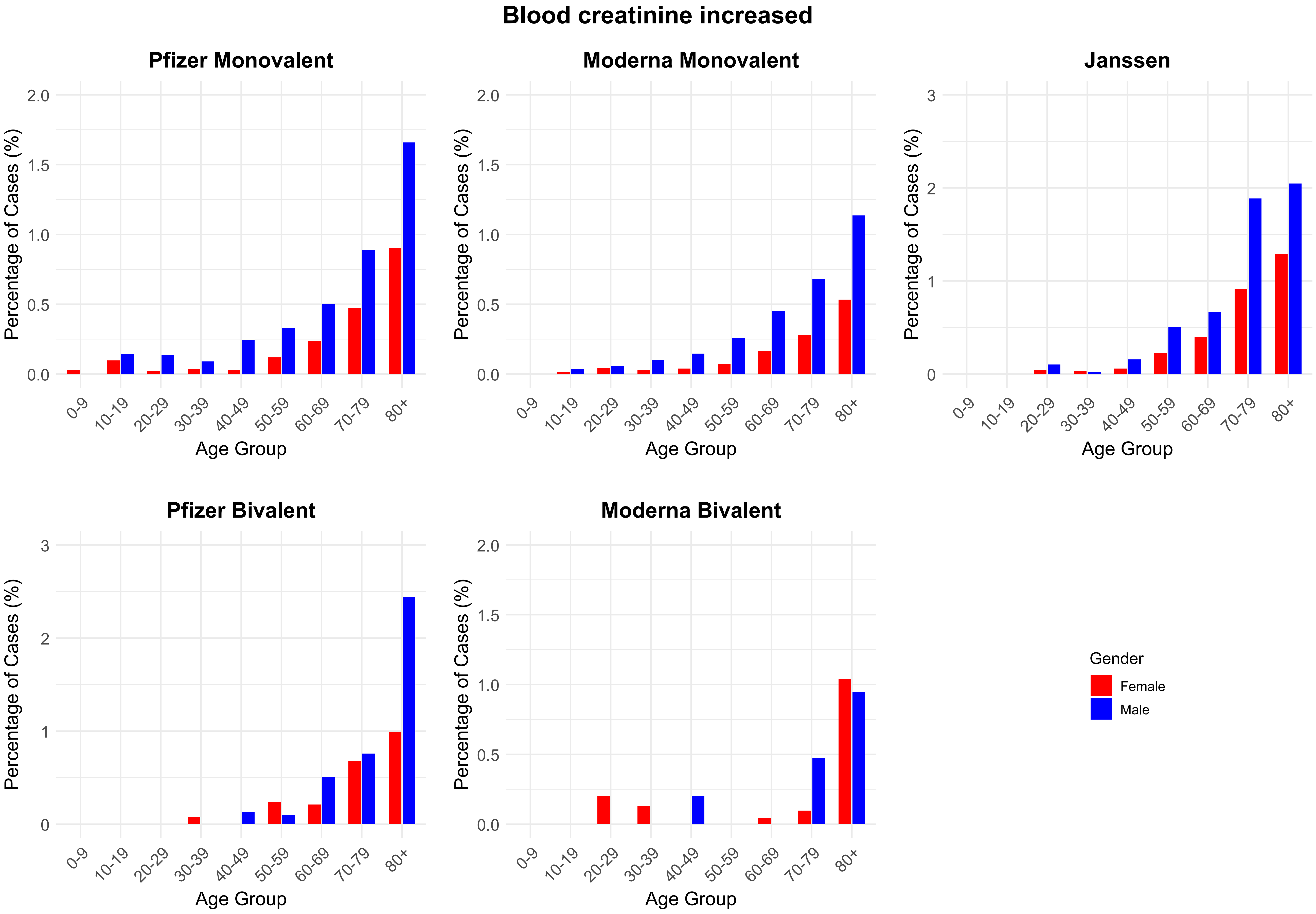
Blood creatinine increased
Pfizer Monovalent
Moderna Monovalent
Janssen
Pfizer Bivalent
Moderna Bivalent
Percentage of Cases (%)
Age Group
0-9
10-19
20-29
30-39
40-49
50-59
60-69
70-79
80+
Gender
Female
Male

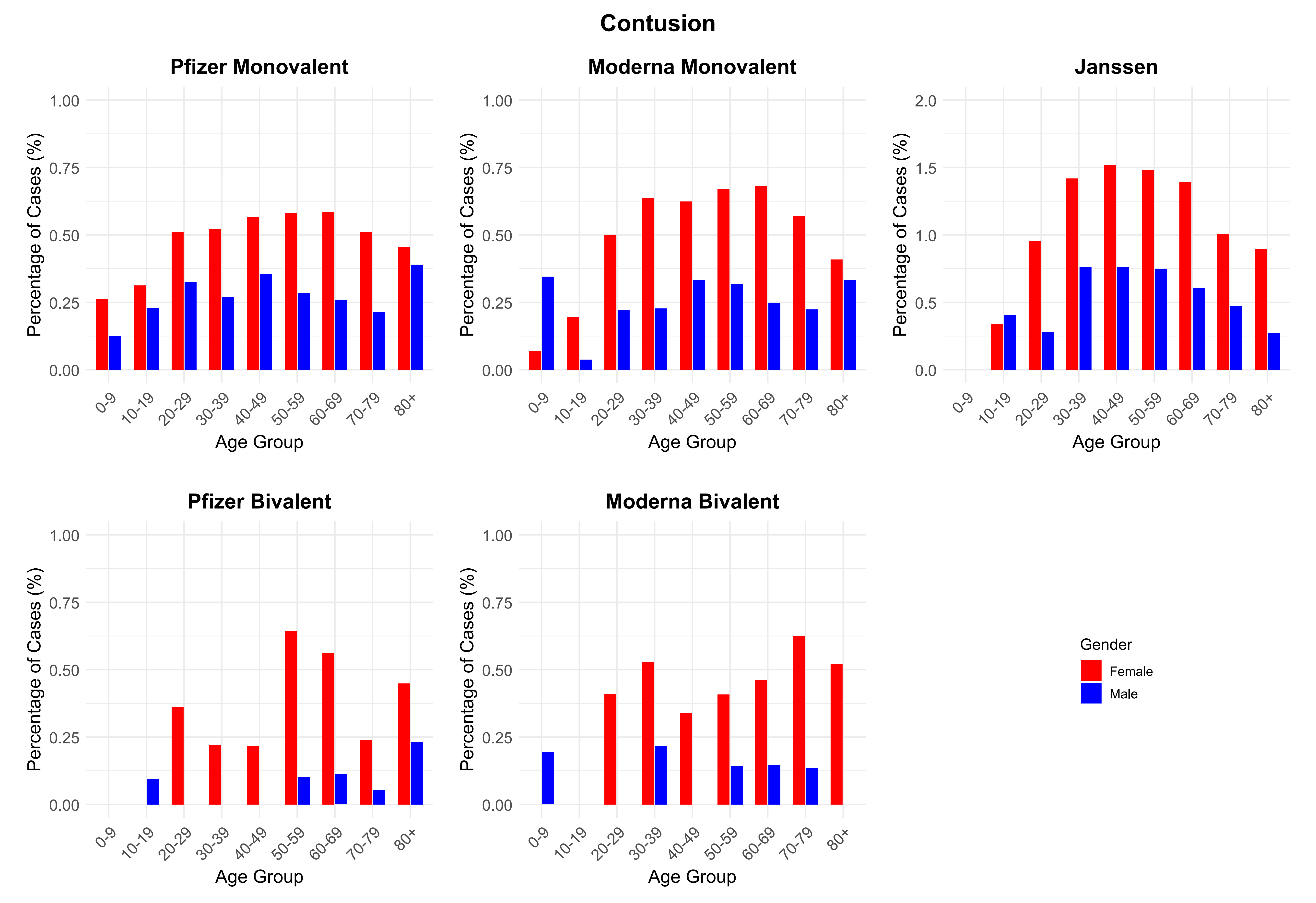

Contusion
Pfizer Monovalent
Moderna Monovalent
Janssen
Pfizer Bivalent
Moderna Bivalent
Percentage of Cases (%)
Age Group
0-9
10-19
20-29
30-39
40-49
50-59
60-69
70-79
80+
0.00
0.25
0.50
0.75
1.00
0.0
0.5
1.0
1.5
2.0
Gender
Female
Male

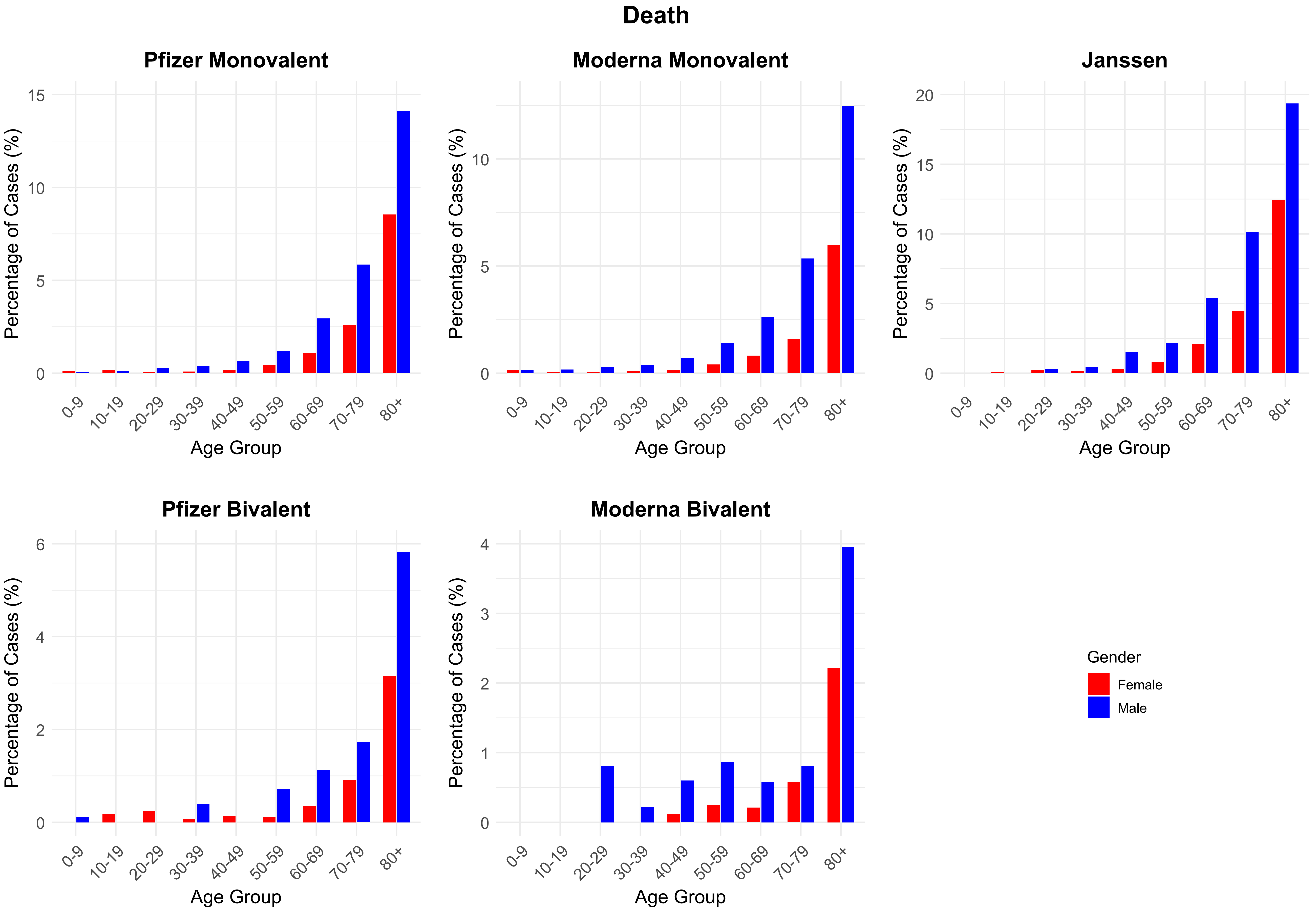

Death
Pfizer Monovalent
Moderna Monovalent
Janssen
Pfizer Bivalent
Moderna Bivalent
Percentage of Cases (%)
Age Group
0-9
10-19
20-29
30-39
40-49
50-59
60-69
70-79
80+
Gender
Female
Male

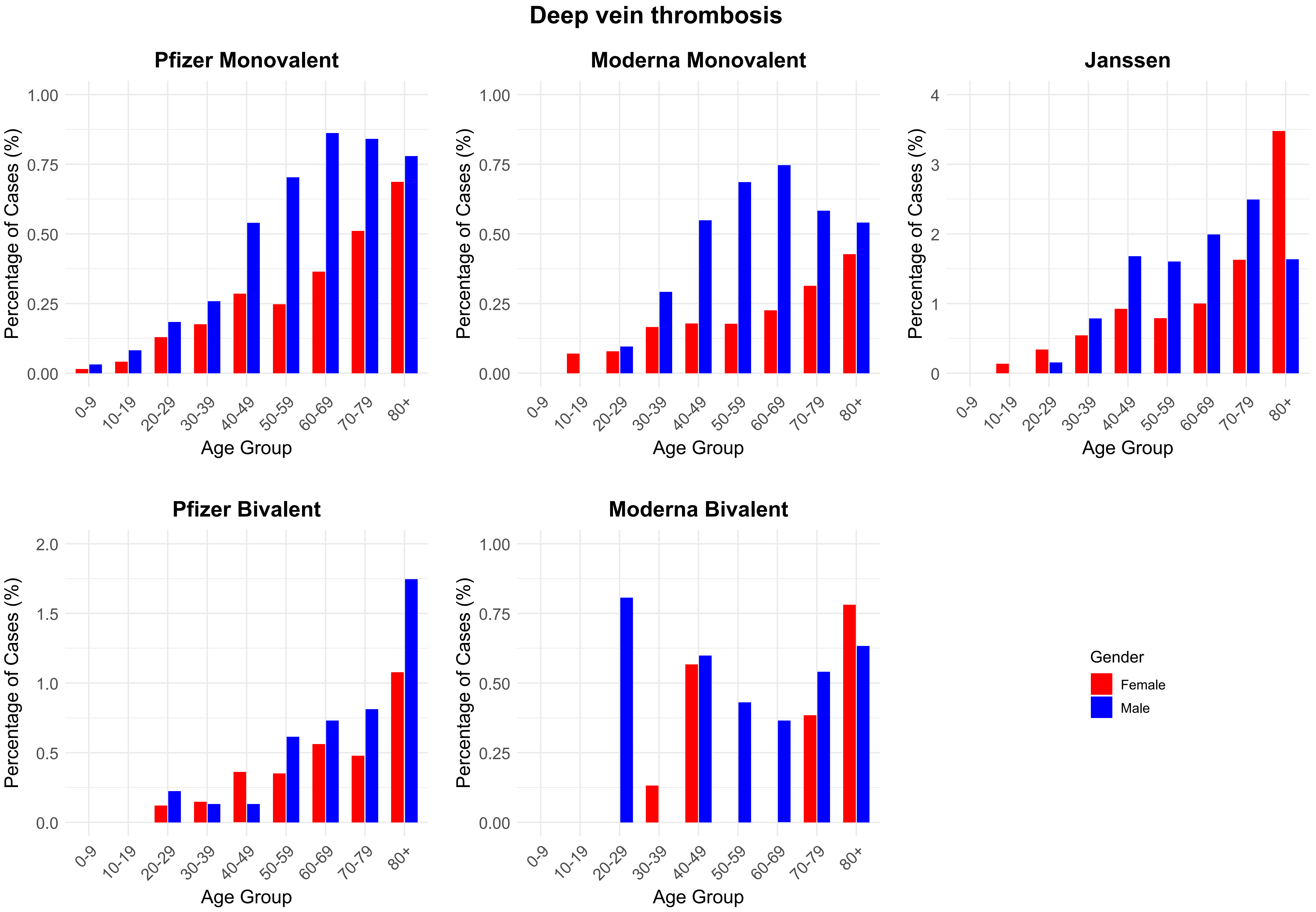
Deep vein thrombosis
Pfizer Monovalent
Moderna Monovalent
Janssen
Pfizer Bivalent
Moderna Bivalent
Percentage of Cases (%)
Age Group
0-9
10-19
20-29
30-39
40-49
50-59
60-69
70-79
80+
Gender
Female
Male

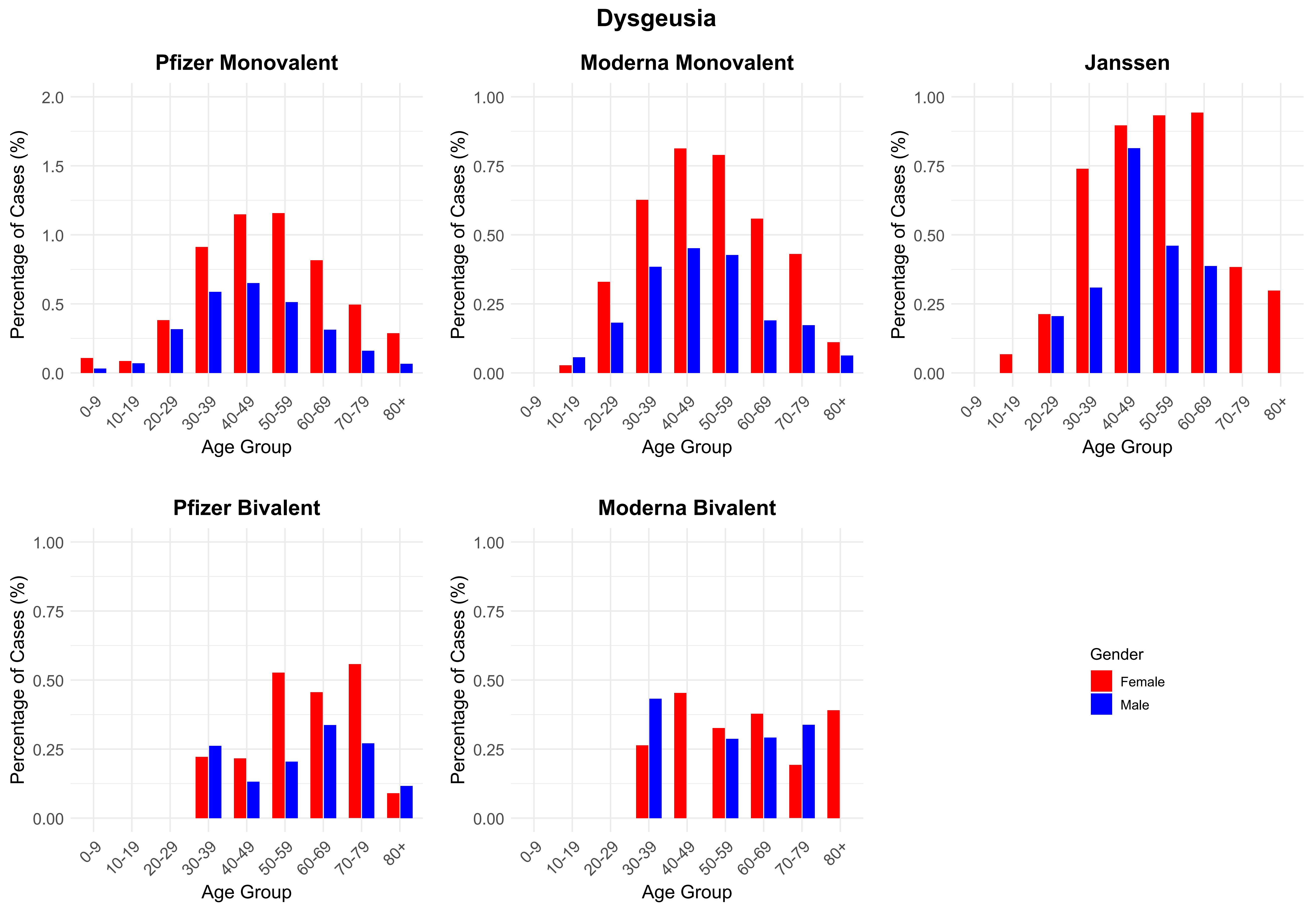
Dysgeusia
Pfizer Monovalent
Moderna Monovalent
Janssen
Pfizer Bivalent
Moderna Bivalent
Percentage of Cases (%)
Age Group
0-9
10-19
20-29
30-39
40-49
50-59
60-69
70-79
80+
Gender
Female
Male

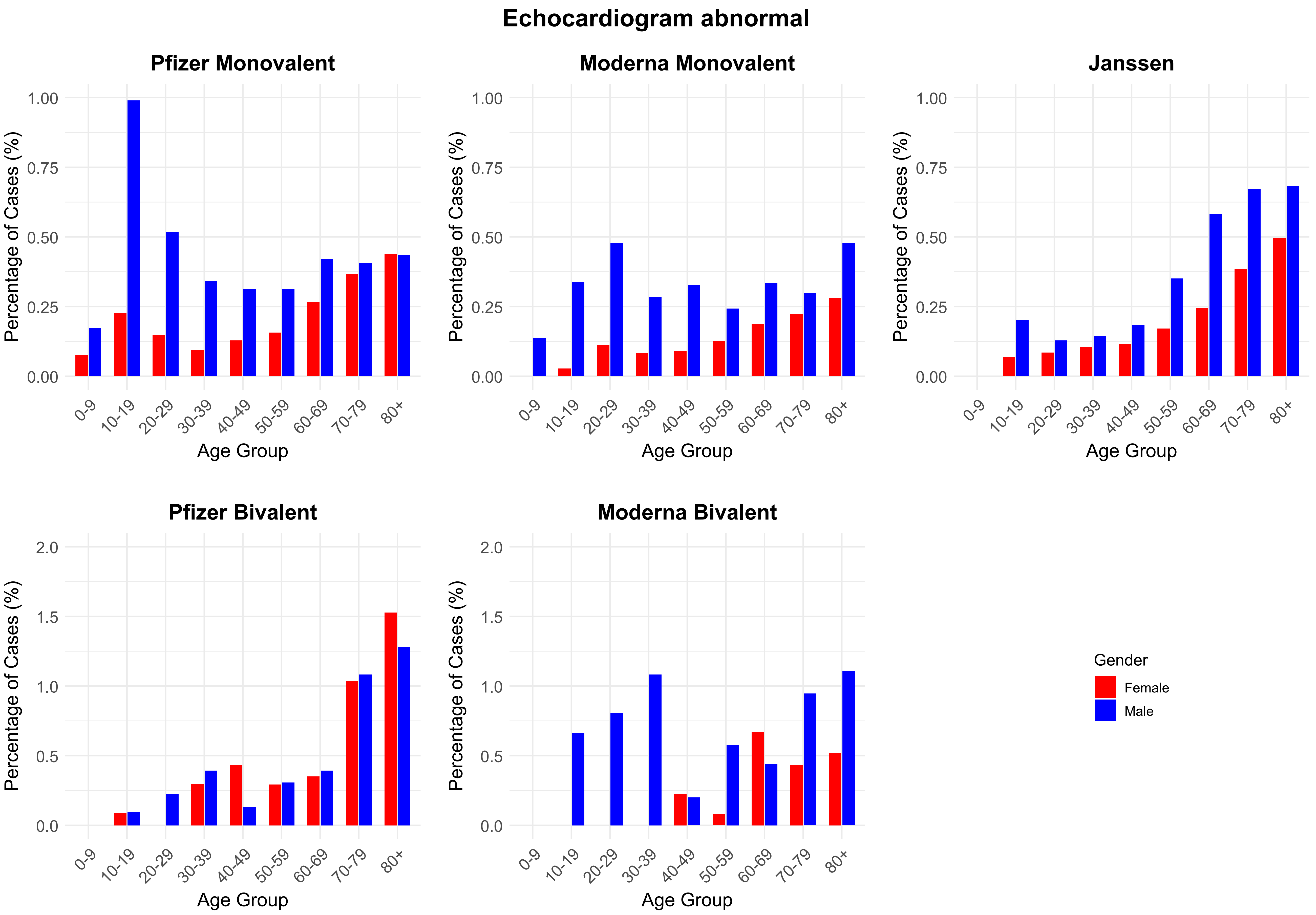

Echocardiogram abnormal
Pfizer Monovalent
Moderna Monovalent
Janssen
Pfizer Bivalent
Moderna Bivalent
Percentage of Cases (%)
Age Group
0-9
10-19
20-29
30-39
40-49
50-59
60-69
70-79
80+
0.00
0.25
0.50
0.75
1.00
0.0
0.5
1.0
1.5
2.0
Gender
Female
Male

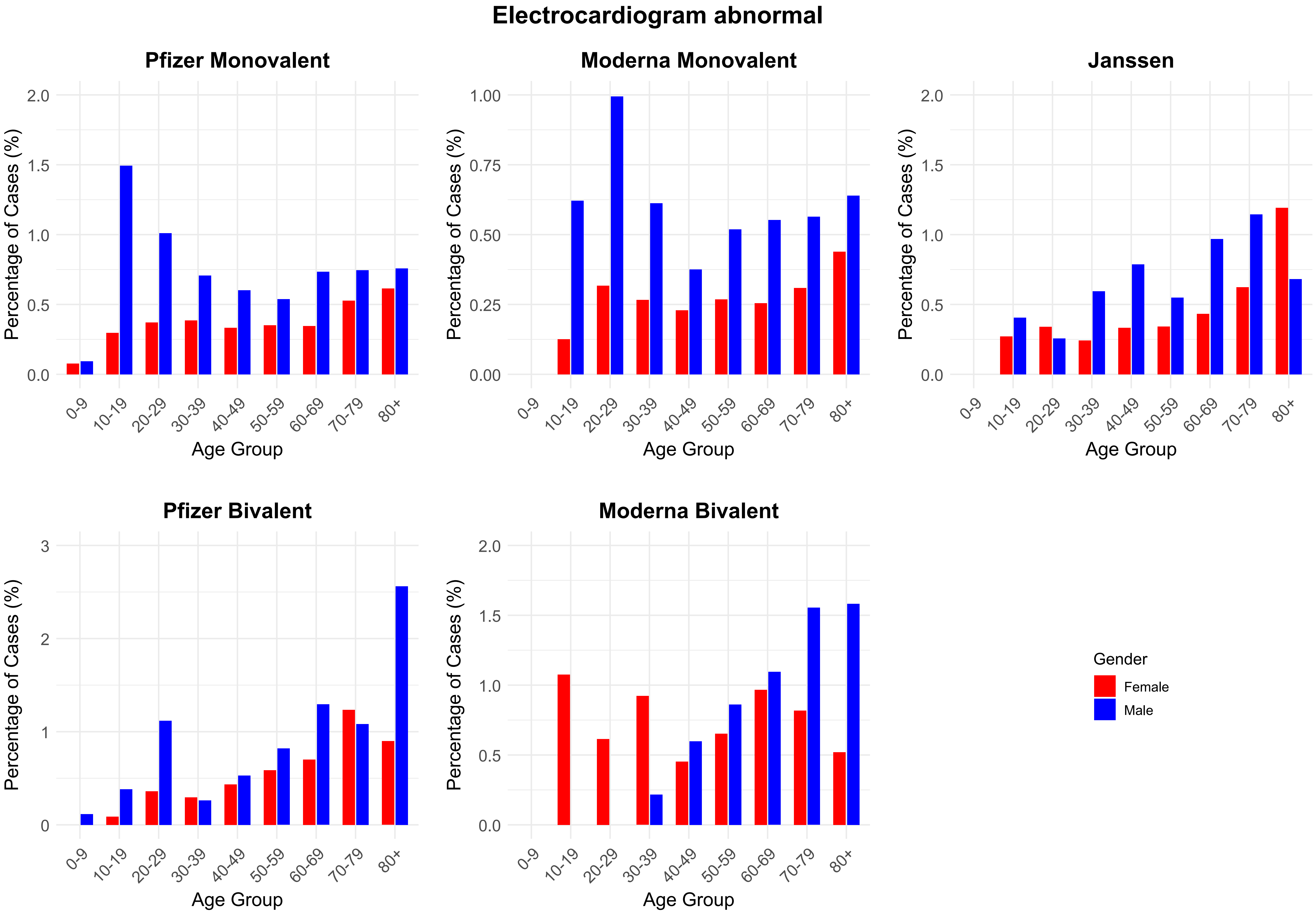
Electrocardiogram abnormal
Pfizer Monovalent
Moderna Monovalent
Janssen
Pfizer Bivalent
Moderna Bivalent
Percentage of Cases (%)
Age Group
0-9
10-19
20-29
30-39
40-49
50-59
60-69
70-79
80+
Gender
Female
Male

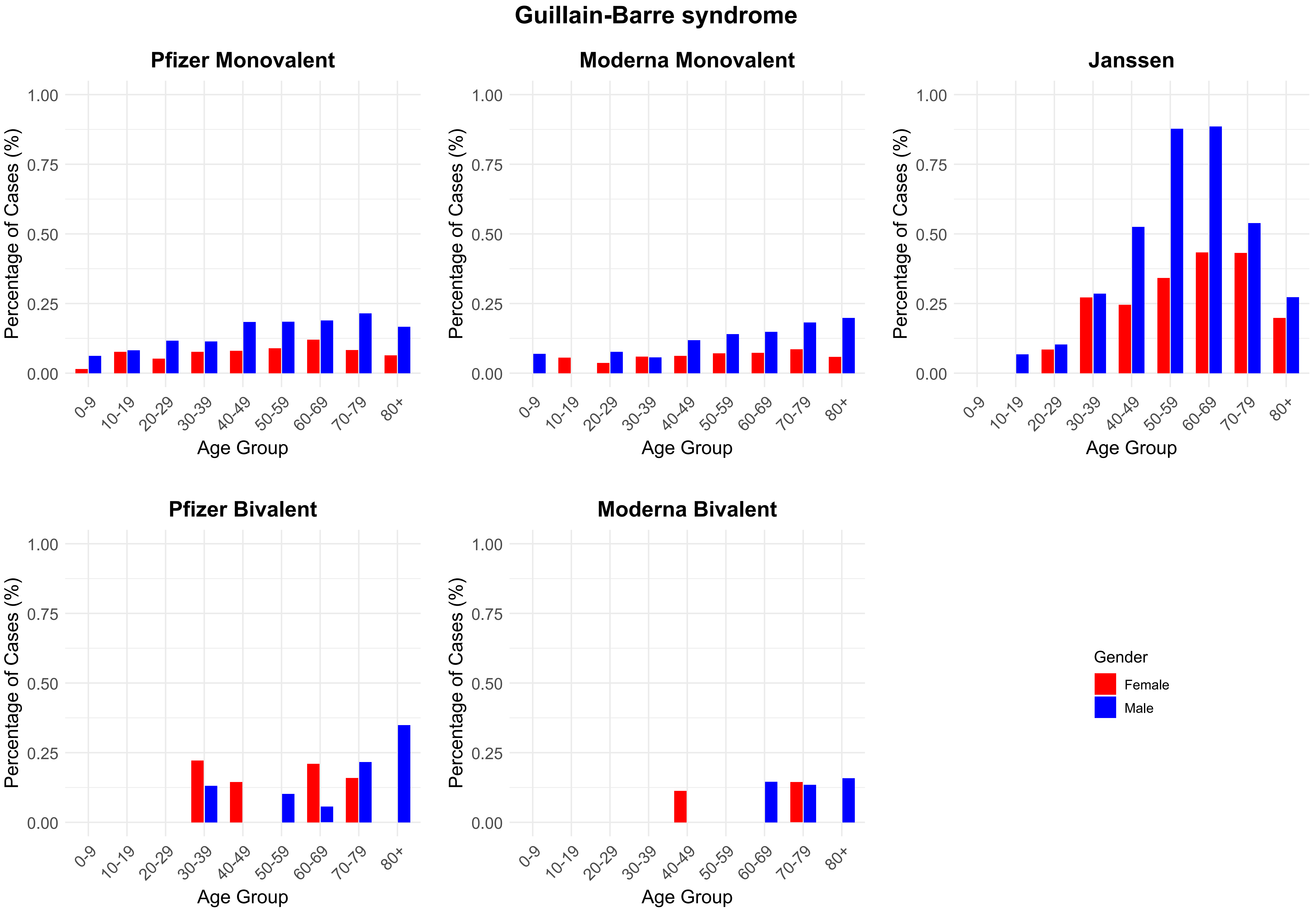
Guillain-Barre syndrome
Pfizer Monovalent
Moderna Monovalent
Janssen
Pfizer Bivalent
Moderna Bivalent
Percentage of Cases (%)
1.00
0.75
0.50
0.25
0.00
0-9
10-19
20-29
30-39
40-49
50-59
60-69
70-79
80+
Age Group
Gender
Female
Male

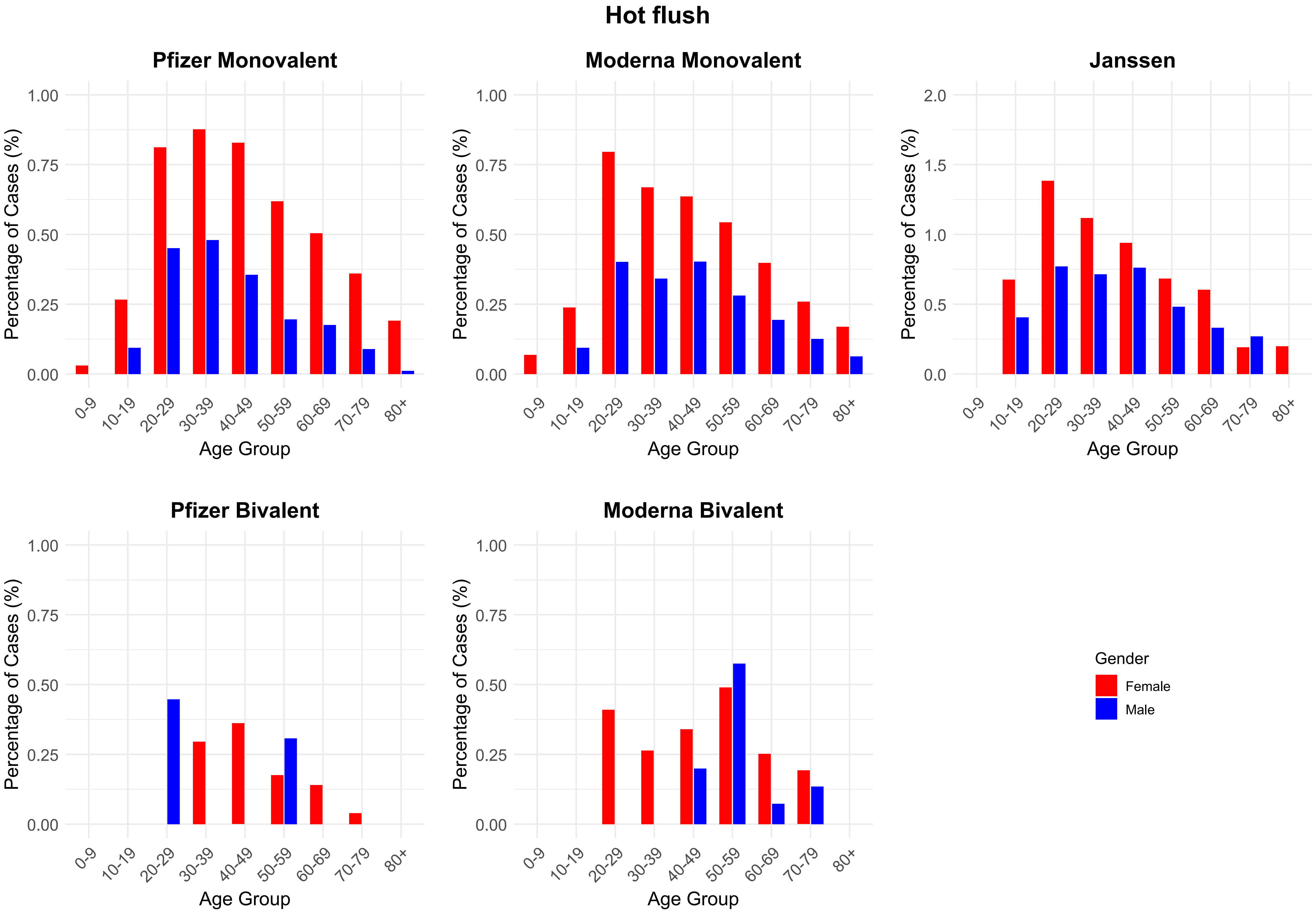

Hot flush
Pfizer Monovalent
Moderna Monovalent
Janssen
Pfizer Bivalent
Moderna Bivalent
Percentage of Cases (%)
Age Group
0-9
10-19
20-29
30-39
40-49
50-59
60-69
70-79
80+
0.00
0.25
0.50
0.75
1.00
0.0
0.5
1.0
1.5
2.0
Gender
Female
Male

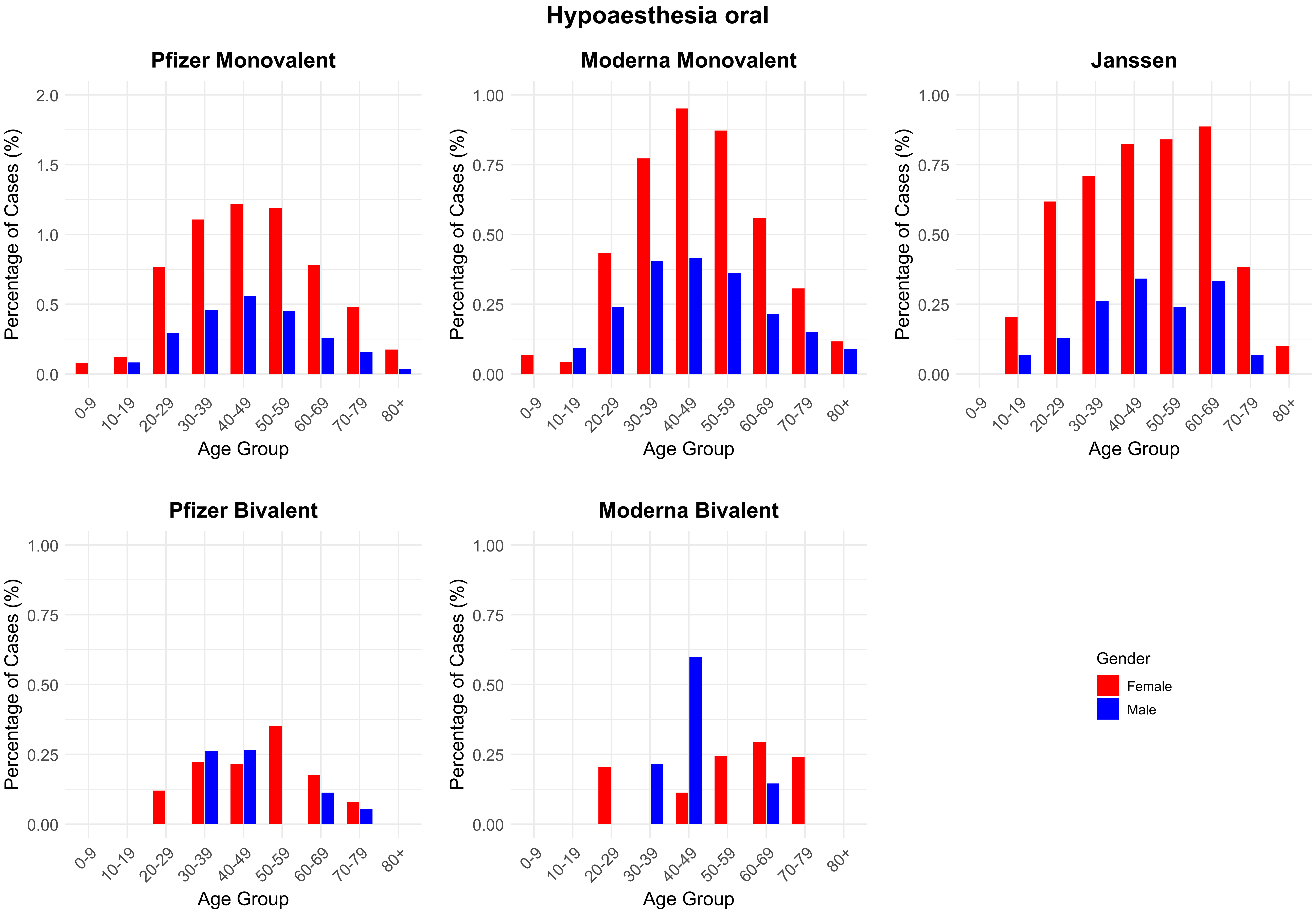
Hypoaesthesia oral
Pfizer Monovalent
Moderna Monovalent
Janssen
Pfizer Bivalent
Moderna Bivalent
Percentage of Cases (%)
Age Group
0-9
10-19
20-29
30-39
40-49
50-59
60-69
70-79
80+
0.0
0.5
1.0
1.5
2.0
0.00
0.25
0.50
0.75
1.00
Gender
Female
Male

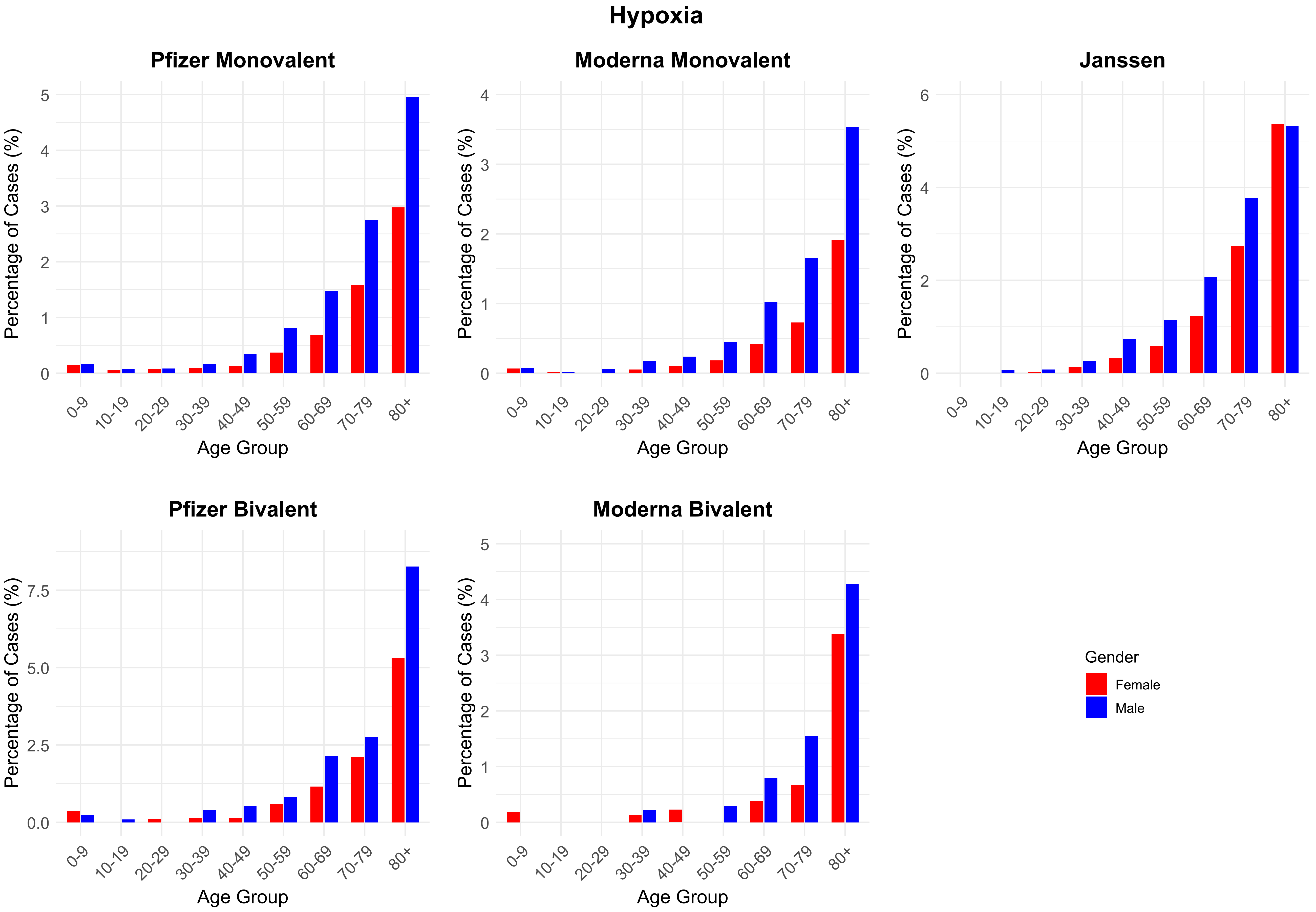

Hypoxia
Pfizer Monovalent
Moderna Monovalent
Janssen
Pfizer Bivalent
Moderna Bivalent
Percentage of Cases (%)
Age Group
0-9
10-19
20-29
30-39
40-49
50-59
60-69
70-79
80+
Gender
Female
Male

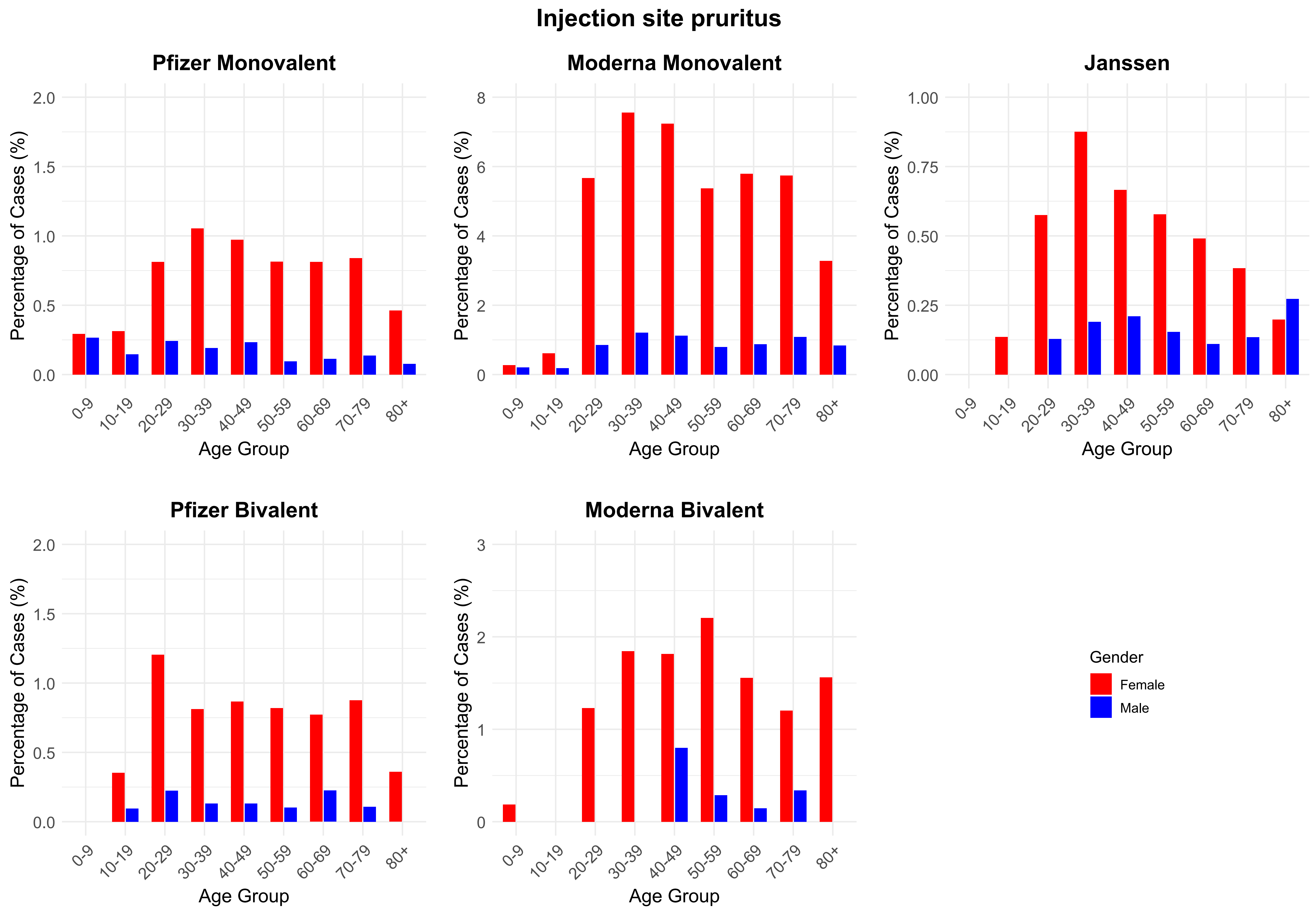
Injection site pruritus
Pfizer Monovalent
Moderna Monovalent
Janssen
Pfizer Bivalent
Moderna Bivalent
Percentage of Cases (%)
Age Group
0-9
10-19
20-29
30-39
40-49
50-59
60-69
70-79
80+
Gender
Female
Male

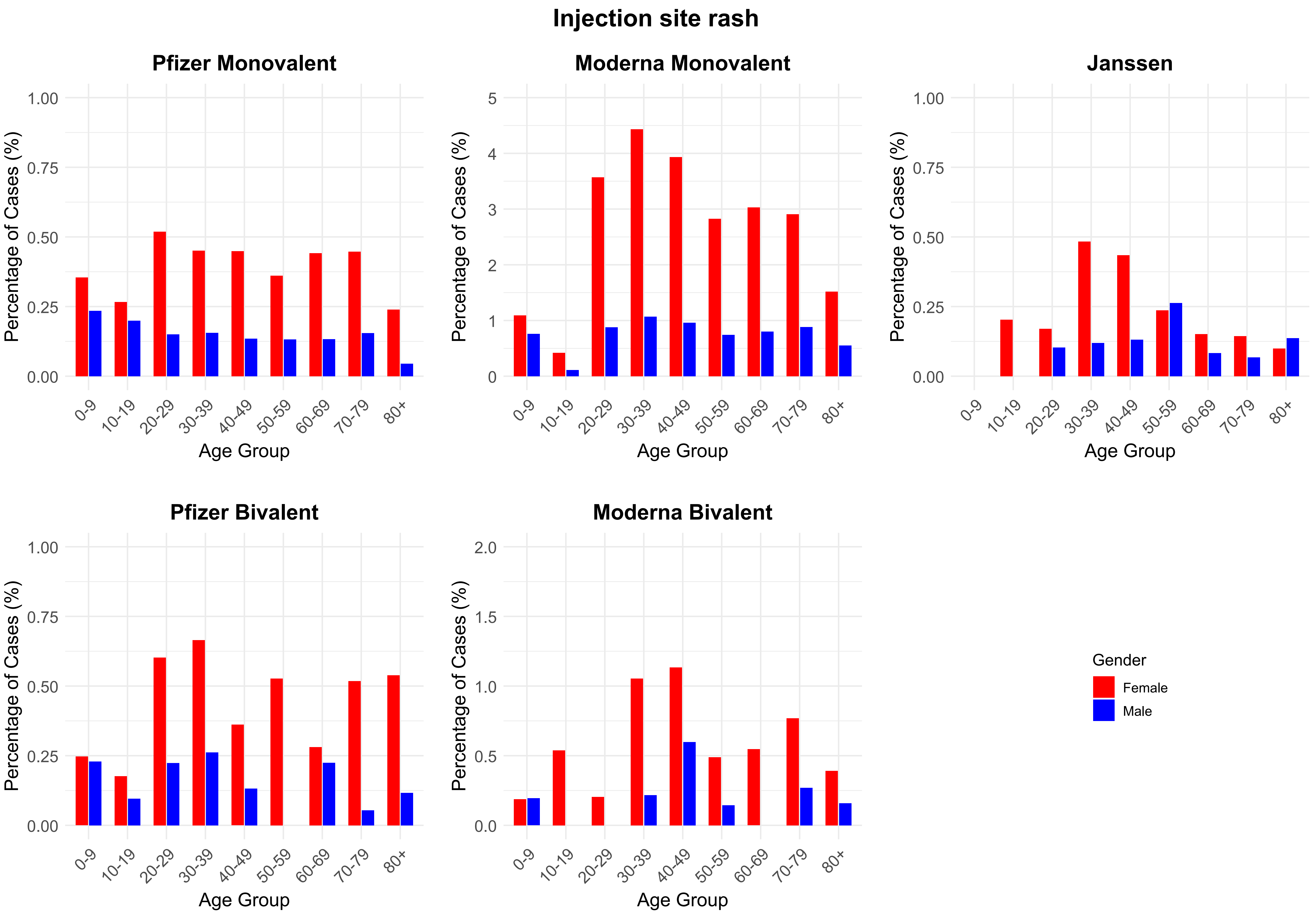

Injection site rash
Pfizer Monovalent
Moderna Monovalent
Janssen
Pfizer Bivalent
Moderna Bivalent
Percentage of Cases (%)
Age Group
0-9
10-19
20-29
30-39
40-49
50-59
60-69
70-79
80+
Gender
Female
Male

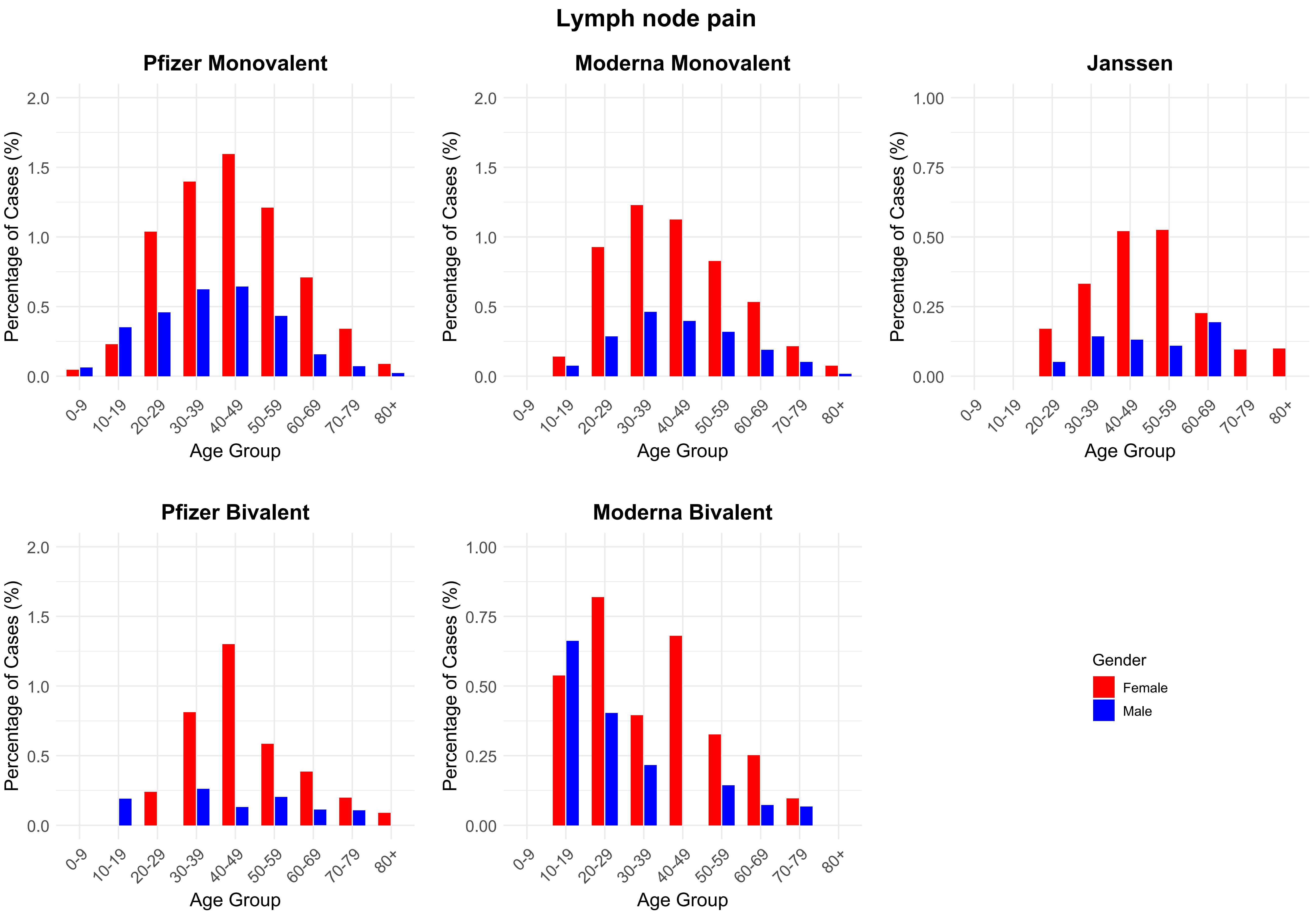
Lymph node pain
Pfizer Monovalent
Moderna Monovalent
Janssen
Pfizer Bivalent
Moderna Bivalent
Percentage of Cases (%)
Age Group
0-9
10-19
20-29
30-39
40-49
50-59
60-69
70-79
80+
Gender
Female
Male

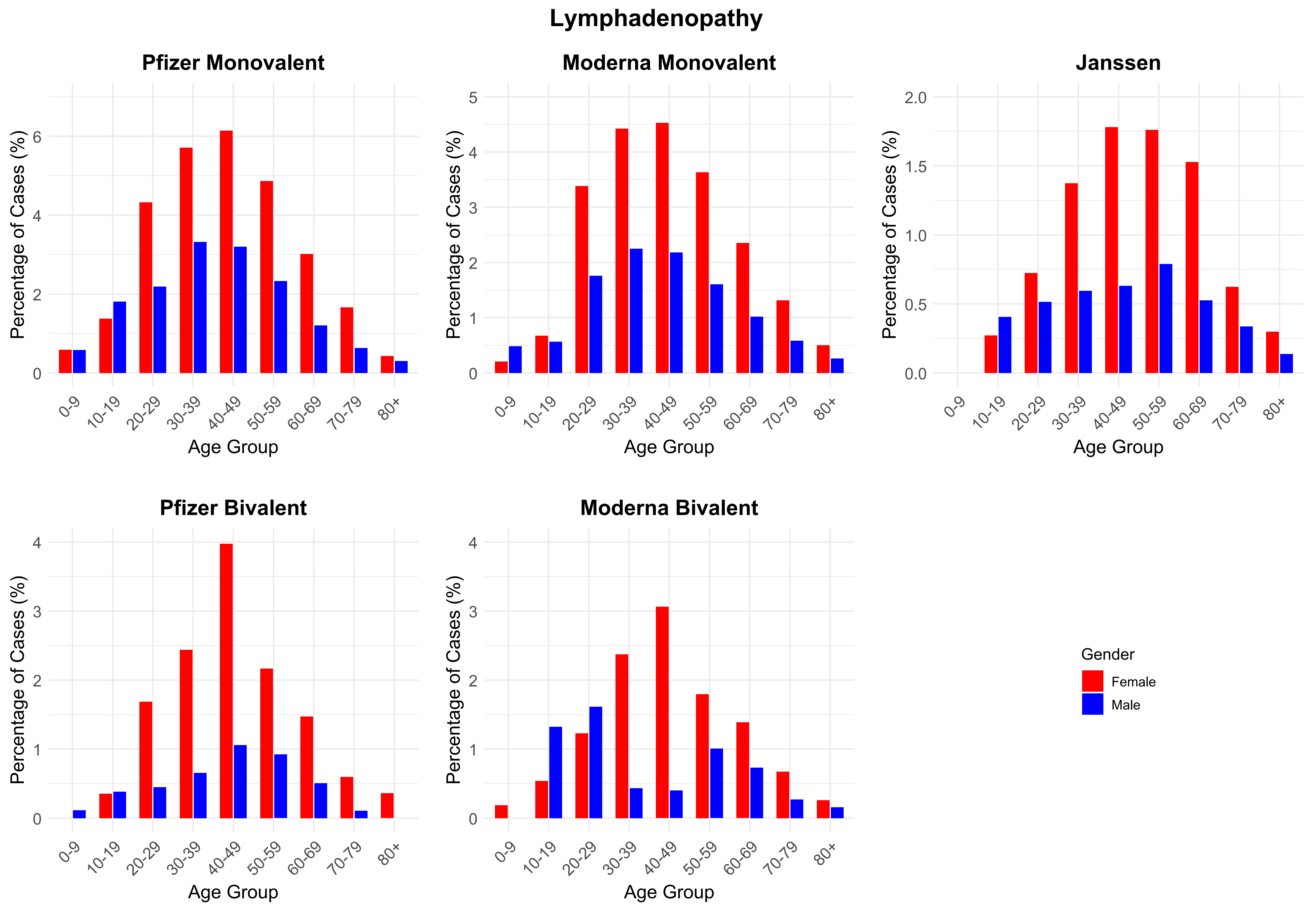

Lymphadenopathy
Pfizer Monovalent
Moderna Monovalent
Janssen
Pfizer Bivalent
Moderna Bivalent
Percentage of Cases (%)
Age Group
0-9
10-19
20-29
30-39
40-49
50-59
60-69
70-79
80+
Gender
Female
Male

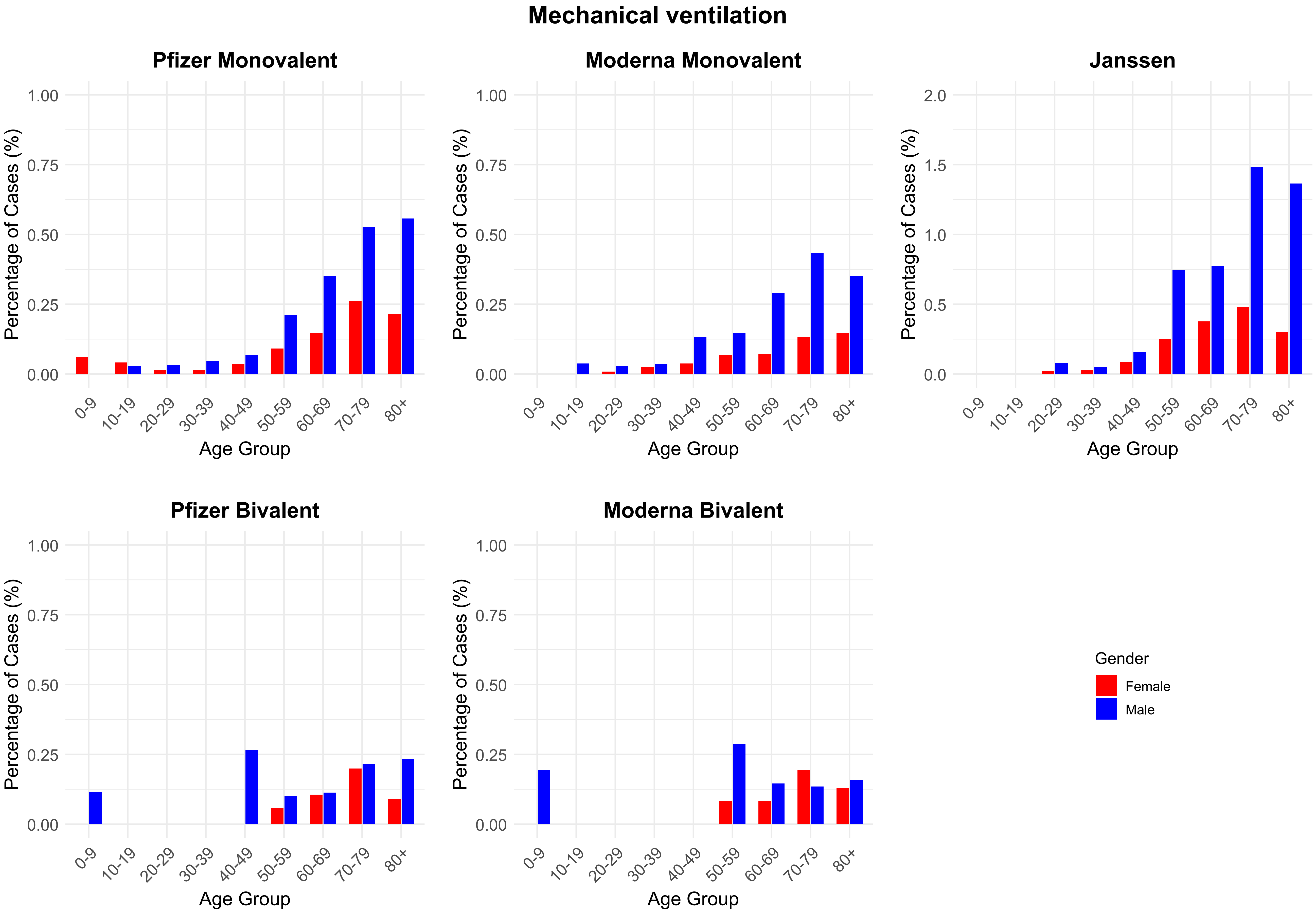

Mechanical ventilation
Pfizer Monovalent
Moderna Monovalent
Janssen
Pfizer Bivalent
Moderna Bivalent
Percentage of Cases (%)
Age Group
0-9
10-19
20-29
30-39
40-49
50-59
60-69
70-79
80+
0.00
0.25
0.50
0.75
1.00
0.0
0.5
1.0
1.5
2.0
Gender
Female
Male

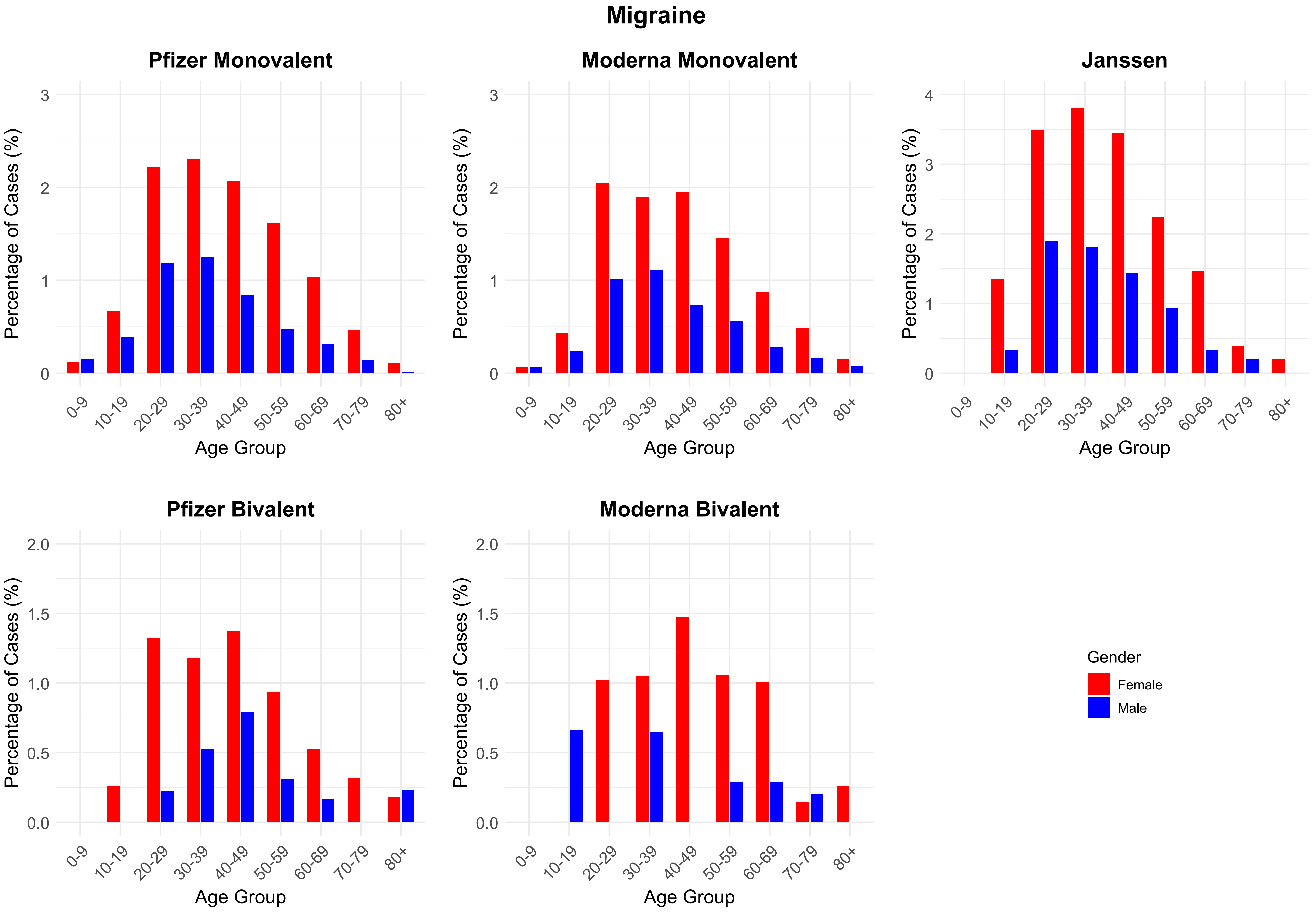

Migraine
Pfizer Monovalent
Moderna Monovalent
Janssen
Pfizer Bivalent
Moderna Bivalent
Percentage of Cases (%)
Age Group
0-9
10-19
20-29
30-39
40-49
50-59
60-69
70-79
80+
Gender
Female
Male

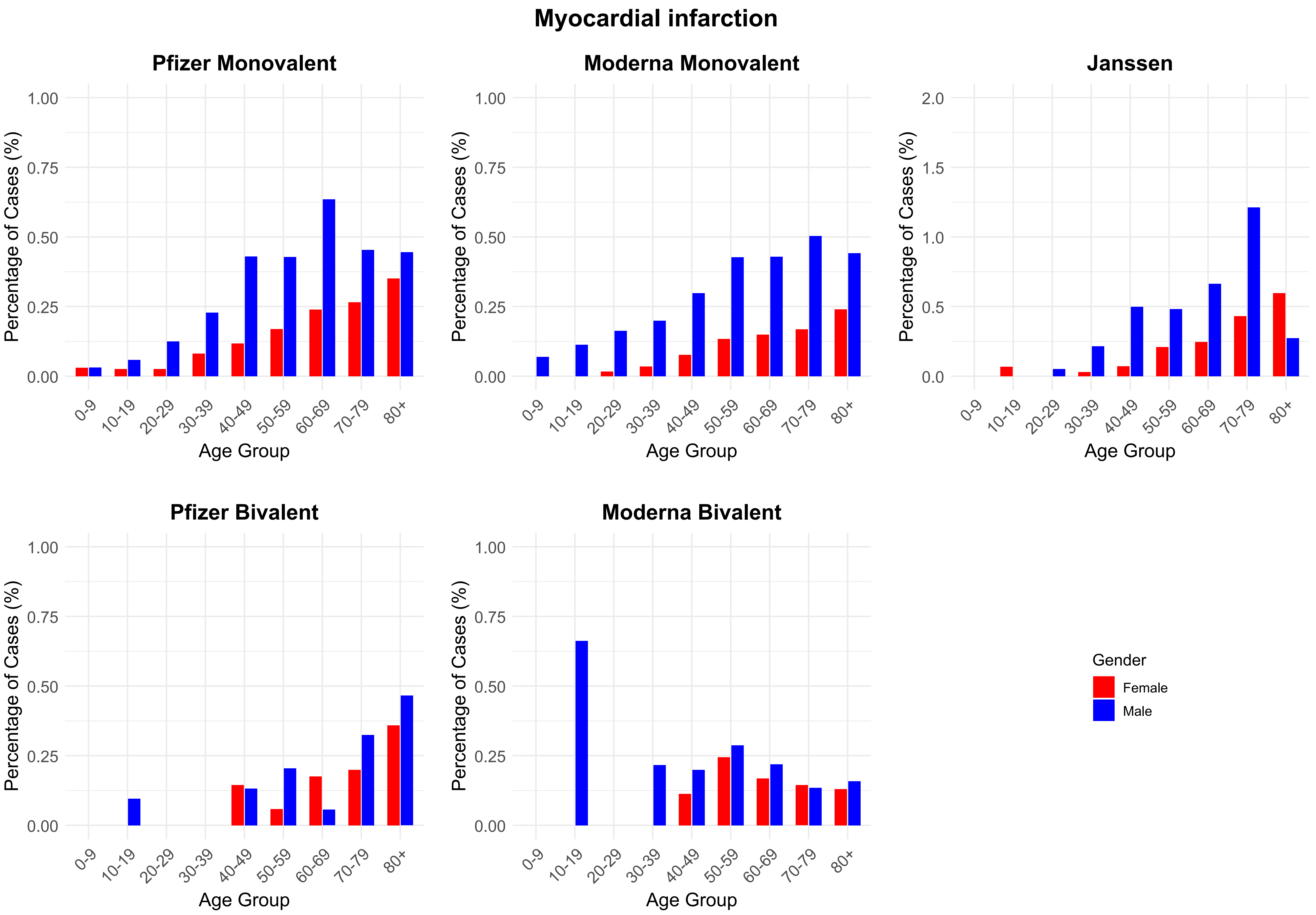
Myocardial infarction
Pfizer Monovalent
Moderna Monovalent
Janssen
Pfizer Bivalent
Moderna Bivalent
Percentage of Cases (%)
Age Group
0-9
10-19
20-29
30-39
40-49
50-59
60-69
70-79
80+
0.00
0.25
0.50
0.75
1.00
0.0
0.5
1.0
1.5
2.0
Gender
Female
Male

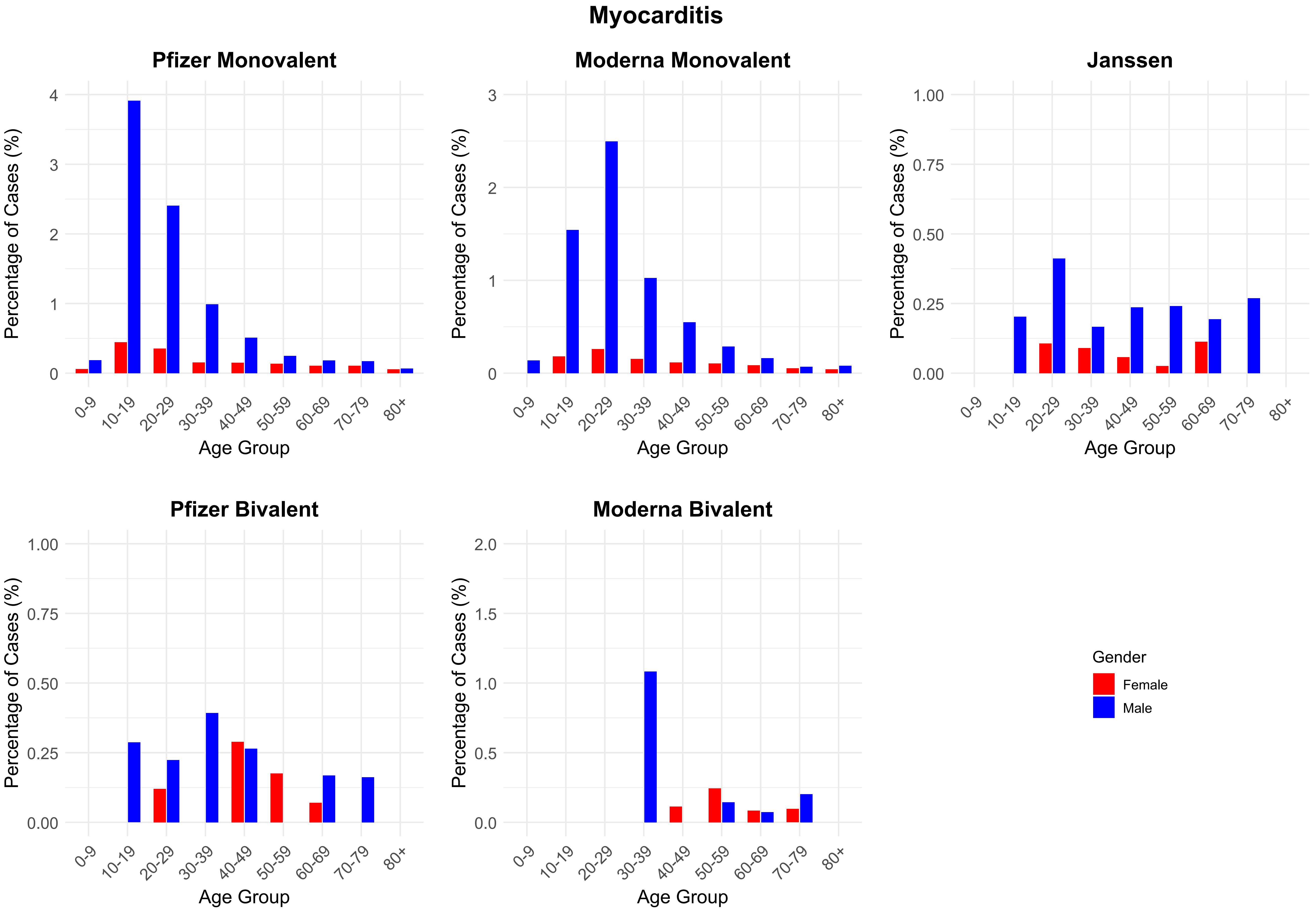
Myocarditis
Pfizer Monovalent
Moderna Monovalent
Janssen
Pfizer Bivalent
Moderna Bivalent
Percentage of Cases (%)
Age Group
0-9
10-19
20-29
30-39
40-49
50-59
60-69
70-79
80+
Gender
Female
Male

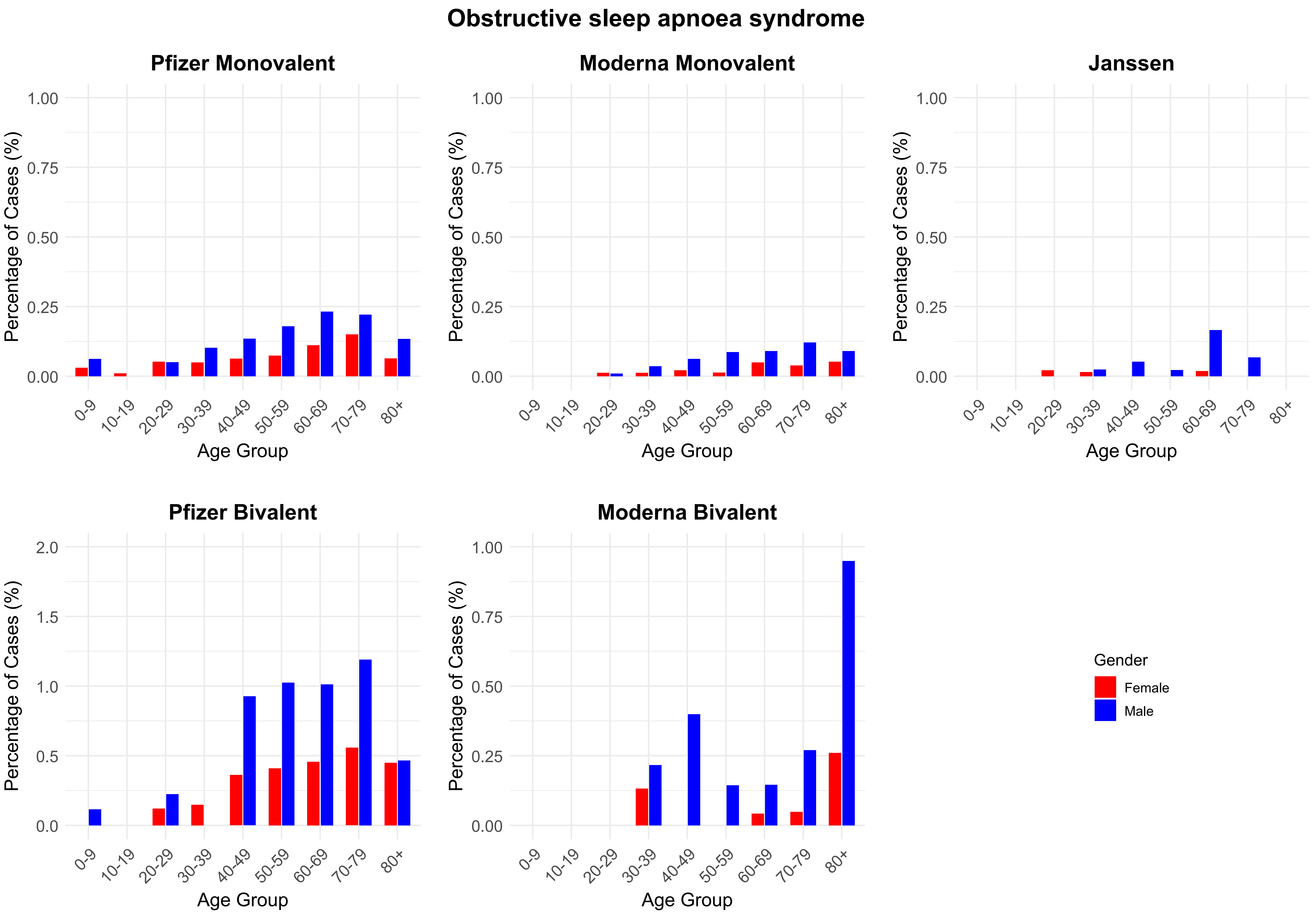
Obstructive sleep apnoea syndrome
Pfizer Monovalent
Moderna Monovalent
Janssen
Pfizer Bivalent
Moderna Bivalent
Percentage of Cases (%)
Age Group
0-9
10-19
20-29
30-39
40-49
50-59
60-69
70-79
80+
Gender
Female
Male

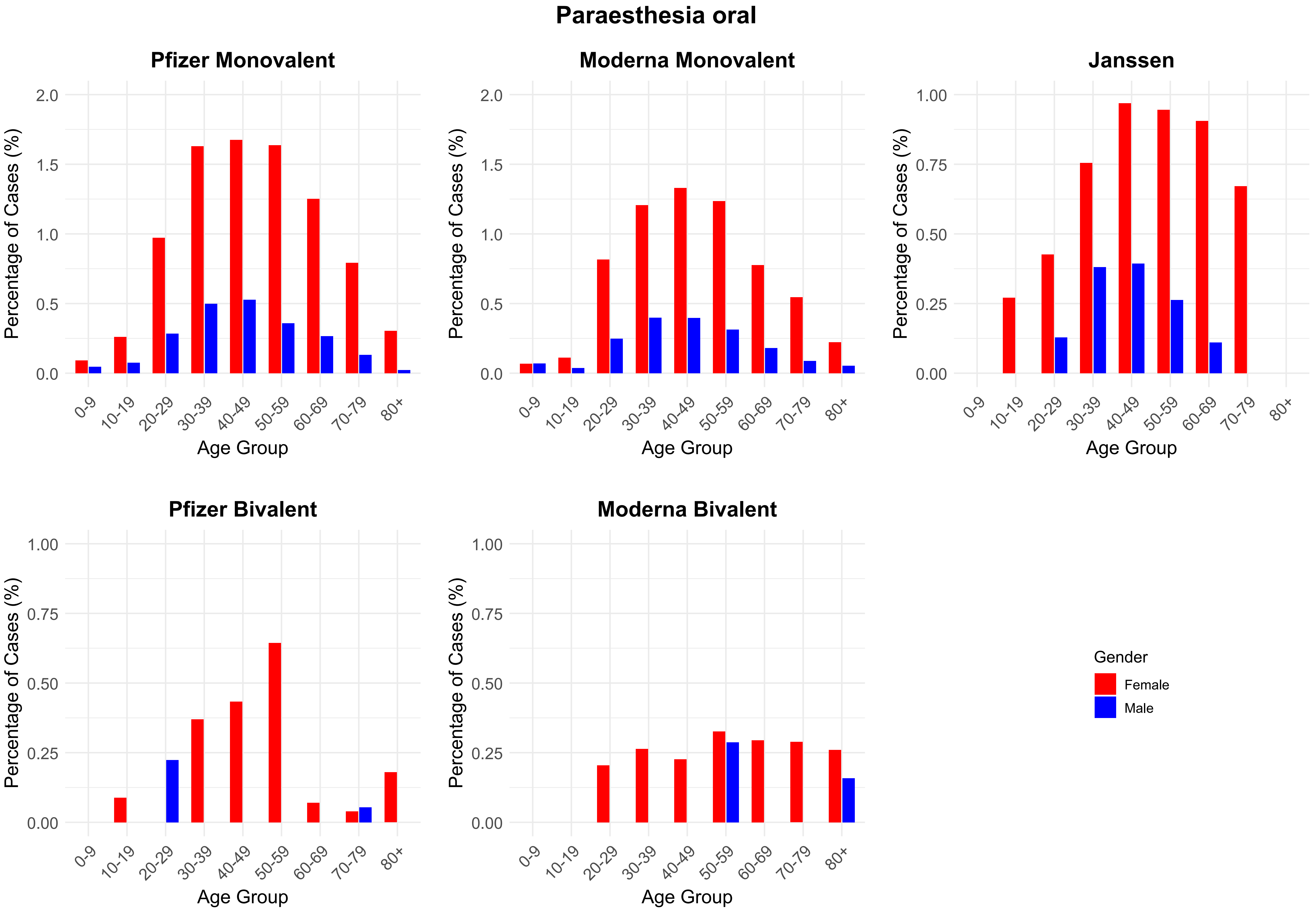
Paraesthesia oral
Pfizer Monovalent
Moderna Monovalent
Janssen
Pfizer Bivalent
Moderna Bivalent
Percentage of Cases (%)
Age Group
0-9
10-19
20-29
30-39
40-49
50-59
60-69
70-79
80+
Gender
Female
Male

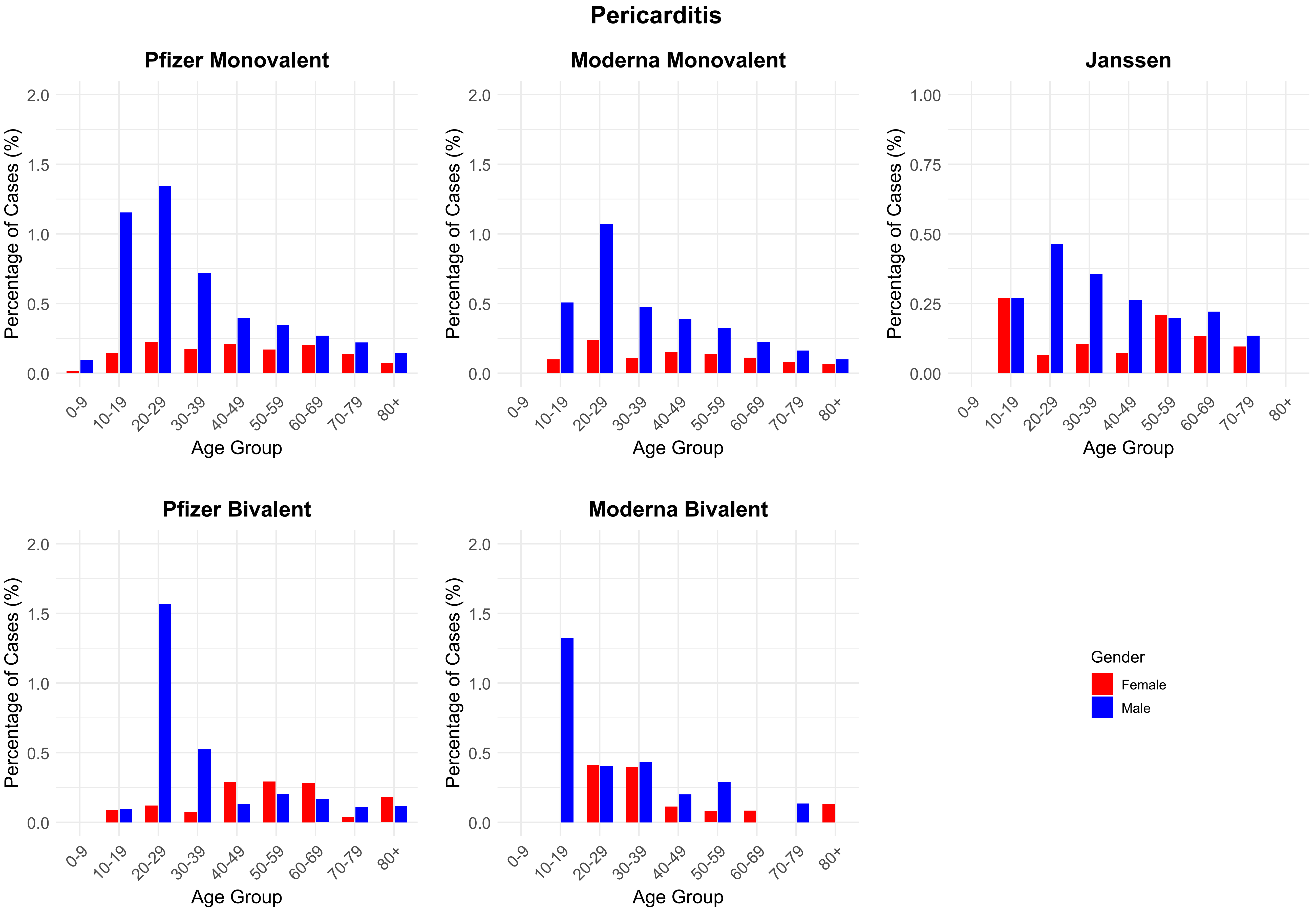
Pericarditis
Pfizer Monovalent
Moderna Monovalent
Janssen
Pfizer Bivalent
Moderna Bivalent
Percentage of Cases (%)
Age Group
0-9
10-19
20-29
30-39
40-49
50-59
60-69
70-79
80+
0.0
0.5
1.0
1.5
2.0
0.00
0.25
0.50
0.75
1.00
Gender
Female
Male

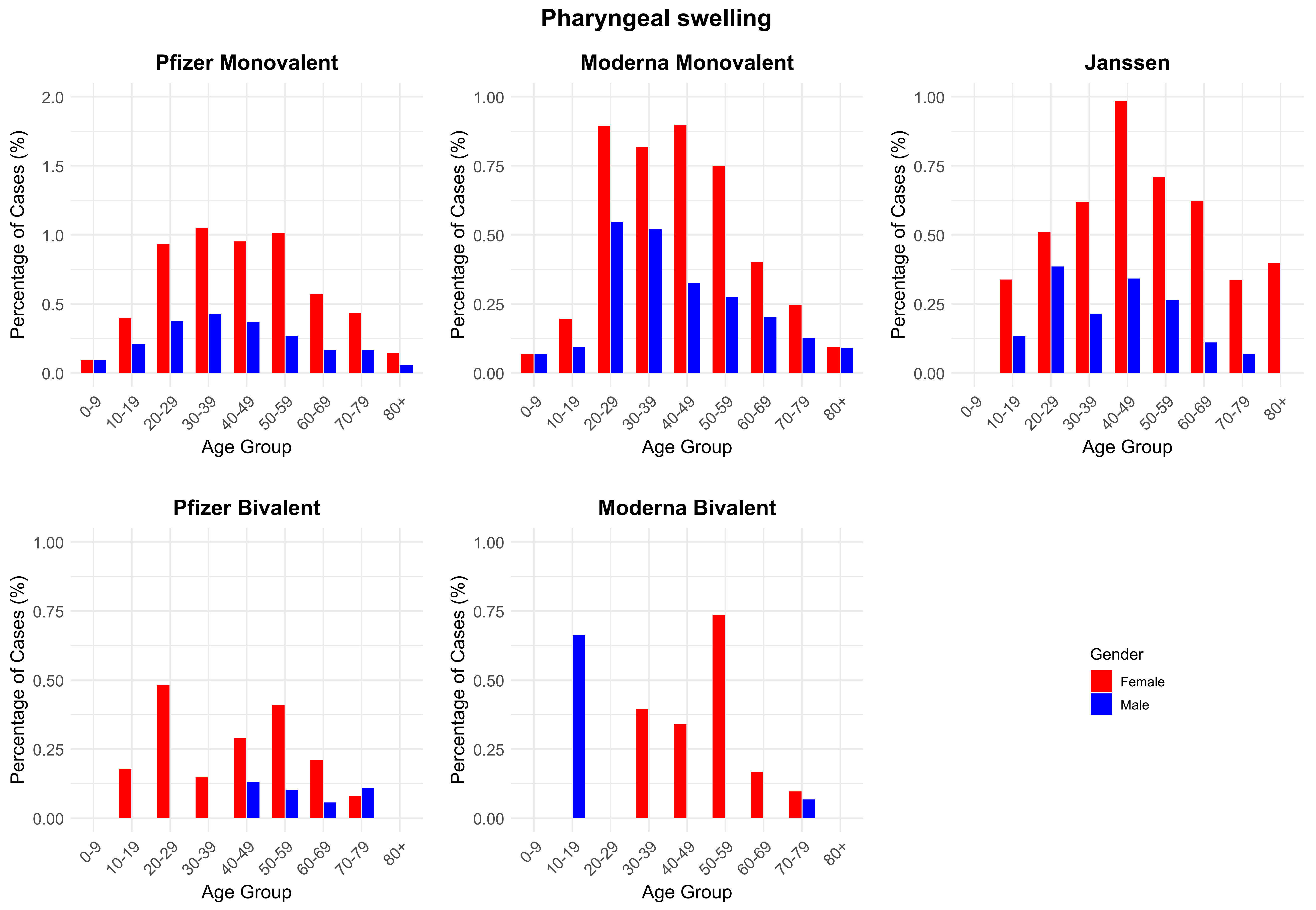

Pharyngeal swelling
Pfizer Monovalent
Moderna Monovalent
Janssen
Pfizer Bivalent
Moderna Bivalent
Percentage of Cases (%)
Age Group
0-9
10-19
20-29
30-39
40-49
50-59
60-69
70-79
80+
0.0
0.5
1.0
1.5
2.0
0.00
0.25
0.50
0.75
1.00
Gender
Female
Male

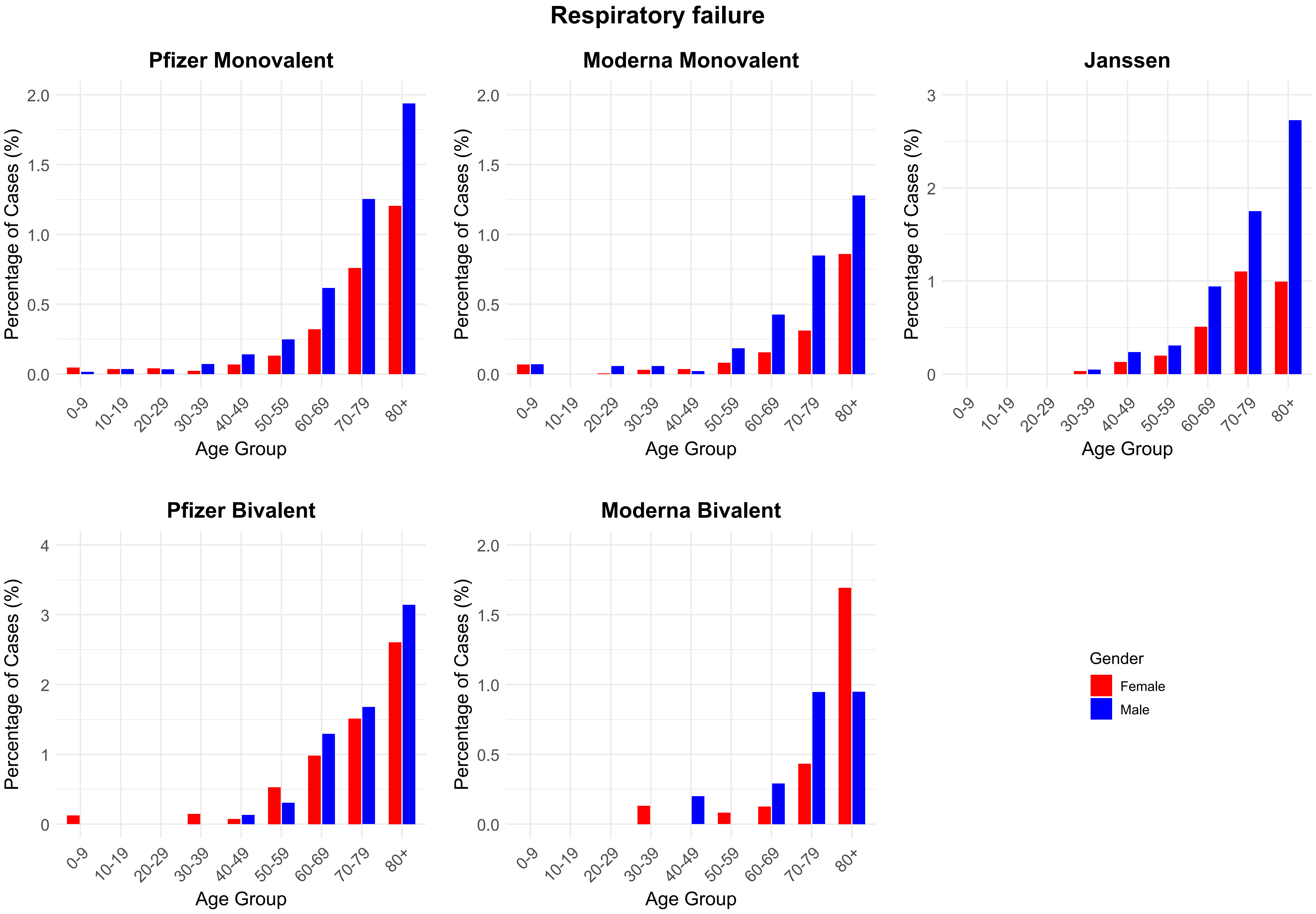

Respiratory failure
Pfizer Monovalent
Moderna Monovalent
Janssen
Pfizer Bivalent
Moderna Bivalent
Percentage of Cases (%)
Age Group
0-9
10-19
20-29
30-39
40-49
50-59
60-69
70-79
80+
Gender
Female
Male

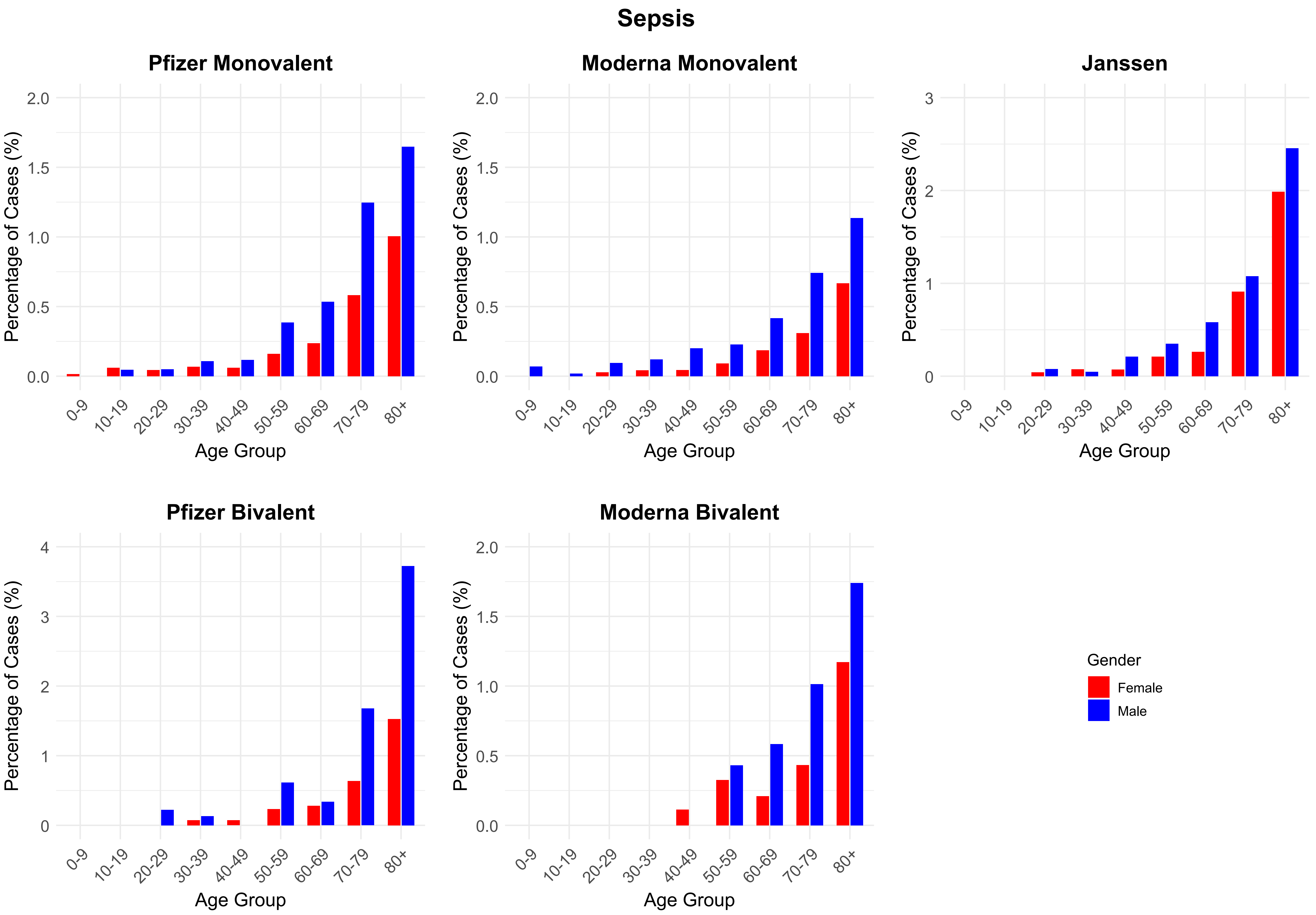

Sepsis
Pfizer Monovalent
Moderna Monovalent
Janssen
Pfizer Bivalent
Moderna Bivalent
Percentage of Cases (%)
Age Group
0-9
10-19
20-29
30-39
40-49
50-59
60-69
70-79
80+
Gender
Female
Male

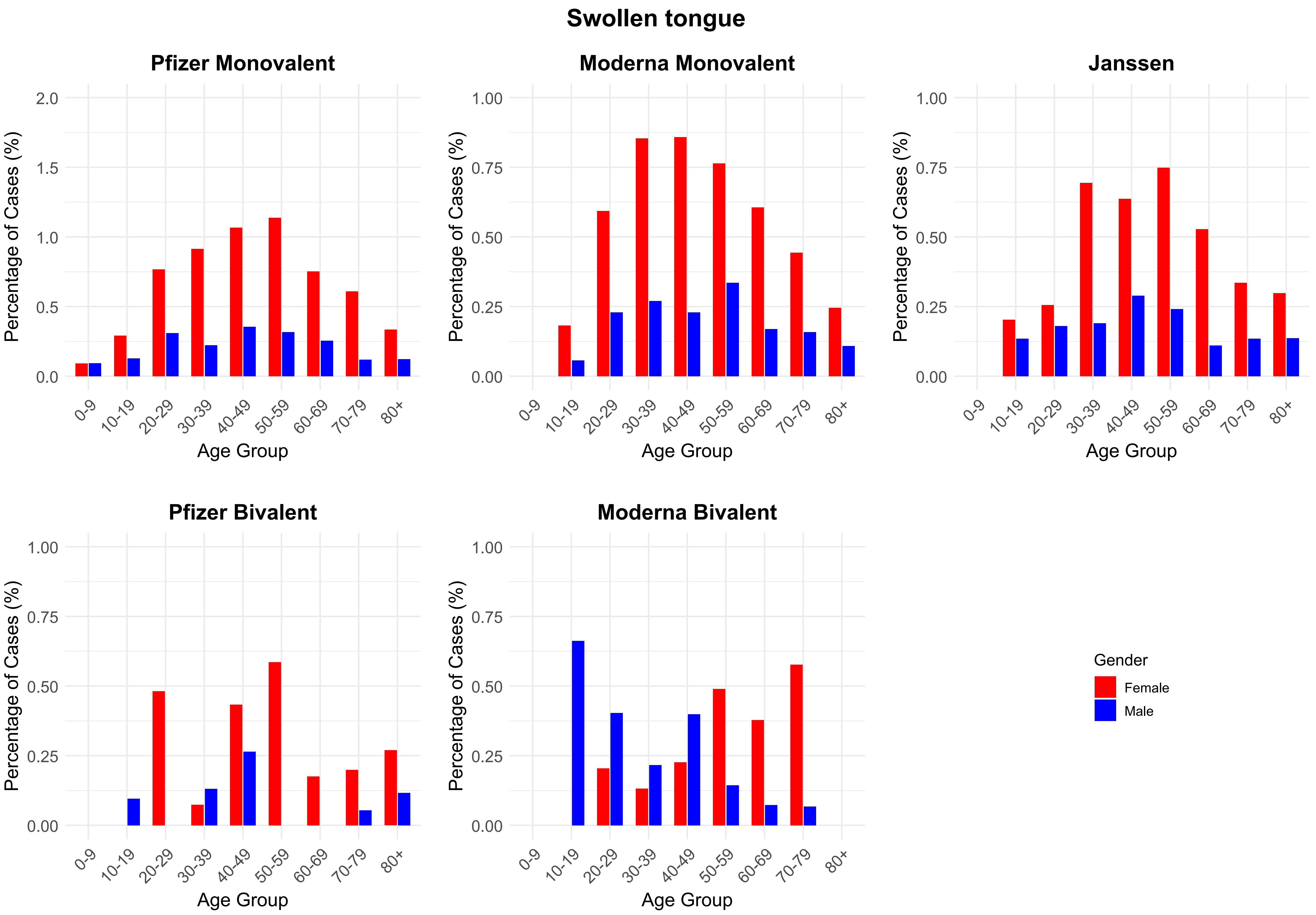
Swollen tongue
Pfizer Monovalent
Moderna Monovalent
Janssen
Pfizer Bivalent
Moderna Bivalent
Percentage of Cases (%)
Age Group
0-9
10-19
20-29
30-39
40-49
50-59
60-69
70-79
80+
Gender
Female
Male

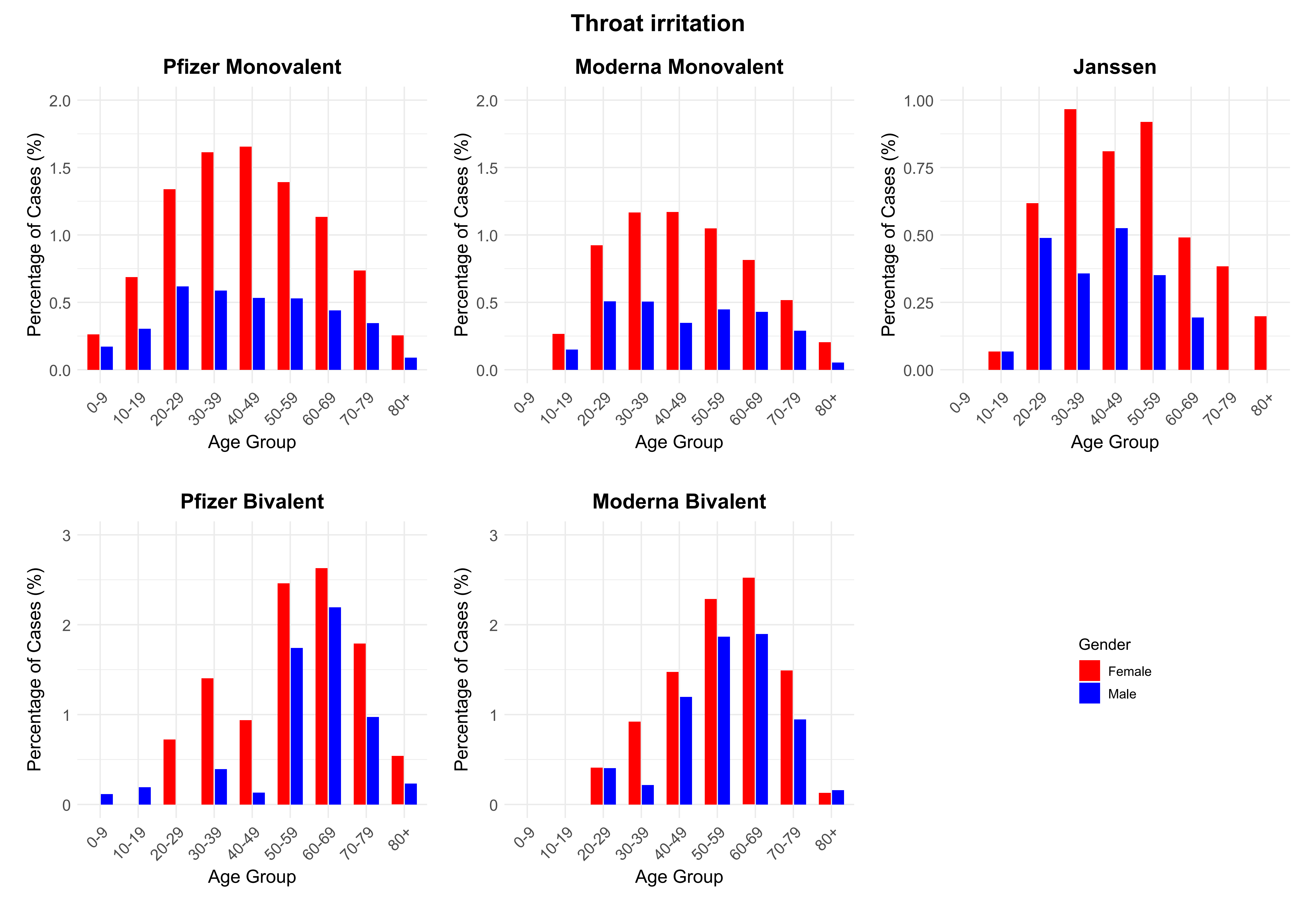
Throat irritation
Pfizer Monovalent
Moderna Monovalent
Janssen
Pfizer Bivalent
Moderna Bivalent
Percentage of Cases (%)
Age Group
0-9
10-19
20-29
30-39
40-49
50-59
60-69
70-79
80+
Gender
Female
Male

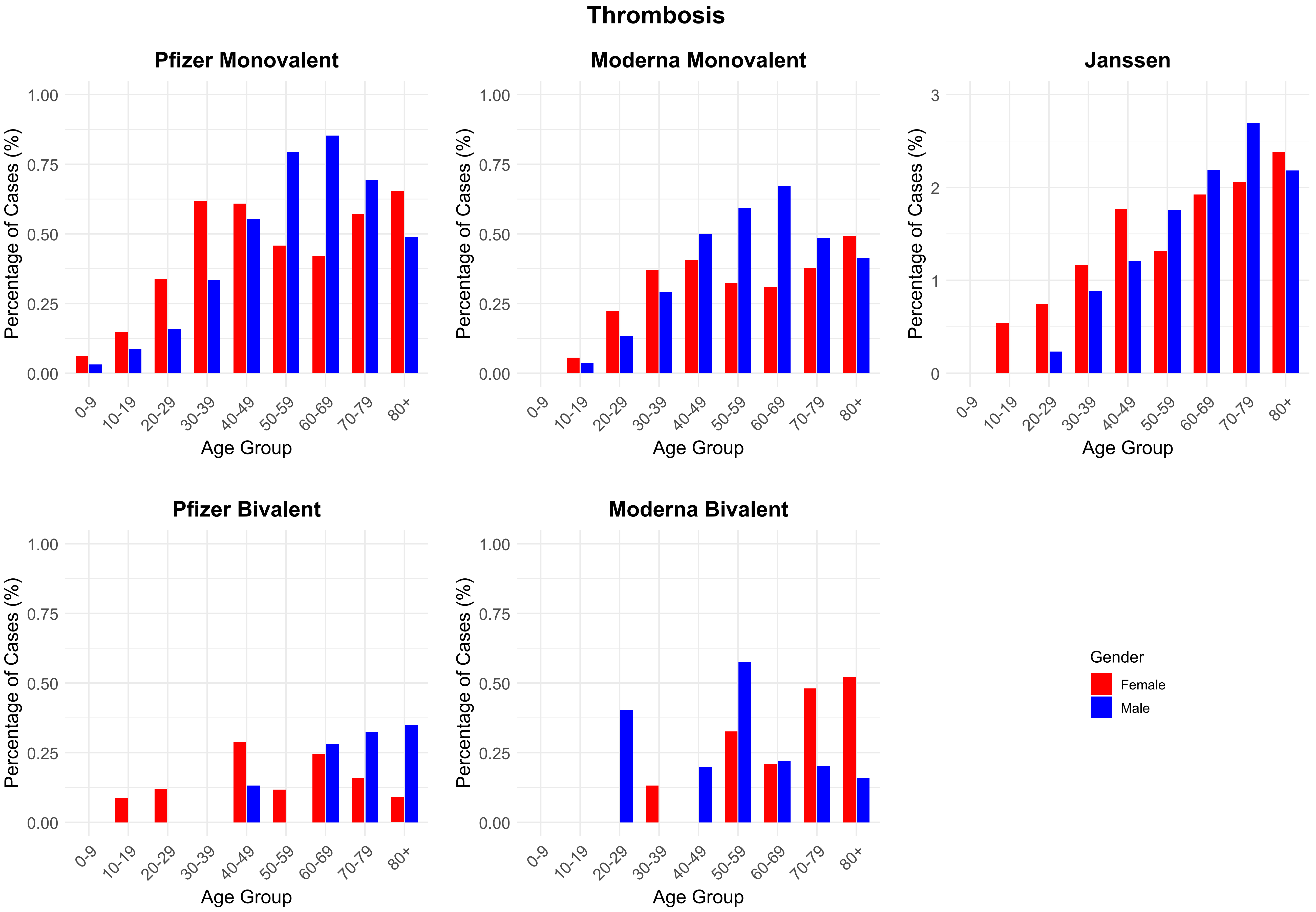

Thrombosis
Pfizer Monovalent
Moderna Monovalent
Janssen
Pfizer Bivalent
Moderna Bivalent
Percentage of Cases (%)
Age Group
0-9
10-19
20-29
30-39
40-49
50-59
60-69
70-79
80+
0.00
0.25
0.50
0.75
1.00
0
1
2
3
Gender
Female
Male

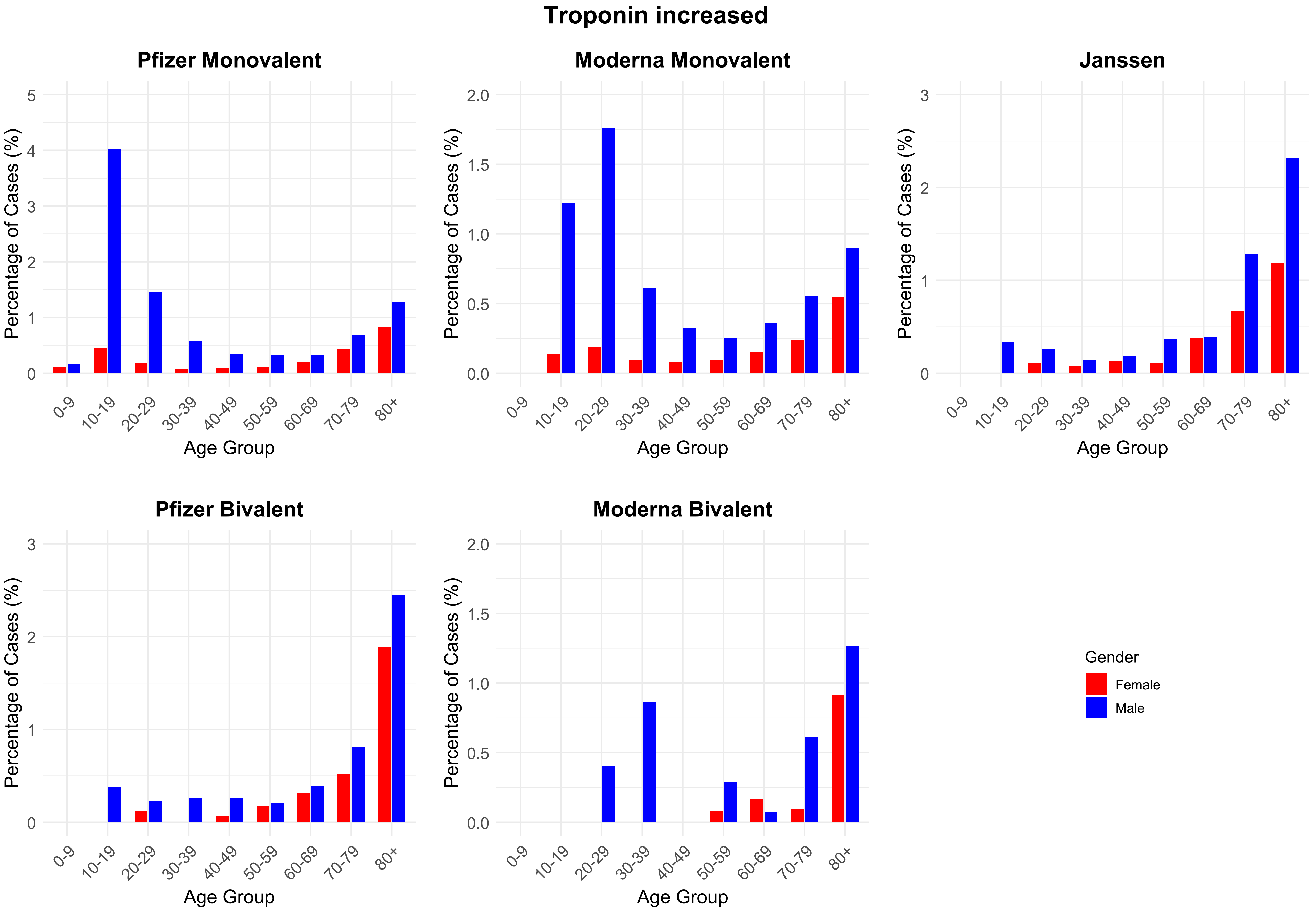
Troponin increased
Pfizer Monovalent
Moderna Monovalent
Janssen
Pfizer Bivalent
Moderna Bivalent
Percentage of Cases (%)
Age Group
0-9
10-19
20-29
30-39
40-49
50-59
60-69
70-79
80+
Gender
Female
Male

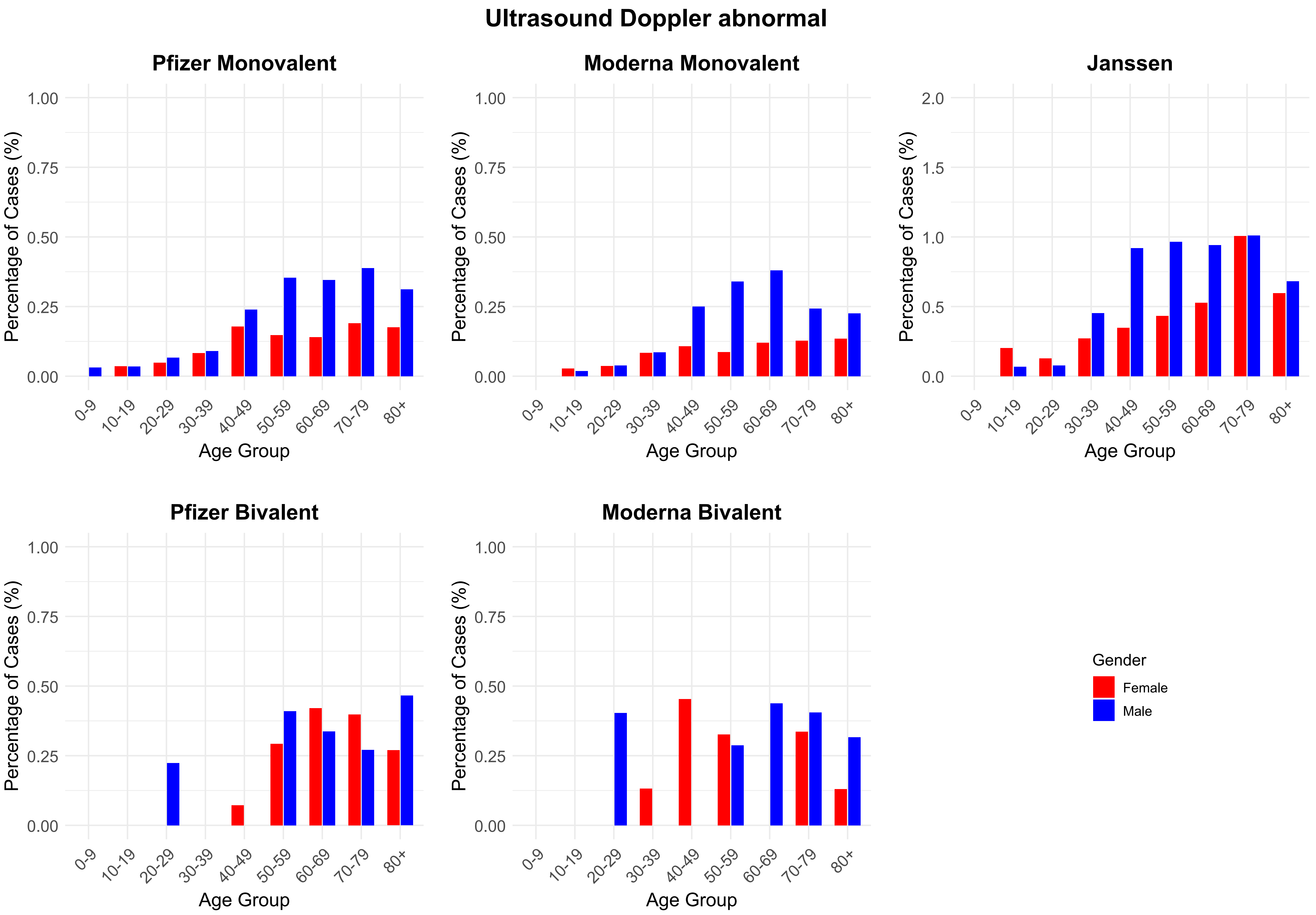

Ultrasound Doppler abnormal
Pfizer Monovalent
Moderna Monovalent
Janssen
Pfizer Bivalent
Moderna Bivalent
Percentage of Cases (%)
Age Group
0-9
10-19
20-29
30-39
40-49
50-59
60-69
70-79
80+
0.00
0.25
0.50
0.75
1.00
0.0
0.5
1.0
1.5
2.0
Gender
Female
Male

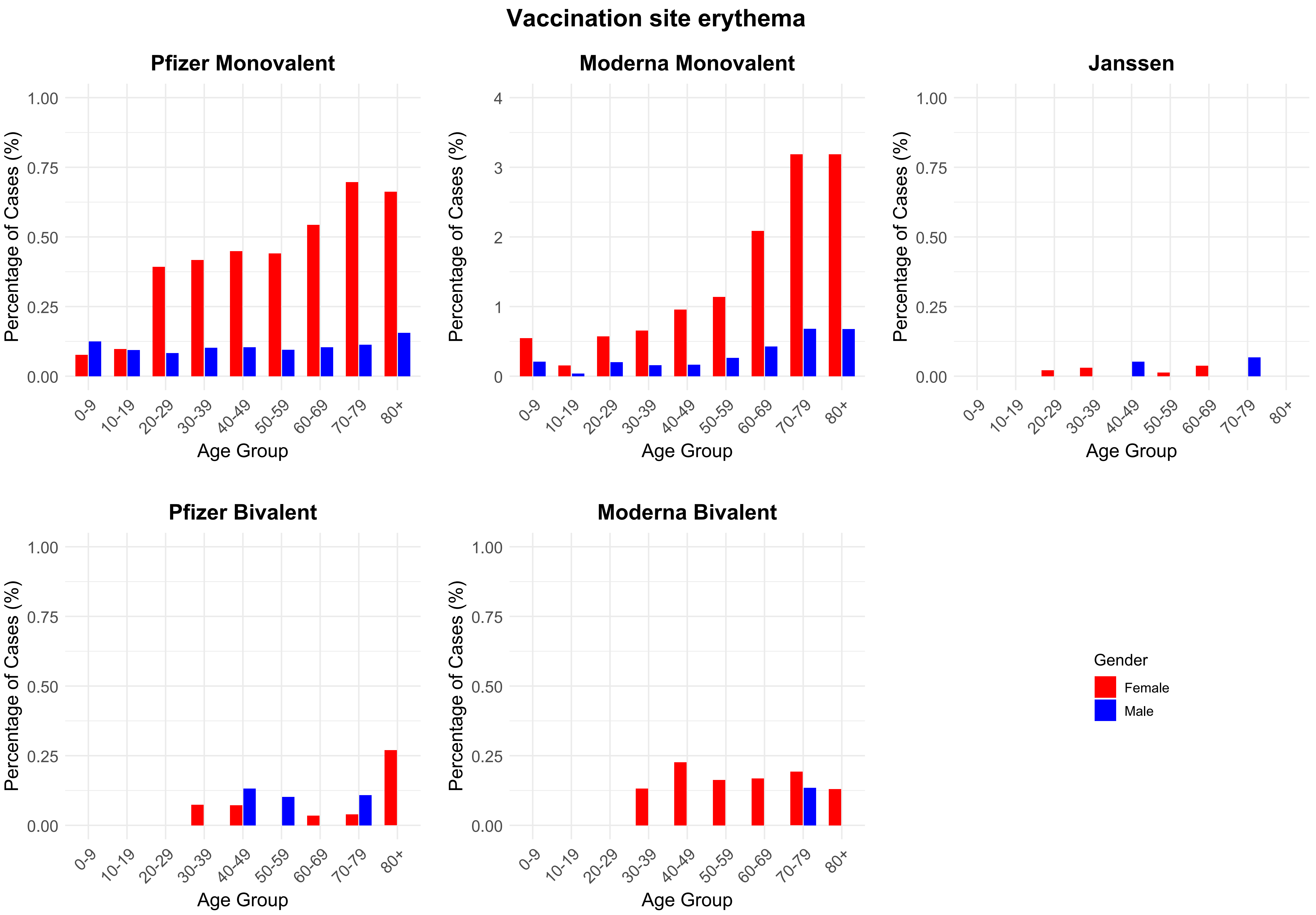

Vaccination site erythema
Pfizer Monovalent
Moderna Monovalent
Janssen
Pfizer Bivalent
Moderna Bivalent
Percentage of Cases (%)
Age Group
0-9
10-19
20-29
30-39
40-49
50-59
60-69
70-79
80+
0.00
0.25
0.50
0.75
1.00
0
1
2
3
4
Gender
Female
Male

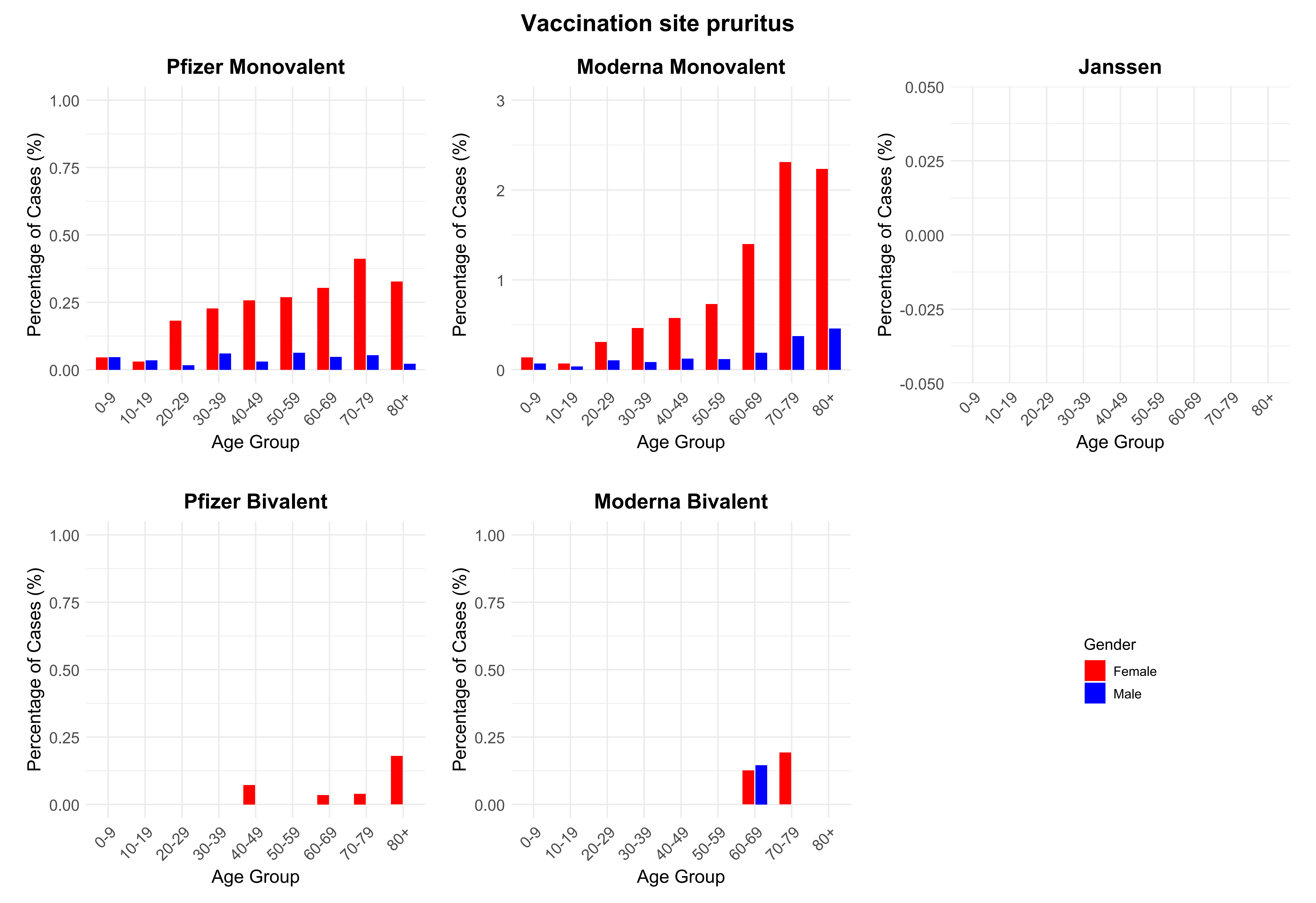
Vaccination site pruritus
Pfizer Monovalent
Moderna Monovalent
Janssen
Pfizer Bivalent
Moderna Bivalent
Percentage of Cases (%)
Age Group
0-9
10-19
20-29
30-39
40-49
50-59
60-69
70-79
80+
1.00
0.75
0.50
0.25
0.00
3
2
1
0
0.050
0.025
0.000
-0.025
-0.050
Gender
Female
Male

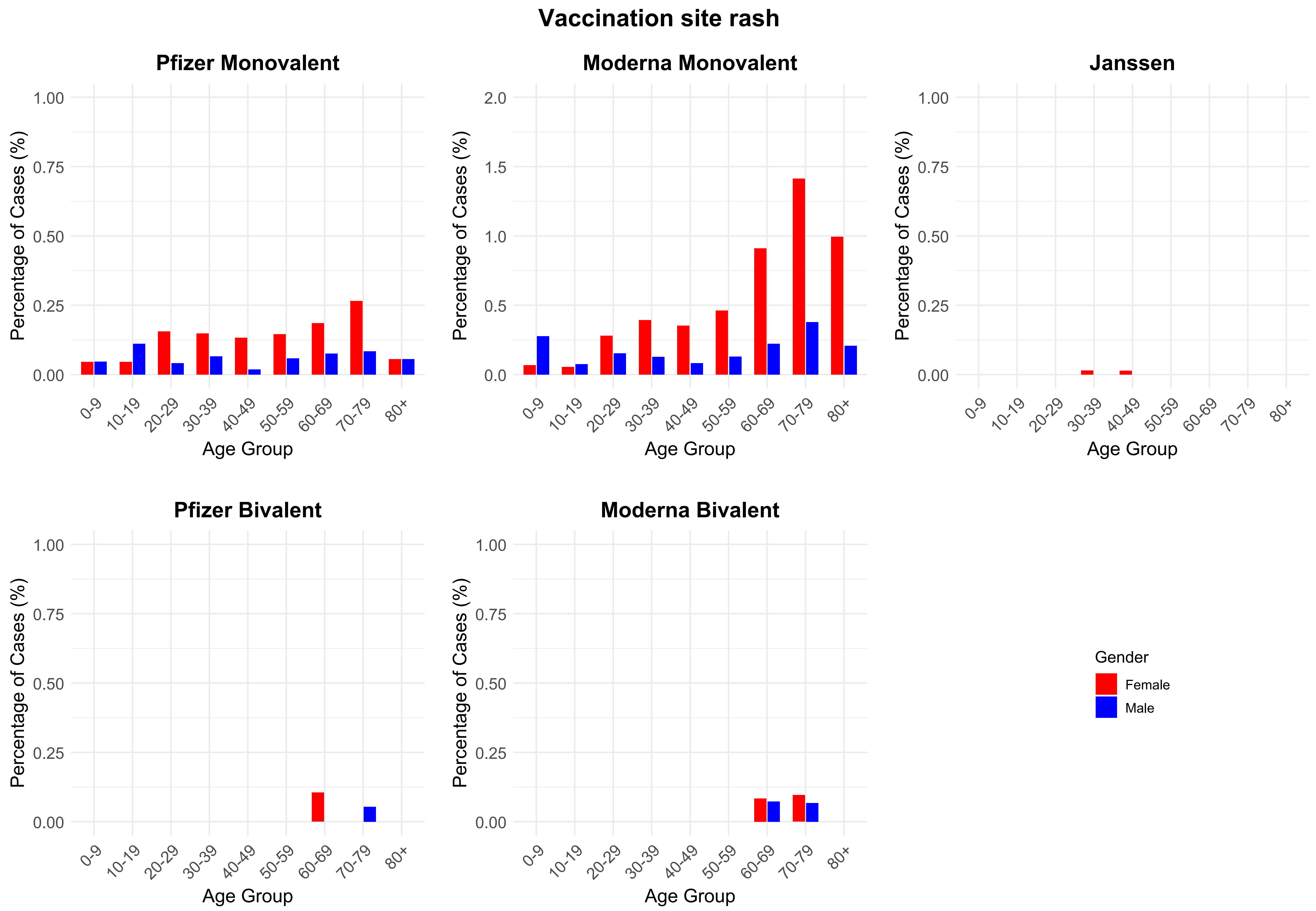

Vaccination site rash
Pfizer Monovalent
Moderna Monovalent
Janssen
Pfizer Bivalent
Moderna Bivalent
Percentage of Cases (%)
Age Group
0-9
10-19
20-29
30-39
40-49
50-59
60-69
70-79
80+
0.00
0.25
0.50
0.75
1.00
0.0
0.5
1.0
1.5
2.0
Gender
Female
Male

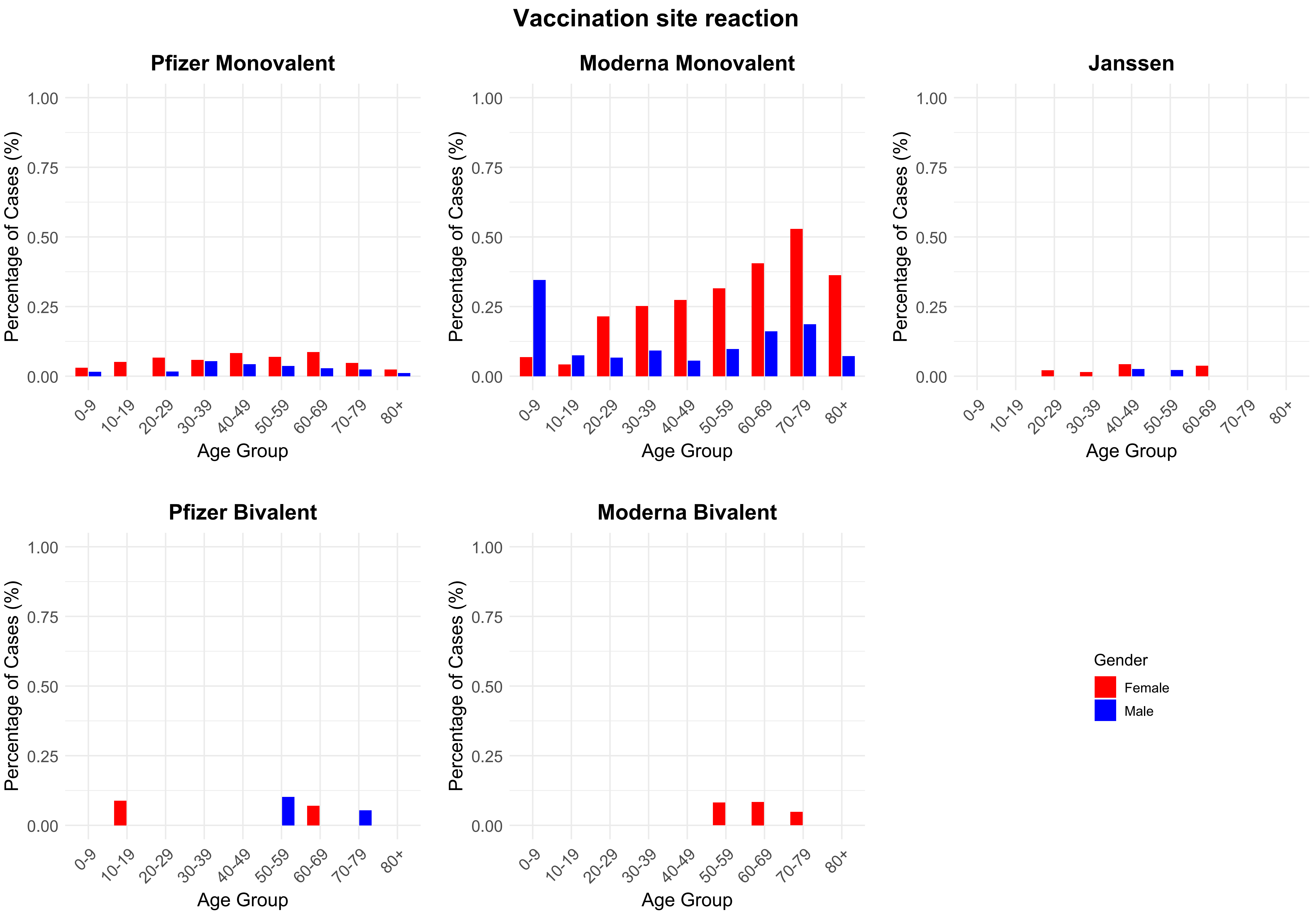
Vaccination site reaction
Pfizer Monovalent
Moderna Monovalent
Janssen
Pfizer Bivalent
Moderna Bivalent
Percentage of Cases (%)
1.00
0.75
0.50
0.25
0.00
0-9
10-19
20-29
30-39
40-49
50-59
60-69
70-79
80+
Age Group
Gender
Female
Male

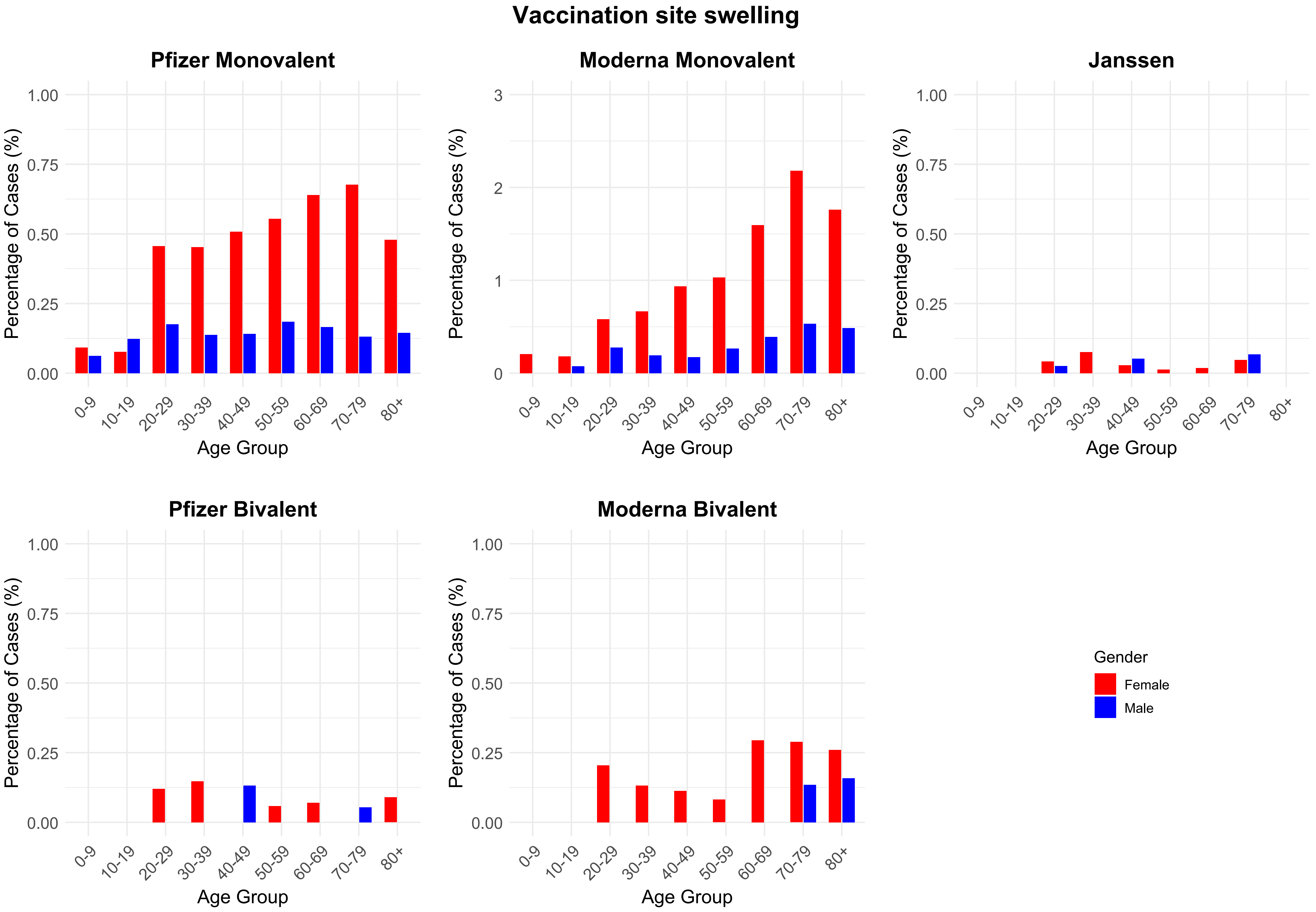

Vaccination site swelling
Pfizer Monovalent
Moderna Monovalent
Janssen
Pfizer Bivalent
Moderna Bivalent
Percentage of Cases (%)
Age Group
0-9
10-19
20-29
30-39
40-49
50-59
60-69
70-79
80+
0.00
0.25
0.50
0.75
1.00
0
1
2
3
Gender
Female
Male

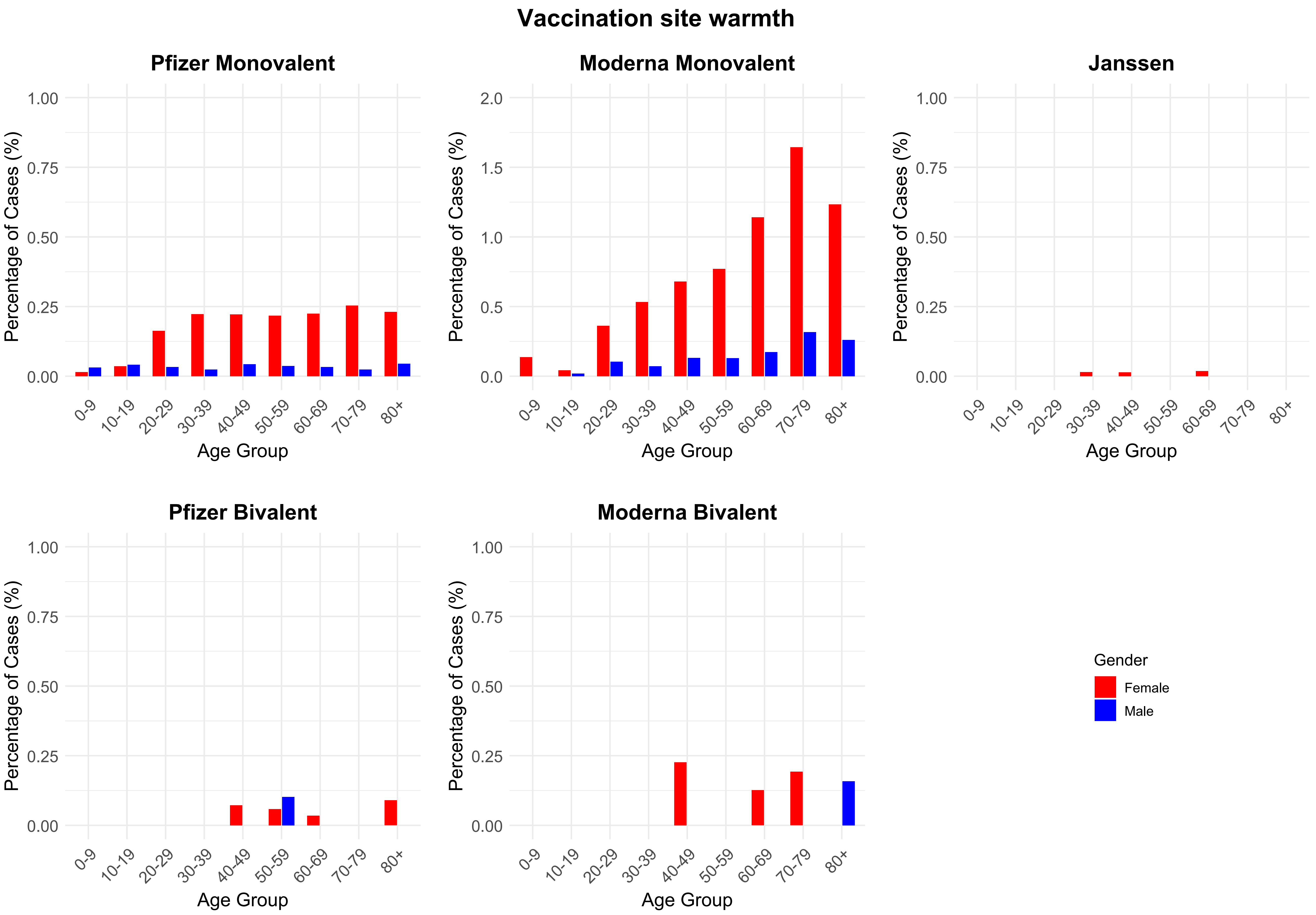
Vaccination site warmth
Pfizer Monovalent
Moderna Monovalent
Janssen
Pfizer Bivalent
Moderna Bivalent
Percentage of Cases (%)
Age Group
0-9
10-19
20-29
30-39
40-49
50-59
60-69
70-79
80+
0.00
0.25
0.50
0.75
1.00
0.0
0.5
1.0
1.5
2.0
Gender
Female
Male
